# Differences in evolutionary history translate into differences in invasion success of alien mammals in South Africa

**DOI:** 10.1002/ece3.1031

**Published:** 2014-04-30

**Authors:** Kowiyou Yessoufou, Jephris Gere, Barnabas H Daru, Michelle van der Bank

**Affiliations:** 1 Department of Environmental Sciences, University of South Africa, Florida campusFlorida, 1710, South Africa; 2 African Centre for DNA Barcoding, University of Johannesburg, APK CampusAuckland Park, 2006, South Africa; 3 Department of Biological Sciences, Bindura University of Science EducationBindura, Private Bag, 1020, Zimbabwe

**Keywords:** Biological invasion, evolutionary distinctiveness, invasion management, life-history traits

## Abstract

Attempts to investigate the drivers of invasion success are generally limited to the biological and evolutionary traits distinguishing native from introduced species. Although alien species introduced to the same recipient environment differ in their invasion intensity – for example, some are “strong invaders”; others are “weak invaders” – the factors underlying the variation in invasion success within alien communities are little explored. In this study, we ask what drives the variation in invasion success of alien mammals in South Africa. First, we tested for taxonomic and phylogenetic signal in invasion intensity. Second, we reconstructed predictive models of the variation in invasion intensity among alien mammals using the generalized linear mixed-effects models. We found that the family Bovidae and the order Artiodactyla contained more “strong invaders” than expected by chance, and that such taxonomic signal did not translate into phylogenetic selectivity. In addition, our study indicates that latitude, gestation length, social group size, and human population density are only marginal determinant of the variation in invasion success. However, we found that evolutionary distinctiveness – a parameter characterising the uniqueness of each alien species – is the most important predictive variable. Our results indicate that the invasive behavior of alien mammals may have been “fingerprinted” in their evolutionary past, and that evolutionary history might capture beyond ecological, biological and life-history traits usually prioritized in predictive modeling of invasion success. These findings have applicability to the management of alien mammals in South Africa.

## Introduction

What drives invasion success of aliens in new environments is likely the most commonly asked question in invasion biology. One theory suggests that the differences in life-history traits between native and alien are key drivers of invasion success. In plants for instance, traits such as habits (life-forms), seed weight, and leaf mass per area correlate with invasion success (Gleason and Cronquist 1991; Pyšek and Richardson 2007; Reich et al. 2007). For mammals, recent studies identified body size as linked to invasiveness (Jeschke and Strayer 2006; Sol et al. 2008; Zalewski and Bartoszewicz 2012). However, many other studies also indicate that life-history traits do not always predict invasion ability, and that identifying those traits is even a more challenging task (Kolar and Lodge 2001; Schaefer et al. 2011; Fautley et al. 2012). Several theories have been developed to explain invasion success: Multiple Introduction Hypothesis, Enemy Release Hypothesis, Shifting Defense Hypothesis, and Evolution of Increased Competitive Ability Hypothesis. However, the importance of species evolutionary history is not explicitly highlighted in these theories.

An alternative and major contribution to our understanding of invasion success is termed “Darwin naturalization hypothesis” (hereafter referred to as Darwin's hypothesis). Darwin argued that the relatedness (phylogenetic) between native and alien species is a key predisposing factor, such that, aliens that have no closely related species in new environments are more likely to establish and invade the recipient communities (Darwin 1859). Although Darwin's hypothesis does explain the invasion success of some aliens in many environments (Strauss et al. 2006; Jiang et al. 2010; Schaefer et al. 2011), its explanatory power has also been discounted in many others (Cahill et al. 2008; Diez et al. 2008; Maitner et al. 2011; Bezeng et al. 2013). Under Darwin's hypothesis, we expect aliens to be evolutionarily distinct from natives. The evolutionary distinctiveness of species can be assessed using “species evolutionary distinctiveness” metric (ED; Isaac et al. 2007). As such, under Darwin's hypothesis, aliens should have, on average, greater ED value than natives. In this study, we are investigating the drivers of the variation in invasion success of alien mammals in South Africa. Our approach is therefore different from the typical test of Darwin's hypothesis because we are comparing the phylogenetic relatedness within aliens and not between aliens and natives. Indeed, alien species introduced to the same environment do not necessarily exhibit similar intensity of invasion: some are “strong invaders”, others are “weak invaders” (Hufbauer and Torchin 2007), and others are even noninvasive. What are the underlying factors of such variation is the main research question of this study.

In South Africa, there is an increasing effort toward the establishment of a database of all alien species (plants, animals, micro-organisms, fungi) where aliens are categorized according to their invasion intensity (Data S1). Five categories have been identified, namely, in decreasing order of invasion intensity: “Appendix 1” (species listed as prohibited alien species, i.e., “strong invaders”); “Appendix 2” (species listed as permitted alien species, i.e., noninvasive alien species); “Appendix 3” (species listed as invasive species, i.e., “weak invaders” as opposed to “strong invaders”); “Appendix 4” (species listed as known to be invasive elsewhere in the world but not in South Africa); and “Appendix 5” (species listed as potentially invasive elsewhere in the world). Here, we focus only on mammal alien species and ask: why are introduced alien mammals to South Africa not equally invasive? In other words, what are the correlates of the variation in invasion intensity (Appendix 1–Appendix 5) of alien mammals in South Africa?

Although invasive alien animals of South Africa have received comparatively less attention than invasive alien plants in the past, a recent study in Europe indicated that the negative impacts of invasive animals might be equal or even greater than those of plants (Vilà et al. 2010). The negative impacts of alien animals include herbivory (overgrazing or overbrowsing), diseases transmission to wildlife and to human, and hybridization with native animals, which has been showed to lead to serious decline of local population and even to extinction of native species (Hughes 1996; Munoz-Fuentes et al. 2007; Genovesi et al. 2012). Animal invaders could also be detrimental to agriculture through the destruction of agricultural landscape (Bertolino and Genovesi 2007; Bertolino and Viterbi 2010). Today, commitment to the study of alien animals in South Africa is increasing (Picker and Griffiths 2011).

The most cost-effective strategy in invasion management is not only to identify potential invasives before they are introduced to new ranges, but also to predict the intensity of their invasion. Adopting such a pre-emptive strategy relies critically on our ability to understand the factors that underlie invasion success and to predict potential invaders (Cadotte et al. 2009). Categorizing alien mammals based on the intensity of invasion success (strong invaders vs. weak invaders vs. noninvasive), we first tested for phylogenetic signal in invasion intensity. We then constructed alternative models of invasion intensity to identify the potential drivers of the observed variation, combining mammal phylogenetic distinctiveness, biological and ecological factors.

## Methods

### Categorization of alien mammals in South Africa

Alien species are grouped into five categories or Appendices (Data S1) based on their invasion intensity ranging from Appendix 1 to Appendix 5. Appendix 1 includes “species listed as prohibited alien species”, that is, all aliens introduced to South Africa that have been strongly detrimental owing to their high invasion intensity (“strong invaders”; Hufbauer and Torchin 2007; Kumschick et al. 2011). We referred to these species as “prohibited species”. In contrast, other introduced species categorized as Appendix 2 do not show so far any invasion ability and are therefore labeled as “species listed as permitted alien species” (“noninvasive aliens”). We referred to these species as “permitted species” as opposed to “prohibited species.” The third category, i.e., Appendix 3 labeled as “species listed as invasive species” includes all species that are invasive but whose invasion intensity and impacts are less than those of the Appendix 1 (“weak invaders”; Hufbauer and Torchin 2007). We referred to this category as “invasive species.” Appendices 4 and 5 include, respectively, “species listed as known to be invasive elsewhere in the world” and “species listed as potentially invasive elsewhere in the world.”

### Data collection

We included in this study only species that are alien in South Africa and present in PanTHERIA database (Jones et al. 2009). From this worldwide database, we retrieved 38 life-history variables characterizing the ecology, biology, and societal life of mammals (Table S1).

In the current checklist of alien mammals of South Africa, there are 20 species listed in Appendix 1, eight in Appendix 2 and 68 in Appendix 3 (Table S1; Data S1). There is no species listed at the moment in Appendix 4 and only one species is currently under Appendix 5. For the purpose of data analysis, we replaced the species *Castor* spp. listed under Appendix 1 with *Castor canadensis* for which data are available in PanTHERIA. Also, all hybrids found in Appendices (e.g., *Connochaetes gnou* × *C. taurinus taurinus*) were removed from the analysis as well as all species listed in Appendices but missing in the PanTHERIA database. We did not include the single species listed under Appendix 5. In total, alien mammals analyzed in this study include: Appendix 1 (prohibited = 19 species), Appendix 2 (permitted = 7 species), and Appendix 3 (invasive = 51 species).

### Data analysis

We converted invasive status of all alien species into binary traits: “prohibited” (Appendix 1) versus nonprohibited (Appendices 2 + 3). We then tested for taxonomic selectivity in invasion intensity assessing whether there were more or less “prohibited” species in some taxa (families and orders) than expected by chance. For this purpose, we estimated the proportion of prohibited species (observed proportion) in each family and order. If *n* is the total number of prohibited species in the dataset, we generated from the dataset 1000 random assemblages of *n* species each. For each of the random assemblages, we calculated the proportion of prohibited species (random proportion). The significance of the difference between the observed and the mean of the 1000 random proportions was tested based on 95% confidence intervals (CI).

We also tested whether the taxonomic selectivity, if any, translates into phylogenetic selectivity in invasion intensity (prohibited vs. nonprohibited) using Fritz and Purvis' (2010) D statistic implemented in the R package “Caper” (Orme et al. 2012). The D statistic provides an estimate of phylogenetic signal for binary traits and compares the observed D value not only with that of a random shuffle of trait values at the tips of a phylogeny but also with that of a Brownian motion (BM) model. *D* = 1 is indicative of a pattern of no phylogenetic structure in the trait considered (here invasion intensity); *D* = 0 corresponds to a BM model; *D* < 0 when traits are highly conserved, that is, when phylogenetically closely related species tend to share similar invasion status (prohibited or nonprohibited). A value of *D* > 1 suggests that the trait is phylogenetically over-dispersed.

To further analyze the phylogenetic structure within invasion categories, we applied two phylogenetic metrics commonly used in community ecology, that is, the net relatedness index (NRI) and nearest taxon index (NTI) (Webb et al. 2002). NRI and NTI values are the results of the comparison, respectively, of the observed mean phylogenetic distance (MPD) and mean nearest taxon distance (MNTD) in each invasion category to the random values of MPD and MNTD. These random MPD and MNTD were calculated based on the null model “phylogeny.pool” (R package “Picante 1.2.”) where species within each category were drawn randomly 1000 times from the phylogeny with equal probability (Kembel et al. 2010). NRI and NTI were calculated for “prohibited species,” “permitted species,” “invasive species,” and “nonprohibited” (“permitted species” + “invasive species”).

Furthermore, using the mammalian tree of life (Bininda-Emonds et al. 2007), we calculated two metrics characterising species uniqueness: species evolutionary distinctiveness (ED; Isaac et al. 2007) and species evolutionary ages. Species ages were determined as the length of terminal branches (BL) that connect each species to the tree. Both BL and ED characterize how species differ in their evolutionary history with the difference that, unlike BL, ED accounts for evolutionary relationships deeper in the phylogenetic tree (Isaac et al. 2007). We compared BL and ED within pairs of invasive categories (prohibited, permitted, invasive, and nonprohibited) using the Wilcoxon rank sum test.

Finally, we reconstructed alternative models of invasion intensity, which was coded as a binary response variable (1 = prohibited and 0 = nonprohibited). Each model was assigned a binomial error distribution and a logit link function. To account for taxonomic selectivity found in invasion intensity (see Results section), we fitted generalized linear mixed-effect models (GLMM) to the data using the glmer function implemented in the R package lme4 (Bates et al. 2013). Our fixed effects were life-history traits as well as ED and BL (Table S1). Family was used as random effect. We identified all the significant correlates of the variation in invasion intensity. Then, we reconstructed pairwise plots of all these correlates against each other to identify highly correlated pairs (Figure S1). In each pair, we excluded one correlate (the least significant) to reduce redundancy of significant correlates of invasion success of alien mammals.

## Results

**Figure 1:**
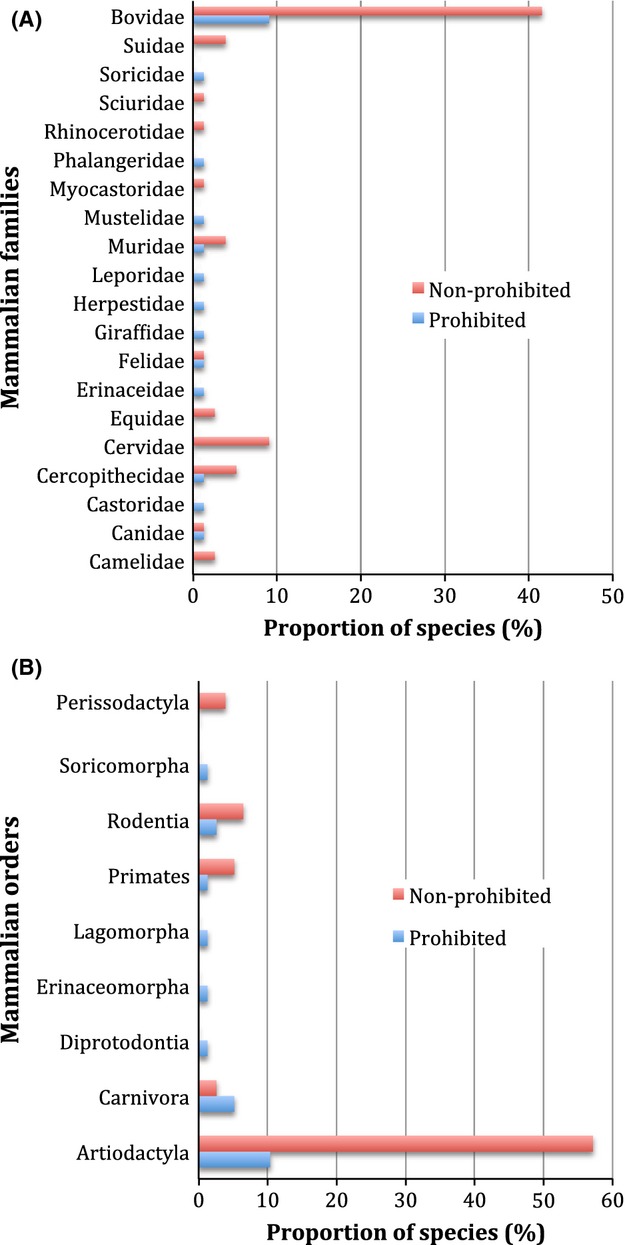
Taxonomic distribution of invasion success of alien mammals in South Africa: (A) Patterns across families and (B) Patterns across orders. Proportion of species was assessed as number of prohibited (strong invaders) and nonprohibited species in a taxon divided by the total number of species assessed within that taxon.

All alien mammals included in this study belong to 20 families and nine orders (Table S1). We found that one family – Bovidae – and the corresponding order – Artiodactyla – contained more prohibited species than expected by chance (observed proportion = 10.39; mean random proportion = 4.31; CI = 2–6.66). In contrast, no single prohibited species was found in seven families (Suidae, Sciuridae, Rhinocerotidae, Myocastoridae, Cervidae, Equidae, and Camelidae) and one order (Perissodactyla) (Figure 1). This is an indication of a taxonomic selectivity in invasion intensity. However, testing for phylogenetic selectivity using the D-statistics, the estimated D value was not significantly different from *D* = 1 (D estimated = 0.82, *P* = 0.198), but departed significantly from the expectation under a BM model (*P* = 0.008). These findings indicate that the taxonomic selectivity found do not translate into phylogenetic signal in invasion intensity.

Using NRI and NTI metrics, we further tested for phylogenetic structure in “prohibited” and “nonprohibited” species. We found evidence for a phylogenetic patterning in only nonprohibited species: Prohibited (NRI = −2.34, *P* = 0.99^ns^; NTI = −2.71, *P* = 0.99^ns^); nonprohibited (NRI = 2.61; *P* = 0.007**; NTI = 2.30, *P* = 0.012*). We now broke down the nonprohibited species into “permitted” and “invasive” and recalculated the NRI and NTI values. We found evidence for phylogenetic clustering only in “invasive” category: Permitted (NRI = −0.20, *P* = 0.53^ns^; NTI = 0.26; *P* = 0.41^ns^) and Invasive (NRI = 2.70; *P* = 0.007**; NTI = 1.91; *P* = 0.03*). This indicates that the phylogenetic structure found in nonprohibited species is driven by species within the “invasive” category.

**Figure 2:**
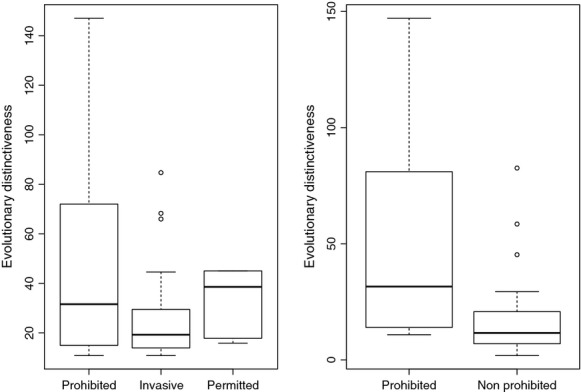
Comparison of evolutionary distinctiveness of alien mammals in South Africa across invasion categories. Prohibited = strong invaders; permitted = noninvasive alien; invasive = alien mammals with invasion intensity lower than that of prohibited.

When we compared prohibited versus nonprohibited species based on their evolutionary ages (BL), we found that the terminal branches of prohibited species are no longer than those of nonprohibited (median BL = 11.3 Myrs vs. 11.65 Myrs; Wilcoxon sum ranked test, *W* = 639, *P* = 0.30^ns^), indicating that species recent evolutionary history do not predispose one to high invasion intensity than other. However, when accounting for their evolutionary history deeper in the tree by comparing ED values across invasion categories, we found that prohibited species were clearly evolutionarily distinct from nonprohibited species (median ED = 31.59 Myrs vs. 11.65 Myrs; *W* = 910, *P* < 0.0001***). Nevertheless, neither prohibited versus invasive (median ED = 31.59 Myrs vs. 19.26 Myrs; *W* = 625, *P* = 0.06^ns^), prohibited versus permitted (median ED = 31.59 Myrs vs. 38.59 Myrs; *W* = 66, *P* = 1^ns^) nor permitted versus invasive (median ED = 38.59 Myrs vs. 19.26 Myrs; *W* = 99.5, *P* = 0.06^ns^) showed significant differences in their evolutionary distinctiveness (Figure 2).

Finally, we tested the predictive power of life-history traits on invasion intensity of alien mammals. Of all 38 traits tested, only four traits were identified as significant (although marginally) correlates of invasion intensity. These include: latitude (minimum latitudinal ranges, *P* = 0.03*; median latitudinal ranges, *P =* 0.019*; maximum latitudinal ranges, *P* = 0.025*), gestation length (*P* = 0.013*), social group size (*P* = 0.039*), and human population density change (*P* = 0.014*) (Table 1). All parameters related to latitude are highly correlated (Figure S1), suggesting that any of them can be used as a significant predictor of invasion intensity. Latitude shows positive correlation with invasion intensity, but gestation length and human population changes show negative correlation (Table 1). In contrast to life-history traits, species evolutionary distinctiveness provided stronger positive explanatory power of invasion intensity (Table 1): BL (*P* = 0.008**)

**Table 1:** Model coefficients for the generalized linear mixed-effect models (GLMM) of invasion intensity of alien mammals in South Africa. Invasion intensity was converted into binary data (prohibited vs. nonprohibited; see text) before fitting GLMM with binomial errors

Predictive variables	Estimate	Std. Error	Z value	*P* value
Evolutionary distinctiveness	0.034	0.012	2.73	0.006**
Minimum latitudinal range	0.027	0.012	2.16	0.030*
Median latitudinal range	0.029	0.012	2.34	0.019*
Maximum latitudinal range	0.024	0.010	2.243	0.025*
Gestation length	−0.007	0.003	−2.474	0.013*
Social group size	0.051	0.024	2.067	0.039*
Human population density change	−15.425	6.314	−2.443	0.014*

The number of stars indicates the level of significance.

## Discussion

Because some alien species are “strong invaders” while others are weak (Kumschick et al. 2011), and others even fail to establish in new ranges (Rodriguez-Cabal et al. 2009) even when they are introduced to similar environmental conditions, we ask: why aliens are not equally invasive in new ranges? Given the negative impacts of invasive alien species on native (Pimentel 2001; Courchamp et al. 2003; White et al. 2008; Forsyth et al. 2010; Nunez et al. 2010), a broader understanding of what drives the variation in invasion success is not only necessary but also critical for a better invasion management. In this study, we focus on alien mammals introduced to South Africa. To investigate the question, we first tested for taxonomic and phylogenetic signal in invasion intensity, expecting some taxonomic groups to contain an unusual proportion of strong invaders (taxonomic selectivity) and species within some particular clades to share similar invasion success (phylogenetic selectivity). We found evidence for taxonomic selectivity as the family Bovidae in the order Artiodactyla contained more “prohibited” species or more “strong invaders” than expected by chance whilst prohibited species are not found, at least for now, in other taxa. Previous studies have also identified Artiodactyla as comprising an unusual proportion of invaders (e.g., Clout and Russell 2008), thus giving support to the taxonomic selectivity found in this study. However, this taxonomic signal did not translate into phylogenetic selectivity. Our test rather indicates that, from a phylogenetic perspective, invasion intensity is distributed randomly across the tips of the phylogeny. This finding discounts a priori the potential of phylogeny in predicting variation in invasion success of alien mammals. Nonetheless, our finding that “nonprohibited species” (“permitted” + “invasive”) are more phylogenetically related than expected by chance indicates that phylogeny might still play a role in driving variation in invasion ability. Looking into the “nonprohibited” category, we only found a phylogenetic structure in “invasive species,” indicating that the phylogenetic patterning found within nonprohibited species is more likely driven by “invasive species,” and that the overall lack of phylogenetic signal might be driven by “prohibited species.”

Given the phylogenetic structure found in nonprohibited species, we expect species evolutionary history to be a driving force of invasion success. We evaluate this hypothesis comparing species evolutionary ages and distinctiveness. We found that species recent evolutionary history as measured by their ages (terminal branch length) is not important driver. However, when accounting for the differences toward the origin of the tree, we found that prohibited species (strong invaders) were more evolutionarily distinct (greater ED value) than nonprohibited, giving support to the phylogeny as a potential predicting tool of the variation in invasion success of alien mammals. In animal kingdom, mammals are known to have stronger ability to establish viable and sustainable populations in new environments (Clout and Russell 2008) through a relatively easy capacity to adjust their ecology and biology (Lee and Gelembiuk 2008; Van Kleunen et al. 2010; Fautley et al. 2012; Zalewski and Bartoszewicz 2012). Their adaptation and spread generally lead to major negative impacts (Pimentel 2001; Courchamp et al. 2003; Hemami et al. 2005; White et al. 2008; Feldhamer and Demarais 2009; Senn and Pemberton 2009; Forsyth et al. 2010; Nunez et al. 2010). A better control of invasive species would rely fundamentally on our ability to anticipate actions and predict future potential invaders. Such predictive power is contingent upon our understanding of correlates of invasion (Fautley et al. 2012). Uncovering those drivers is, however, a complex task given that different factors play important roles at different stages of invasion process (Fautley et al. 2012). Therefore, efforts should be maximized in investigating factors associated with species success at each stage of the invasion process (Fautley et al. 2012). However, that is not our objective in this study. Here, we focus on alien mammals that are already established in South Africa. We are particularly interested in what could explain the variation in their invasion intensity. We investigated multiple factors combining life-history traits and evolutionary-related metrics. Among life-history traits, we found that latitudinal ranges, social group size, and litter size are positively associated with the variation in invasion success of alien mammals, whereas the gestation length and human population density change correlate negatively.

How can we explain the positive correlations? We found that invasion intensity is greater at high latitude. This was also recently found for the females of American mink (*Neovison vison*), a mammalian species of the family Mustelidae (Zalewski and Bartoszewicz 2012). One explanation is that, at high latitude, the body size of the female of American mink is reduced as a result of reduction in food requirements in favor of reproduction ability (Erlinge 1979; Moors 1980). Such increase in reproduction success at high latitude will elevate the risk of invasion success (Zalewski and Bartoszewicz 2012), thus justifying the positive correlation we found between latitude and invasion intensity in this study. The positive correlation between social group size and invasion intensity indicates that species living in communities of high number of individuals have high invasion capacity. An explanation could be linked to reproductive rate. Indeed, a community of living organisms generally includes both sexes, thus facilitating breeding. Another plausible explanation is that species living in groups defend altogether against predators. Such defense mechanisms would enhance their survival, and also their establishment and spread.

What about negative correlations? We found that, in areas where human population density increases, mammals have low invasion intensity. We link this negative correlation to human–animal conflicts such that, an increase in human population, might lead to a disproportionate loss of animal habitats, thus depressing animal survival. Further, our results also indicate that longer gestation period is associated with low invasion intensity. This could be expected as long gestation generally results in low litter size, hence low invasion ability.

In contrast to the life-history traits that are only marginally significant predictors, ED shows stronger positive predictive power, indicating that alien mammals that are more evolutionarily isolated have greater invasion ability. Why this? ED captures the evolutionary past of species that makes one species distinct from the other (Redding and Mooers 2006; Isaac et al. 2007). Species evolutionary history is predicted to capture useful feature diversity (Faith 1992; Crozier 1997; Forest et al. 2007; Faith et al. 2010) but might also capture unwanted features that predispose, for instance, species to greater invasion success. In addition, functional diversity correlates with species diversity but more strongly with evolutionary history (Forest et al. 2007; Faith et al. 2010), suggesting that evolutionary history would capture species behaviors, for example, their invasion ability, beyond the predictive power of species *per se* (Redding et al. 2008). As such, evolutionary history would explain the invasion success better than life-history traits. This is exactly what we found in this study.

Invasive species are considered one of the three greatest threats to global biodiversity (Walker and Steffen 1997; Allendorf 2003), and in-conjunction threats with the ongoing climate change may be further amplified. Even currently noninvasive alien species (e.g., permitted species) could become invasive under new climate regimes (Willis et al. 2010) and therefore pose serious economical and ecological problems in the future (Williamson 1996; Mack et al. 2000; Pimentel et al. 2005). As such, there is an urgent need for a continued commitment to better understand the factors predicting invasion success, if we are to prevent and manage future invasion successfully. Invasion success is a result of a long process comprising four stages: transport, introduction, establishment, and spread (Kolar and Lodge 2001). Traditionally, invasion success of aliens is thought to be driven by three major factors including species life-history characteristics (ecology, biology, etc.), the characteristics of recipient communities (presence or absence of alien congeneric species), and the introduction event (propagule size, frequency of the introduction, etc.) (Sol et al. 2008). The identification of useful life-history traits linked to invasion success may be complex as no single trait can correlate with all four stages of invasion process (Fautley et al. 2012). Further, the survival ability of aliens in recipient areas depends on their competitive ability with native species, and this might be favored by high propagule pressure. Our results indicate that the variation in invasion success of alien mammals is “fingerprinted” in their evolutionary past, rather than simply predictable using life-history data. This suggests that mainstreaming evolutionary information into the various programmes of early detection mechanisms of alien species in South Africa is necessary for a better management of invasion species.

## Supporting Information

**Figure S1.** Pairwise relationships between parameters identified as significant correlates of invasion success in our GLMM analysis.

**Table S1.** Species life-history traits of alien mammals in South Africa (retrieved from PanThera database.

**Data S1.** Categorization of alien species (plants, animals, fungi, micro-organisms) in South Africa. In this study, we focus only on mammals.
