# Supplementary figures and images for: Differences in evolutionary history translate into differences in invasion success of alien mammals in South Africa
Source: Ecol Evol. 2014 Apr 30;4(11):2115–23. doi: 10.1002/ece3.1031 (PMC4201426; doi:10.1002/ece3.1031)

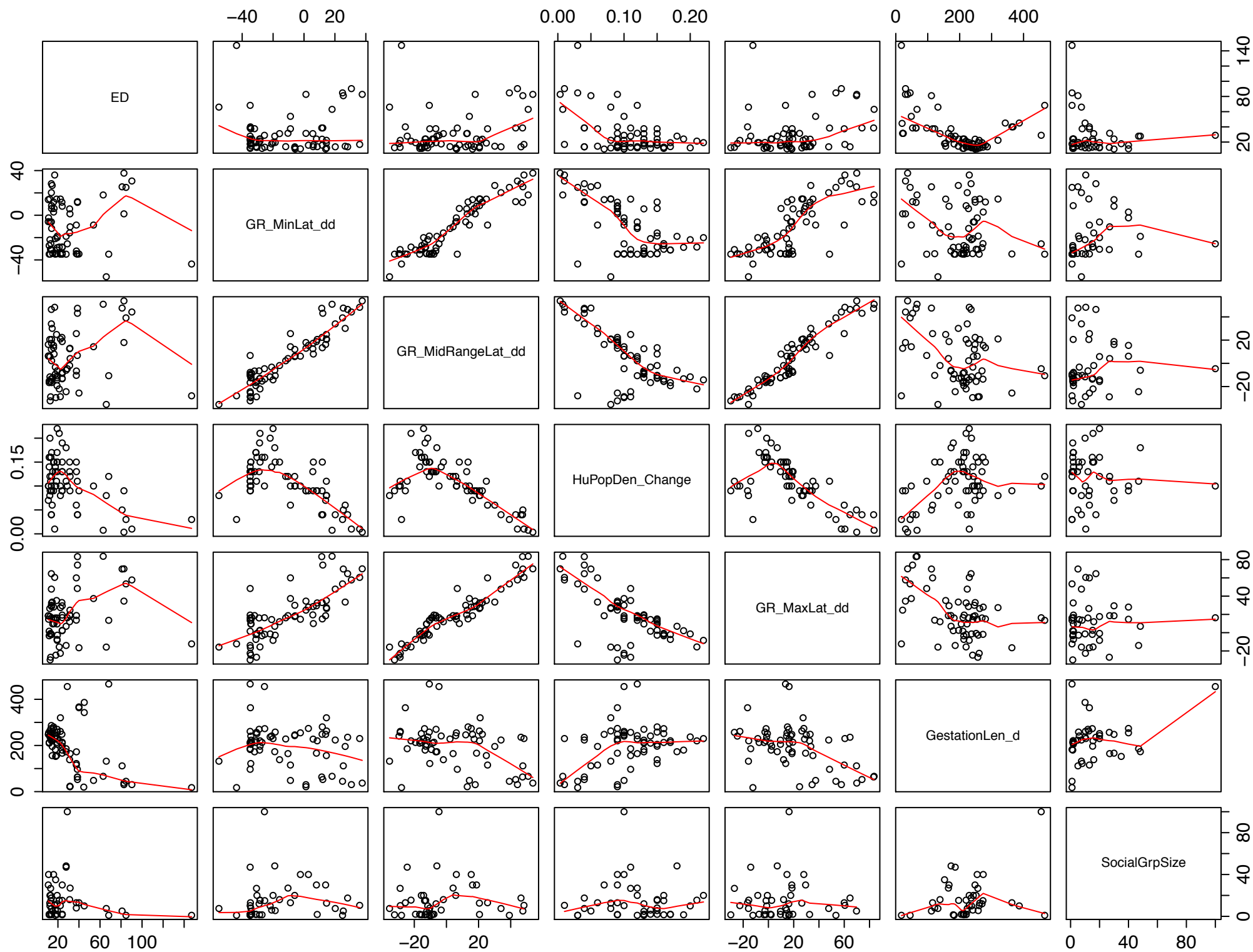

Supplement: Supplementary file 1 — Figure S1. Pairwise relationships between parameters identified as significant correlates of invasion success in our GLMM analysis. [file ece30004-2115-sd1.pdf]
