# Supplementary material for: Differences in evolutionary history translate into differences in invasion success of alien mammals in South Africa
Source: Ecol Evol. 2014 Apr 30;4(11):2115–23. doi: 10.1002/ece3.1031 (PMC4201426; doi:10.1002/ece3.1031)
Supplement: Supplementary file 3 — Data S1. Categorization of alien species (plants, animals, fungi, micro-organisms) in South Africa. In this study, we focus only on mammals. [file ece30004-2115-sd3.pdf]

GOVERNMENT NOTICE

DEPARTMENT OF ENVIRONMENTAL AFFAIRS AND TOURISM

No. **February 2006**

NOTICE UNDER CHAPTER 5 OF THE
NATIONAL ENVIRONMENTAL MANAGEMENT: BIODIVERSITY ACT
(Act No. 10 of 2004)

LISTING OF ALIEN SPECIES AND INVASIVE SPECIES

I, Marthinus van Schalkwyk, Minister of Environmental Affairs and Tourism, after consultation with the Cabinet Cluster, hereby:

(1) in terms of section 67(1) of the Act, list in Appendix 1 the alien species or categories of alien species in respect of which a permit may not be issued;

(2) in terms of section 66(1) of the Act, list in Appendix 2 the alien species or categories of alien species which may be exempted from a risk assessment and permit;

(3) in terms of section 70(1)(a) of the Act, list in Appendix 3 the invasive species to which Chapter 5 of the Act applies nationally;

(4) in terms of section 97(1)(c)(iii) of the Act, list in Appendix 4 the species or categories of alien species for which a permit from the receiving country is required before the species may be exported out of the country; and

(5) in terms of section 97(1)(c)(iii) of the Act, list in Appendix 5 the species or categories of alien species for which there must be compulsory labelling before restricted activities may take place.

M VAN SCHALKWYK
Minister of Environmental Affairs and Tourism

Explanatory note:
The listing of alien species and listed species is done within the Framework for the Assessment and Management of Risk of Alien and Invasive species (Schedule 3 to the National Environmental Management: Biodiversity Act Regulations on Alien and Invasive Species). This requires a science-based approach to the listing process; reqular updating of the lists, and scope to appeal against the listing of species or activities. The economic implicatrions of a potential introduction may also be factored into the decision-making process. The lists that appear here are the initially assessed species. It is anticipated that it will take a further two years to have considered the most key species for possible listing, after which listing will take place on a regular basis, as the need arises. The lists will be reviewed following public comment. Further scope to comment will be made, as the lists are developed. Certain of the taxa lists are still being considered, and will be released at a later stage. This is indicated under the relevant taxa in the lists. Please note that the Author Names for species, and the common names for species in Afrikaans and African languages, will also be given in the final version of these lists.

CONTENTS

Appendix 1: **Species Listed as Prohibited Alien Species**
1.1 Mammals
1.2 Birds
1.3 Reptiles
1.4 Amphibians
1.5 Fishes (Fresh-water)
1.6 Fishes (Marine)
1.7 Other Marine Species
1.8 Invertebrates (Fresh-water)
1.9 Invertebrates (Terrestrial)
1.9.1 Molluscs
1.9.2 Nematodes
1.9.3 Insects
1.9.4 Mites
1.9.5 Spiders
1.10 Plants
1.11 Microbes

Appendix 2: **Species Listed as Permitted Alien Species**
3.1 Mammals
3.2 Birds
3.3 Reptiles
3.4 Amphibians
3.5 Fishes (Fresh-water)
3.6 Fishes (Marine) and other Marine Species
3.7 Invertebrates (Fresh Water)
3.8 Invertebrates (Terrestrial)
3.9 Plants
3.10 Microbes

Appendix 3: **Species Listed as Invasive Species**
4.1 Mammals
4.2 Birds
4.3 Reptiles
4.4 Amphibians
4.5 Fishes (Fresh-water)
4.6 Fishes (Marine)
4.7 Other Marine Species
4.8 Invertebrates
4.9 Plants
4.10 Microbes

Appendix 4: **Species Listed as Known to be Invasive Elsewhere in the World**
5.1 Mammals
5.2 Birds
5.3 Reptiles
5.4 Amphibians
5.5 Fishes (Fresh-water)
5.6 Fishes (Marine)
5.7 Other Marine Species
5.8 Invertebrates
5.9 Plants
5.10 Microbes

Appendix 5: **Species Listed as Potentially Invasive Elsewhere in the World**
6.1 Mammals
6.2 Birds
6.3 Reptiles
6.4 Amphibians
6.5 Fishes (Fresh-water)
6.6 Fishes (Marine)
6.7 Other Marine Species
6.8 Invertebrates
6.9 Plants
6.10 Microbes

Appendix 1

Species Listed as Prohibited Alien Species

A list of species that are prohibited for introduction into South Africa is outlined below. These are species for which an assessment has been made that they have a high risk of causing significant negative impacts in the country, and that they are therefore prohibited from introduction. This list will be compiled over time by flagging taxa (species, genera or families) that are deemed to pose a high risk based on experience, both within South Africa, and within neighbouring countries and elsewhere in the world.

An applicant may, at his or her own cost, appeal against the listing of the species on the Prohibited Species list. This cost may be waived, should the appeal succeed.

The following species are listed as Prohibited Species:

1.1 Mammals

No	Species	Common Name
1	*Alcelaphus buselaphus*	Red hartebeest (all subspecies with the exception of *A.b. caama*)
2	*Bos frontalis*	Gaur
3	*Castor spp.*	Beaver (all species)
4	*Erinaceus europaeus*	European hedgehog
5	*Felis catus*	Feral cat (off-shore islands)
6	*Giraffa camelopardalis*	Giraffe (all subspecies excluding *capensis*)
7	*Herpestes javanicus*	Small Indian mongoose (off-shore islands)
8	*Hippotragus niger varianii*	Giant sable (except in breeding zoos)
9	*Kobus kafuensis*	Kafue lechwe
10	*Kobus kob*	Kob
11	*Kobus megaceros*	Nile lechwe
12	*Macaca fascicularis*	Crab-eating macaque
13	*Mustela erminea*	Short-tailed weasel / Stoat
14	*Oryctolagus cuniculus*	European rabbit (Islands)
15	*Oryx beisa*	Beisa oryx
16	*Rattus exulans*	Pacific/Polynesian rat (off-shore islands)
17	*Redunca redunca*	Bohor reedbuck
18	*Suncusmurinus*	Asian/Indian musk shrew
19	*Trichosurus vulpecula*	Brushtail possum
20	*Vulpes vulpes*	Red fox

1.2 Birds

No	Species	Common Name
1	*Acridotheres cristatellus*	Crested myna
2	*Acridotheres fuscus*	Jungle myna
3	*Alectoris rufa*	Red-legged partridge
4	*Callipepla californica*	California quail
5	*Callipepla gambelii*	Gambel's quail
6	*Callipepla squamata*	Scaled quail
7	*Carduelis carduelis*	European goldfinch
8	*Carduelis chloris*	European greenfinch
9	*Carduelis flammea*	Common redpoll
10	*Carpodacus mexicanus*	House finch
11	*Colinus cristatus*	Crested quail
12	*Colinus virginianus*	Northern bobwhite

13	*Columba palumbus*	Common wood-pigeon
14	*Corvus brachyrhynchos*	American crow
15	*Corvus frugilegus*	Rook
16	*Corvus monedula*	Eurasian jackdaw
17	*Eleutherodactylus coqui*	Puerto Rican coqui
18	*Emberiza citrinella*	Yellowhammer
19	*Foudia madagascariensis*	Madagascar red fody
20	*Francolinus pondicerianus*	Grey francolin
21	*Gracula religiosa*	Hill myna
22	*Icterus pectoralis*	Spot-breasted oriole
23	*Lonchura malacca*	Indian black-headed manikin
24	*Lonchura punctulata*	Scaly-breasted munia
25	*Molothrus ater*	Brown-headed cowbird
26	*Molothrus bonariensis*	Shiny cowbird
27	*Oreortyx pictus*	Mountain quail
28	*Oxyura jamaicensis*	Northern ruddy duck
29	*Oxyura leucocephala*	White-headed duck
30	*Passer hispaniolensis*	Spanish sparrow
31	*Passer montanus*	Eurasian tree sparrow
32	*Perdix perdix*	Grey partridge
33	*Phasianus colchicus*	Ring-necked pheasant
34	*Pycnonotus cafer*	Red-vented bulbul
35	*Pycnonotus jocosus*	Red-whiskered bulbul
36	*Sicalis flaveola*	Saffron finch
37	*Streptopelia picturata*	Madagascar turtle-dove
38	*Struthio camelus molybdophanus*	North African ostrich
39	*Turdus merula*	Blackbird
40	*Turdus philomelos*	Song thrush
41	*Zenaida asiatica*	White-winged dove

1.3 Reptiles

No	Species	Common Name
1	*Agama agama*	Common agama
2	*Anolis distichus*	Bark anole
3	*Anolis sagrei*	Brown anole
4	*Basiliscus vittatus*	Basilisk
5	*Boiga irregularis*	Brown tree snake
6	*Chelydra macroclemys*	Alligator snapper turtle
7	*Geochelone sulcata*	Spur thighed tortoise
8	*Hemidactylus frenatus*	House gecko
9	*Hemidactylus garnotti*	Indo-Pacific gecko
10	*Hemidactylus turcicus*	Mediterranean gecko
11	*Leiocephalus carinatus*	Northern curlytail lizard
12	*Macrochelys temminckii*	Snapper turtle
13	*Tarentola mauritanica*	Moorish wall gecko
14	*Trachemys scripta elegans*	Red-eared slider

1.4 Amphibians

No	Species	Common Name
1	*Ambystoma tigrinum*	Tiger salamander
2	*Bufo bufo*	European toad
3	*Bufo marinus*	Cane toad / Marine toad
4	*Eleutherodactylus planirostris*	Greenhouse frog
5	*Litoria caerulea*	Great green treefrog

6	*Notophthalmus viridenscens viridescens*	Red-spotted newt
7	*Osteopilus septentrionalis*	Cuban treefrog
8	*Rana catesbeiana*	American bull frog

1.5 Fishes (Fresh-water)

No	Species	Common name
1	*Clarias batrachus*	walking catfish

NB: The DoA list of fresh-water fishes, as agreed to with the industry, needs to be inserted here.

1.6 Fishes (Marine)

No	Species	Common name
1	SEE POINT 1 BELOW	

NB: The DoA list of marine fishes, as agreed to with the industry, needs to be inserted here.

1.7 Other Marine Species

No	Species	Common name
1	*Caulerpa taxifolia*	Caulerpa
2	*Undaria pinnatifida*	Asian kelp
3	*Eiochir sinensis*	Asian mitten crab
4	*Asteria anamurensis*	Pacific seastar

1.8 Invertebrates (Fresh-water)

No	*Species*	Common Name
1	*Cherax destructor*	Yabby

1.9 Invertebrates (Terrestrial)

1.9.1 Mollusca (snails and molluscs)

No	Species	Common name
1	*Achatina fulica* Bowdich, 1822	Giant African snail
2	*Euglandina rosea* (Férussac, 1821)	Rosy wolf snail

1.9.2 Nematoda (nematodes)

No	Species	Common name
1	*Aphelenchoides fragariae (*Ritzema Bos, 1890) Christie, 1932	Strawberry crimp disease nematode
2	*Belonolaimus longicaudatus* Rau, 1958	
3	*Bursaphelenchus xylophilus (*Steiner & Bührer, 1934) Nickel, 1970	Pine wilt or Pine wood nematode
4	*Globodera pallida (*Stone, 1973) Behrens, 1975	Pale cyst nematode, Potato cyst nematode
5	*Heterodera glycines* Ichinohe, 1952	Soybean cyst nematode
6	*Heterodera goettingiana* Liebscher, 1892	Pea cyst nematode
7	*Longidorus attenuatus* Hooper, 1961	Tomato docking disorder nematode
8	*Longidorus elongatus (*De Man, 1876) Micoletzky, 1922	Sugar beet docking disorder nematode
9	*Paratrichodorus nanus* (Allen, 1957) Siddiqi, 1974	
10	*Paratrichodorus pachydermus* (Seinhorst, 1954) Siddiqi, 1974	
11	*Paratrichodorus tunisiensis* (Siddiqi, 1963) Siddiqi, 1974	
12	*Paratylenchus bukowinensis* Micoletzky, 1922	
13	*Rhadinaphelenchus cocophilus* (Cobb, 1919) J.B. Goodey, 1960	Red ring disease nematode
14	*Scutellonema bradys (*Steiner & Lehew, 1933) Andrássy, 1958	Yam nematode
17	*Trichodorus primitivus* (De Man, 1880) Micoletzky, 1922	
18	*Trichodorus similes* Steinhorst, 1963	
19	*Trichodorus viruliferus* Hooper, 1963	

1.9.3 Insecta (insects)

	Species	Family
1.	*Aleurodicus destructor*	Aleyrodidae
2.	*Aleurodicus disperses*	Aleyrodidae
3.	*Amblypelta lutescens*	Coccidae
4.	*Anastrepha ludens*	Tephritidae
5.	*Anastrepha oblique*	Tephritidae
6.	*Anastrepha pseudoparallela*	Tephritidae
7.	*Anastrepha serpentine*	Tephritidae
8.	*Anastrepha striata*	Tephritidae
9.	*Anastrepha suspense*	Tephritidae
10.	*Archips argyrospilis*	Tortricidae
11.	*Argyrotaenia citrana*	Tortricidae
12.	*Bactrocera zonata*	Tephritidae
13.	*Bactrocera aquilonis*	Tephritidae
14.	*Bactrocera carambolae*	Tephritidae
15.	*Bactrocera caryeae*	Tephritidae
16.	*Bactrocera correta*	Tephritidae
17.	*Bactrocera curcubitae*	Tephritidae
18.	*Bactrocera dorsalis*	Tephritidae
19.	*Bactrocera facialis*	Tephritidae
20.	*Bactrocera frauenfeldi*	Tephritidae
21.	*Bactrocera jarvisi*	Tephritidae
22.	*Bactrocera kandiensis*	Tephritidae
23.	*Bactrocera kandiensisoccipitalis*	Tephritidae
24.	*Bactrocera kirki*	Tephritidae
25.	*Bactrocera latifrons*	Tephritidae
26.	*Bactrocera melanota*	Tephritidae
27.	*Bactrocera musae*	Tephritidae
28.	*Bactrocera neohumeralis*	Tephritidae
29.	*Bactrocera occipitalis*	Tephritidae
30.	*Bactrocera papayae*	Tephritidae
31.	*Bactrocera passiflorae*	Tephritidae
32.	*Bactrocera philippensis*	Tephritidae
33.	*Bactrocera psidi*	Tephritidae
34.	*Bactrocera pyrifoliae*	Tephritidae
35.	*Bactrocera tryoni*	Tephritidae
36.	*Bactrocera xanthodes*	Tephritidae
37.	*Castnia licoides*	Castniidae
38.	*Castnia penelope*	Castniidae
39.	*Ceroplastes floridensis*	Coccidae
40.	*Chloropulvinaria polygnota*	Coccidae
41.	*Choristoneura rosaceana*	Tortricidae
42.	*Chrysodeixis eriosoma*	Noctuidae
43.	*Cnephasia jactatana*	Tortricidae
44.	*Colaspis hypochlora*	Chrysomelidae
45.	*Conogethes punctiferalis*	Pyralidae
46.	*Conopomorpha litchiella*	Gracillariidae
47.	*Cryptophlebia illepida*	Tortricidae
48.	*Cryptophlebia onbrodelta*	Tortricidae
49.	*Ctenopseustis obliquana*	Tortricidae
50.	*Dudua aprobola*	Tortricidae
51.	*Dysmicoccus neobrevipes*	Pseudococcidae
52.	*Epiphyas postvittana*	Tortricidae
53.	*Erionota thrax*	Hesperiidae
54.	*Holothrips ananasi*	Thripidae
55.	*Lachnopus sp. near campechianus*	Curculionidae
56.	*Lobesia aeolopa*	Tortricidae
57.	*Maconellicoccus hirsutus*	Pseudococcidae
58.	*Metamasius callizona*	Curculionidae
59.	*Nacoleia octasema*	Pyralidae
60.	*Neodecadarchis flavistriata*	Tineidae
61.	*Odoiporus longicollis*	Curculionidae
62.	*Phenacoccus madeirensis*	Pseudococcidae
63.	*Planococcoides njalensis*	Pseudococcidae
64.	*Planococcus litchi*	Pseudococcidae
65.	*Planococcus minor*	Pseudococcidae
66.	*Planotortix excessana*	Tortricidae
67.	*Platynota stultana*	Tortricidae
68.	*Proeulia auraria*	Tortricidae
69.	*Proeulia chrysopteris*	Tortricidae
70.	*Pseudococcus calceolariae*	Pseudococcidae
71.	*Pseudococcus comstocki*	Pseudococcidae
72.	*Pseudococcus cryptus*	Pseudococcidae
73.	*Pseudococcus elisae*	Pseudococcidae

74.	*Pseudococcus jackbeardsleyi*	Pseudococcidae
75.	*Rastrococcus iceryoides*	Pseudococcidae
76.	*Rastrococcus invadens*	Pseudococcidae
77.	*Rastrococcus mangiferae*	Pseudococcidae
78.	*Rastrococcus spinosus*	Pseudococcidae
79.	*Rhipiphorothrips cruentatus*	Thripidae
80.	*Spodoptora litura*	Noctuidae
81.	*Sternochetus frigidus*	Curculionidae
82.	*Thecla basilides*	Lycaenidae
83.	*Thecla legota*	Lycaenidae
84.	*Thrips hawaiiensis*	Thripidae
85.	*Thrips palmi*	Thripidae
86.	*Tmolus echion*	Lycaenidae
87.	*Toxotrypana curvicauda*	Aleyrodidae
88.	*Unaspis citri*	Diaspididae
89.	*Vinsonia stellifera*	Coccidae

1.9.4 Acari (Mites)

	Species	Family
1	*Tuckerella pavoniformis*	Tuckerellidae
2	*Calacarus brionesae*	Eriophyidae
3	*Cisaberoptus kenyae*	Eriophyidae
4	*Tetranychus desertorum*	Tetranychidae
5	*Tetranychus piercei*	Tetranychidae
6	*Tetranychus mexicanus*	Tetranychidae
7	*Oligonychus biharensis*	Tetranychidae
8	*Oligonychus biharensis*	Tetranychidae
9	*Oligonychus punicae*	Tetranychidae
10	*Oligonychus yothersi*	Tetranychidae

1.9.5 Arachnida (Spiders)

	Species	Family and/or Common name
1	*Acanthoscurria geniculata*	Theraphosidae Brazilian white knee tarantula
2	*Avicularia aviculatria*	Theraphosidae Pink toe tarantla
3	*Brachypelma albopilosum*	Theraphosidae Curly hair tarantula
4	*Brachypelma smithi*	Theraphosidae Mexican red knee tarantula
5	*Brachypelma vegans*	Theraphosidae Red rump tarantula
6	*Grammostola rosae*	Theraphosidae Chilean rose tarantula
7	*Haplopelma lividum*	Theraphosidae Cobalt blue tarantula
8	*Lasiodora parahybana*	Theraphosidae Brazilan salmon pink tarantula
9	*Poecilotheria regalis*	Theraphosidae Indian ornamental tarantula
10	*Psalmopoeus cambridgei*	Theraphosidae Trinidad chevron tarantula
11	*Theraphosa blondi*	Theraphosidae Goliath bird eating tarantula

1.10 Plants

No	Species	Common Name
1	*Acaena pallida* (Kirk) Allan	Pale biddy-biddy
2	*Achnatherum caudatum* (Trin.) S.W.L.Jacobs & J.Everett	Spear grass
3	*Achnatherum brachychaetum* (Godr.) Barkworth (= *Nassella brachychaeta* (Godr.) Barkworth, *Stipa brachychaeta* Godr.)	Puna grass
4	*Aegilops* spp.	Goat grass
5	*Aegilops cylindrica* Host (= *Cylindropyrum cylindricum* (Host) Á.Löve, *Triticum cylindricum* (Host) Ces.	Jointed goat grass
6	*Aegilops geniculata* Roth (= *A. ovata* auct., *Triticum ovatum* auct.)	Ovate goat grass
7	*Aegilops triuncialis* L. (= *A. squarrosa* L., *Triticum triunciale* (L.) Raspail	Barb goat grass
8	*Aeginetia* spp.	Aeginetia
9	*Aeschynomene rudis* Benth.	Rough joint-vetch
10	*Allium paniculatum* L.	Panicled onion
11	*Allium vineale* L. (= *A. kochii* Lange)	Wild garlic
12	*Alternanthera philoxeroides* (Mart.) Griseb.	Alligator weed
13	*Ambrosia trifida* L.	Giant ragweed
14	*Andropogon bicornis* L.	West Indian foxtail grass
15	*Andropogon virginicus* L.	Broom-sedge
16	*Annona glabra* L.	Pond apple
17	*Artemisia verlotiorum* Lamotte	Mugwort
18	*Arundinaria* spp.	Arundinaria reeds/bamboos
19	*Baccharis halimifolia* L.	Groundsel bush
20	*Berberis glaucocarpa* Stapf	Barberry
21	*Bifora testiculata* (L.) Spreng. (*Coriandrum testiculatum* L.)	Bifora
22	*Cabomba caroliniana* A.Gray	Cabomba, Carolina fanwort

23	*Cabomba* spp.	Cabomba
24	*Callistachys lanceolata* Vent. (= *Oxylobium lanceolatum* (Vent.) Druce	Oxylobium
25	*Calluna vulgaris* (L.) Hull	Heather
26	*Calotis lappulacea* Benth.	Bur-daisy
27	*Lepidium draba* L. subsp. *chalepense* (L.) Thell. (= *Cardaria chalepensis* (L.) Hand.-Mazz	Lens podded hoary cress
28	*Lepidium appelianum* Al-Shehbaz (= *Cardaria pubescens* (C.A.Mey.) Jarm.	Globe-pod hoary cress
29	*Carduus acanthoides* L.	Plumeless thistle
30	*Carduus nutans* L.	Musk thistle
31	*Carduus pycnocephalus* L.	Italian thistle
32	*Carex buchananii* Bergg.	New Zealand sedge
33	*Carex comans* Bergg. (= *C. albula* Allan)	New Zealand sedge
34	*Carex flagellifera* Colenso	New Zealand sedge
35	*Carex longebrachiata* Boeckeler	Australian sedge
36	*Carex testacea* Sol. ex Boott	New Zealand sedge
37	*Carthamus leucocaulos* Sm.	White-stem distaff thistle, Saffron thistle
38	*Carthamus oxyacanthus* M.Bieb. (= *C. flavescens* Willd.)	Wild safflower
39	*Cassinia arcuata* R.Br.	Chinese shrub, Drooping cassinia
40	*Caulerpa taxifolia* (Vahl) C.Agardh	Notched caulerpa, Feather caulerpa
41	*Celastrus orbiculatus* Thunb. (= *C. articulatus* Thunb.)	Climbing spindleberry, Oriental bittersweet
42	*Cenchrus echinatus* L.	Southern sandbur grass, Mossman River grass
43	*Cenchrus longispinus* (Hack.) Fernald	Mat sandbur, Spiny bur grass
44	*Centaurea stoebe* L. subsp. *micranthos* (Gugler) Hayek (= *Centaurea biebersteinii* auct., *C. maculosa* auct.)	Spotted knapweed
45	*Centaurea diffusa* Lam.	Diffuse knapweed
46	*Centaurea iberica* Trevir. ex Spreng.	Iberian star thistle
47	*Centaurea sulphurea* Willd.	Sicilian star thistle
48	*Centaurea virgata* Lam. subsp. *squarrosa* (Boiss.) Gugler (= *C. squarrosa* Willd., *C. virgata* auct.)	Squarrose knapweed
49	*Cereus hildmannianus* K.Schum. (= *C. peruvianus* auct. pl. [= *C. hildmannianus* subsp. *hildmannianus*], *C. uruguayanus* C.Ritter ex R.Kiesling [= *C. hildmannianus* subsp. *uruguayanus*]	Queen-of-the-night, Peruvian apple, Hedge cactus, Spiny tree cactus
50	*Chorispora tenella* (Pall.) DC.	Purple mustard
51	*Chrysopogon aciculatus* (Retz.) Trin. (= *Andropogon acicularis* Retz. ex Roem. & Schult., *A. aciculatus* Retz., *Rhaphis acicularis* (Retz. ex Roem. & Schult.) Desv., *R. aciculata* (Retz.) Honda	Pilipiliula
52	*Cirsium japonicum* Fisch. ex DC.	Japanese thistle
53	*Cirsium ochrocentrum* A.Gray	Yellow-spine thistle
54	*Cirsium undulatum* (Nutt.) Spreng.	Wavy-leaf thistle
55	*Clematis vitalba* L.	Old man's beard
56	*Clidemia hirta* (L.) D.Don (= *Melastoma hirtum* L.)	Koster's curse
57	*Coccinia grandis* (L.) Voigt (= *C. cordifolia* auct., *C. indica* Wight & Arn.	Ivy gourd
58	*Cortaderia richardii* (Endl.) Zotov	New Zealand pampas grass
59	*Crassula helmsii* (Kirk) Cockayne	Swamp stonecrop
60	*Crataegus sinaica* Boiss.	Azzarola
61	*Crupina vulgaris* Cass.	Common crupina, Bearded creeper
62	*Cupaniopsis anacardioides* (A.Rich.) Radlk. (= *Cupania anacardioides* A.Rich.)	Carrotwood
63	*Cuscuta indecora* Choisy (= *C. jepsonii* Yunck.)	Large-seeded dodder
64	*Cuscuta reflexa* Roxb.	Giant dodder, Indian dodder
65	*Cymbopogon refractus* (R.Br.) A.Camus (= *Andropogon refractus* R.Br.)	Barbwire grass
66	*Datura leichhardtii* F.Muell. ex Benth. (= *D. pruinosa* Greenm.)	Leichhardt's thorn apple
67	*Datura wrightii* Regel (= *D. meteloides* auct.)	Hairy thorn apple
68	*Diplotaxis tenuifolia* (L.) DC. (= *Sisymbrium tenuifolium* L.)	Sand rocket
69	*Dipsacus fullonum* L. (= *D. sylvestris* Huds.)	Wild teasel
70	*Dioscorea alata* L. (= *D. rubella* Roxb.)	White yam
71	*Drymaria arenarioides* Humb. & Bonpl. ex Schult.	Alfombrilla, Lightningweed
72	*Echium italicum* L.	Italian bugloss
73	*Eichhornia azurea* (Sw.) Kunth	Anchored water hyacinth
74	*Elaeagnus* X *reflexa* C.Morren & Decne. (= *E. pungens* Thunb. var. *reflexa* (C. Morren & Decne.) C.K.Schneid.	Elaeagnus
75	*Elephantopus mollis* Kunth (= *E. tomentosus* auct. nonn.)	Elephantopus, Elephant's foot
76	*Emex spinosa* (L.) Campd. (= *Rumex spinosus* L.)	Spiny emex, Devil's thorn, Lesser jack
77	*Equisetum arvense* L.	Field horsetail, Common horsetail
78	*Erica lusitanica* Rudolphi	Spanish heath, Portuguese heath
79	*Euphorbia esula* L. (= *E.* X *pseudovirgata* (Schur) Soó, *E. tommasiniana* Bertol., *E. virgata* Waldst. & Kit.)	Leafy spurge
80	*Euphorbia oblongata* Griseb.	Oblong spurge
81	*Euphorbia terracina* L.	Geraldton carnation
82	*Fallopia japonica* (Houtt.) Ronse Decr. (*Polygonum cuspidatum* Siebold & Zucc., *Polygonum reynoutria* Makino, *Reynoutria japonica*	Japanese knotweed

	Houtt.)	
83	*Fallopia* X *bohemica* (Chrtek. & Chrtková) J.P.Bailey	Japanese knotweed hybrid
84	*Galium tricornutum* Dandy (= *G. tricorne* Stokes pro parte)	Three-horned bedstraw, Corn-cleavers
85	*Gaura coccinea* Pursh (= *G.odorata* Sessé ex Lag.)	Scarlet gaura
86	*Gaura drummondii* (Spach) Torr. & A.Gray (= *G. odorata* auct.)	Drummond's gaura
87	*Gmelina asiatica* L.	Badhara bush
88	*Gunnera tinctoria* (Molina) Mirb. (= *G. chilensis* Lam., *Panke tinctoria* Molina)	Chilean rhubarb
89	*Gymnocoronis spilanthoides* DC.	Senegal tea plant, Temple plant
90	*Gypsophila paniculata* L.	Baby's breath
91	*Halimodendron halodendron* (Pall.) Voss (= *Caragana argentea* Lam., *Halimodendron argenteum* (Lam.) DC., *Robinia halodendron* Pall.)	Russian salt tree
92	*Halogeton glomeratus* (M.Bieb.) C.A.Mey. (= *Anabasis glomerata* M.Bieb.)	Halogeton
93	*Harungana madagascariensis* Lam. ex Poir. (= *Haronga madagascariensis* (Lam. ex Poir.) Choisy	Harungana, Haronga, Dragon's-blood-tree
94	*Helianthus ciliaris* DC.	Blueweed, Texas blueweed
95	*Hieracium aurantiacum* L. (= *Pilosella aurantiaca* (L.) F.W.Schultz & Sch. Bip.)	Orange hawkweed
96	*Hieracium pilosella* L. (= *Pilosella officinarum* F.W. Schultz & Sch. Bip.)	Mouse-ear hawkweed
97	*Hieracium praealtum* Gochn.	King devil
98	*Hydrilla verticillata* (L.f.) Royle	Hydrilla
99	*Hydrocharis morsus-ranae* L.	Frog's-bit
100	*Hydrodictyon reticulatum* Lagerh.	Water net
101	*Hygrophila costata* Nees et al. (= *H. brasiliensis* (Spreng.) Lindau*, H. guianensis* Nees*, Ruellia brasiliensis* Spreng.	Hygrophila
102	*Hygrophila polysperma* (Roxb.) T.Anderson (= *Justicia polysperma* Roxb.)	Miramar weed, Hygrophila
103	*Hymenachne amplexicaulis* (Rudge) Nees	Olive hymenachne, West Indian marsh grass
104	*Hypericum androsaemum* L.	Tutsan
105	*Hypericum* X *inodorum* Mill. (= *H. elatum* Aiton)	Tall St John's wort, Tall tutsan
106	*Hypericum triquetrifolium* Turra (= *H. crispum* L.)	Tangled hypericum
107	*Hyptis capitata* Jacq.	Knobweed
108	*Hyptis pectinata* (L.) Poit. (= *Nepeta pectinata* L.)	Comb hyptis
109	*Hyptis suaveolens* (L.) Poit. (= *Ballota suaveolens* L.)	Wild spikenard, Hyptis
110	*Imperata brasiliensis* Trin.	Brazilian satin-tail
111	*Imperata brevifolia* Vasey (= *Imperata hookeri* (Rupr. ex Andersson) Hack.	Satin-tail
112	*Ipomoea triloba* L.	Little-bell, Aiea morning-glory
113	*Iris douglasiana* Herb.	Douglas iris
114	*Iris missouriensis* Nutt.	Western blue flag, Rocky Mountain iris
115	*Ischaemum rugosum* Salisb.	Murain-grass
116	*Iva axillaris* Pursh	Poverty weed
117	*Iva axillaris* Pursh subsp. *robustior*	Poverty weed
118	*Juncus acutus* L. subsp. *acutus*	Spiny rush
119	*Lagascea mollis* Cav.	Acuate
120	*Lepidium latifolium* L.	Perennial pepperweed, Perennial peppercress
121	*Limnobium spongia* (Bosc) Steud. (= *Hydrocharis spongia* Bosc)	American spongeplant
122	*Limnocharis flava* (L.) Buchenau (= *Alisma flavum* L., *Limnocharis emarginata* Bonpl.)	Sawah flowering rush, Yellow burrhead
123	*Limnophila indica* (L.) Druce (= *Hottonia indica* L.)	Ambulia
124	*Limnophila sessiliflora* (Vahl) Blume (= *Hottonia sessiliflora* Vahl	Ambulia
125	*Linaria dalmatica* (L.) Mill. (= *Antirrhinum dalmaticum* L., *Linaria genistifolia* subsp. *dalmatica* (L.) Maire & Petitm.)	Dalmatian toadflax, Broadleaf toadflax
126	*Ludwigia peploides* (Kunth) P.H.Raven	Primrose willow, Creeping water-primrose
127	*Malachra alceifolia* Jacq.	Malachra
128	*Malvella leprosa* (Ortega) Krapov. (= *Sida hederacea* (Douglas ex Hook.) Torr. ex A.Gray*, Sida leprosa* var. *hederacea* (Douglas ex Hook.) K.Schum.	Alkali mallow, Alkali sida
129	*Marsilea mutica* Mett.	Nardoo, Australian water-clover
130	*Martynia annua* L. (= *M. diandra* Gloxin)	Devil's claw
131	*Medinilla venosa* (Blume) Blume (= *Melastoma venosum* Blume	
132	*Melastoma malabathricum* L. (= *Melastoma normale* D.Don)	Indian-rhododendron
133	*Melastoma* spp.	Melastoma
134	*Menyanthes trifoliata* L.	Bog bean
135	*Miconia* spp.	Miconia
136	*Mikania cordata* (Burm.f.) B.L.Rob.	Mile-a-minute
137	*Mikania micrantha* Kunth	Mile-a-minute, Climbing hempweed
138	*Mikania scandens* (L.) Willd. (= *Eupatorium scandens* L., *Willoughbya scandens* (L.) Kuntze	Climbing hempweed
139	*Mimosa diplotricha* C.Wright (= *M. invisa* Mart.)	Giant sensitive-plant
140	*Miscanthus floridulus* (Labill.) Warb. ex K.Schum. & Lauterb. (= *M. japonicus* Andersson, *Saccharum floridulum* Labill.	Giant Chinese silver grass, Japanese silver grass

141	*Monochoria hastata* (L.) Solms	Arrow-leaf monochoria, Hastate-leaf-pondweed
142	*Monochoria vaginalis* (Burm.f.) C.Presl. ex Kunth (= *M. vaginalis var. pauciflora* Merr.*, Pontederia vaginalis* Burm.f.)	Oval-leaf monochoria, Oval-leaf-pondweed, Pickerel-weed
143	*Muhlenbergia schreberi* J.F.Gmel. (= *M. diffusa* Willd.)	Nimblewill
144	*Myagrum perfoliatum* L.	Muskweed
145	*Najas guadalupensis* (Spreng.) Magnus	Southern naiad
146	*Nassella charruana* (Arechav.) Barkworth (= *Stipa charruana* Arechav.	Lobed needlegrass
147	*Nassella hyalina* (Nees) Barkworth (= *Stipa hyalina* Nees)	Cane needlegrass
148	*Nassella leucotricha* (Trin. & Rupr.) R.W.Pohl (= *Stipa leucotricha* Trin. & Rupr.	Texas needlegrass
149	*Nechamandra alternifolia* (Roxb.) Thwaites (= *Vallisneria alternifolia* Roxb.)	
150	*Neyraudia reynaudiana* (Kunth) Keng ex Hitchc. (= *Arundo reynaudiana* Kunth)	Burma reed
151	*Nuphar lutea* (L.) Sm. (= *N. minor* Dumort., *N. sericea* Láng, *N. spathulifera* Rchb., *N. tenella* Rchb., *Nymphaea lutea* L., *N. umbilicalis* Salisb., *Nymphozanthus luteus* (L.) Fernald, *N. sericeus* (Láng) Fernald, *N. vulgaris* Rich.)	Yellow water-lily
152	*Nymphoides geminata* (R.Br.) Kuntze (= *Villarsia geminata* R.Br.)	Entire marshwort
153	*Nymphoides peltata* (S.G.Gmel.) Kuntze (= *Limnanthemum peltatum* S.G.Gmel.)	Gringed waterlily, Yellow floating-heart
154	*Oenanthe pimpinelloides* L.	Water dropwort, Corky-fruit water-dropwort
155	*Ononis alopecuroides* L.	Foxtail restharrow
156	*Onopordum acaulon* L.	Stemless thistle
157	*Onopordum illyricum* L.	Illyrian thistle
158	*Onopordum tauricum* Willd.	Taurian thistle, Taurean thistle
159	*Orobanche cooperi* (A.Gray) A.Heller (= *O. ludoviciana* Nutt. var. *cooperi* (A.Gray) Beck	Cooper's broomrape
160	*Oryza rufipogon* Griff. (= *O. fatua* J. König ex Trin., nom. nud.*, O. sativa* L. var. *fatua* Prain)	Red rice, Perennial wild red rice
161	*Ottelia alismoides* (L.) Pers.	Duck-lettuce, Water-plantain ottelia
162	*Oxyspora paniculata* (D.Don) DC. (= *Arthrostemma paniculatum* D.Don)	
163	*Paederia cruddasiana* Prain	Sewer vine
164	*Paederia foetida* L. (= *P. magnifica* Noronha, nom. nud.*, P. scandens* (Lour.) Merr.*, P. tomentosa* Blume*, Gentiana scandens* Lour.)	Skunk vine
165	*Panicum antidotale* Retz. (= *P. miliare* Lam.*, P. proliferum* Lam.)	Blue panic grass
166	*Parietaria judaica* L. (= *P. diffusa* Mert. & W.D.J. Koch)	Wall pellitory
167	*Passiflora bicornis* Mill. (= *P. pulchella* Kunth)	Wingleaf passionfruit
168	*Pennisetum alopecuroides* (L.) Spreng. (= *Alopecurus hordeiformis* L.*, Panicum alopecuroides* L.*, Pennisteum compressum* R.Br.*, P. hordeiforme* (Thunb.) Spreng.*, P. japonicum* Trin. ex Spreng.	Chinese pennisetum, Swamp foxtail grass
169	*Pennisetum pedicellatum* Trin.	Kyasuwa-grass
170	*Pennisetum polystachion* (L.) Schult.	Mission grass, Thin Napier grass
171	*Persicaria perfoliata* (L.) H.Gross (= *Polygonum perfoliatum* L.)	Devil's tail tearthumb, Mile-a-minute-vine, Mile-a-minute-weed
172	*Persicaria wallichii* Greuter & Burdet (= *P. polystachya* (Wall. ex Meisn.) H.Gross, *Polygonum polystachyum* Wall. ex Meisn.*, Rubrivena polystachya* (Wall. ex Meisn.) M.Král)	Himalayan knotweed
173	*Physalis longifolia* Nutt. (= *P. subglabrata* Mack. & Bush, *P. virginiana* Mill. var. *sonorae* (Torr.) Waterf.	Long-leaf ground-cherry
174	*Picnomon acarna* (L.) Cass.	Soldier thistle
175	*Piper aduncum* L. (= *P. angustifolium* Ruiz & Pav.*, P. celtidifolium* Kunth*, P. elongatum* Vahl)	Spiked pepper, Piper
176	*Potamogeton perfoliatus* L.	Clasped pondweed
177	*Pontederia rotundifolia* L.f. (= *Reussia rotundifolia* (L.f.) A.Cast.	Tropical pickerel-weed
178	*Prosopis alpataco* Phil.	Mesquite
179	*Prosopis argentina* Burkhart	Mesquite
180	*Prosopis bukartii* Muñoz	Mesquite
181	*Prosopis caldenia* Burkart	Mesquite
182	*Prosopis calingastana* Burkart	Cusqui, Mesquite
183	*Prosopis campestris* Griseb.	Mesquite
184	*Prosopis castellanosii* Burkart	Mesquite
185	*Prosopis denudans* Benth. (= *P. patagonica* Speg.)	Mesquite
186	*Prosopis elata* (Burkart) Burkart (= *P. campestris* Griseb. var. *elata* Burkart)	Mesquite
187	*Prosopis farcta* (Banks & Sol.) J.F.Macbr. (= *Mimosa farcta* Banks & Sol.)*, M. stephaniana* M.Bieb.*, P. stephaniana* (M.Bieb.) Kunth ex Spreng.)	Syrian mesquite
188	*Prosopis ferox* Griseb.	Mesquite
189	*Prosopis fiebrigii* Harms	Mesquite
190	*Prosopis hassleri* Harms	Mesquite
191	*Prosopis humilis* Gillies ex Hook. & Arn.	Mesquite
192	*Prosopis kuntzei* Harms	Mesquite
193	*Prosopis palmeri* S.Watson	Mesquite

194	*Prosopis reptans* Benth. (= *P. cinerascens* (A.Gray) Benth., *Strombocarpa cinerascens* A.Gray)	Mesquite
195	*Prosopis rojasiana* Burkart	Mesquite
196	*Prosopis ruizlealii* Burkart	Mesquite
197	*Prosopis ruscifolia* Griseb.	Mesquite
198	*Prosopis sericantha* Gillies ex Hook. & Arn.	Mesquite
199	*Prosopis strombulifera* (Lam.) Benth. (= *Acacia strombulifera* (Lam.) Willd., *Mimosa strombulifera* Lam.)	Argentine screwbean, Creeping mesquite
200	*Prosopis torquata* (Cav. ex Lag.) DC. (= *Acacia torquata* Cav. ex Lag.)	Mesquite
201	*Pueraria phaseoloides* (Roxb.) Benth. (= *Dolichos phaseoloides* Roxb., *Neustanthus javanicus* Benth., *Pueraria javanica* (Benth.) Benth.)	Tropical kudzu
202	*Ranunculus acris* L. (= *R. friesianus* Jord.)	Giant buttercup
203	*Ranunculus sceleratus* L.	Celery-leaf buttercup
204	*Reseda phyteuma* L.	Rampion mignonette
205	*Rorippa austriaca* (Crantz) Besser (= *Nasturtium austriacum* Crantz)	Austrian field cress
206	*Rorippa sylvestris* (L.) Besser	Creeping yellow field cress
207	*Rubus anglocandicans* A.Newton	Blackberry
208	*Rubus argutus* Link	Prickly Florida blackberry
209	*Rubus ellipticus* Sm. (= *R. ellipticus* var. *obcordatus* (Franch.) Focke , *R. flavus* Buch.-Ham. ex D.Don, *R. gowreephul* Roxb.)	Yellow Himalayan raspberry
210	*Rubus moluccanus* L. (= *R. hillii* F.Muell.)	Wild blackberry, Wild raspberry
211	*Rubus sieboldii* Blume	Molucca raspberry
212	*Saccharum spontaneum* L. (= *S. arenicola* Ohwi, *S. biflorum* Forssk.)	Wild sugarcane
213	*Sagittaria montevidensis* Cham. & Schltdl. (= *Lophotocarpus spongiosus* (Engelm.) J.G.Sm., *S. calycina* Engelm.)	Giant arrowhead
214	*Sagittaria platyphylla* (Engelm.) J.G.Sm. (= *S. graminea* Michx. var. *platyphylla* Engelm.)	Sagittaria, Delta arrowhead
215	*Salsola collina* Pall.	Spineless Russian thistle, Tumbleweed
216	*Salsola paulsenii* Litv.	Barbwire Russian thistle
217	*Salsola vermiculata* L.	Wormleaf salsola, Wormleaf saltwort
218	*Salvia aethiopis* L.	Mediterranean sage
219	*Salvia virgata* Jacq. (= *S. campestris* M.Bieb.)	Meadow sage
220	*Salvinia auriculata* Aubl. (= *S. rotundifolia* Willd.)	Salvinia, giant salvinia
221	*Salvinia biloba* Raddi	Giant salvinia
222	*Salvinia herzogii* de la Sota	Giant salvinia
223	*Sclerolaena birchii* (F.Muell.) Domin (= *Anisacantha birchii* F.Muell.)	Galvanised burr
224	*Scolymus hispanicus* L.	Golden thistle
225	*Scolymus maculatus* L.	Spotted golden thistle
226	*Senecio jacobaea* L.	Tansy ragwort, St james' ragwort
227	*Senecio squalidus* L.	Oxford ragwort
228	*Senna tora* (L.) Roxb. (= *Cassia tora* L.)	Java bean, Sicklepod senna
229	*Setaria faberi* R.A.W.Herrm.	Chinese foxtail, Giant foxtail
230	*Setaria palmifolia* (J.König) Stapf (= *Panicum palmifolium* Willd. ex Poir., *Chaetochloa palmifolia* Hitchc. & Chase)	Palm grass
231	*Solanum carolinense* L.	Horse nettle, Carolina horse nettle
232	*Solanum dimidiatum* Raf. (= *S. torreyi* A.Gray)	Torrey's nightshade
233	*Solanum lanceolatum* Cav.	Lance-leaf nightshade
234	*Solanum marginatum* L.f.	White-margined nightshade, White-edged nightshade
235	*Solanum robustum* H.L.Wendl. (= *S. alatum* Seem. & J. C. Schmidt)	Silver-leaf nightshade
236	*Solanum tampicense* Dunal	Wetland nightshade
237	*Sonchus arvensis* L.	Perennial sow thistle
238	*Sorghum hybrid* cv. *Silk*	Silk forage sorghum
239	*Sorghum X almum* Parodi (= *S. bicolor* (L.) Moench X *S. halepense* (L.) Pers.	Columbus grass
240	*Sparganium erectum* L. (= *S. chlorocarpum* Rydb., *S. polyedrum* (Asch. & Graebn.) Juz., *S. ramosum* Huds.)	Exotic bur-reed, Bur reed
241	*Spermacoce alata* Aubl. (= *Borreria alata* (Aubl.) DC.)	Borreria, Buttonweed
242	*Sphaerophysa salsula* (Pall.) DC. (= *Colutea caspica* M.Bieb., *Phaca salsula* Pall., *Swainsona salsula* (Pall.) Taub.)	Austrian peaweed
243	*Sporobolus indicus* (L.) R.Br. var. *major* (Büse) Baaijens (= *Agrostis fertilis* Steud., *S. diandrus* (Retz) P.Beauv. var. *major* Büse, *S. elongatus* R.Br. var. *purpureo-suffusus* Ohwi, *S. fertilis* (Steud.) Clayton, *S. indicus* var. *fertilis* (Steud.) Jovet & Guédès, *S. indicus* var. *purpureo-suffusus* (Ohwi) T.Koyama)	Giant Parramatta grass
244	*Stratiotes aloides* L.	Water-aloe, Soldier plant, Water-soldier
245	*Symphytum asperum* Lepech. (= *S. asperrimum* Sims)	Prickly comfrey, Rough comfrey
246	*Taeniatherum caput-medusae* (L.) Nevski (= *Cuviera caput-medusae* (L.) Koeler var. *aspera* Simonk., *Elymus caput-medusae* L., *E. crinitus* Schreb., *Hordeum crinitum* (Schreb.) Desf., *T. asperum* (Simonk.) Nevski, *T. crinitum* (Schreb.) Nevski	Medusa-head, Medusa's-head
247	*Themeda quadrivalvis* (L.) Kuntze (= *Andropogon quadrivalvis* L., *Anthristia ciliata* L.f., *Themeda ciliata* (L.f.) Hack.)	Grader grass, Habana grass

248	*Themeda villosa* (Poir.) A.Camus (= *Anthistiria villosa* Poir., *Themeda gigantea* (Cav.) Hack. subsp. *villosa* (Poir.) Hack.)	Lyon's grass
249	*Thunbergia annua* Hochst.	Thunbergia
250	*Thunbergia fragrans* Roxb. (= *T. volubilis* Pers., *Flemingia grandiflora* Roxb. ex Rottler)	Fragrant thunbergia, White thunbergia
251	*Tribulus cistoides* L. (= *T. terrestris* L. var. *cistoides* (L.) Oliv.)	Caltrop
252	*Triumfetta semitriloba* Jacq.	Sacramento bur
253	*Tropaeolum speciosum* Poepp. & Endl.	Chilean flame creeper, Flame nasturtium
254	*Vallisneria gigantea* Graebn.	Eelgrass
255	*Verbascum thapsus* L.	Mullein, Great mullein
256	*Viscum album* L.	European mistletoe
257	*Zizania latifolia* (Griseb.) Turcz. ex Stapf (= *Z. caduciflora* Hand.-Mazz., *Hydropyrum latifolium* Griseb.)	Manchurian wild rice
258	*Zygophyllum fabago* L. (= *Z. fabago* L. var. *brachycarpum* Boiss.)	Syrian bean-caper

1.11 Microbes

No	Species	Common Name
	**Fungi**	
1	*Alternaria triticina*	
2	*Clavibacter michiganensis*	
3	*Corticium koleroga*	Thread blight
4	*Corticium stevensii*	Thread blight
5	*Erwinia amylovora*	
6	*Fusarium oxysporum* f. sp. *albedines*	Phoenix wilt
7	*Guignardia bidwellii*	
8	*Liberobacter asiaticum*	
9	*Monilinia fructicola*	
10	*Nectria galligena*	
11	*Pantoea stewartii*	
12	*Phakopsora euvitis*	
13	*Phoma tracheiphila*	
14	*Phymatotrichopsis omnivora*	Root rot fungus
15	*Phytophthora fragariae* var. *fragariae*	Red stele rot of strawberries
16	*Phytophthora fragariae* var. *rubi*	Red stele root rot of Rubus
17	*Phytophthora ramorum*	Sudden Oak death
18	*Phytophthora palmivora*	Phoenix palm disease
19	*Xanthomonas axonopodis pv. citri*	
20	*Xylelia fastidiosa*	
	**Virusses and phytoplasmas**	
1	Apple proliferation phytoplasma	
2	Banana bunchy top nanavirus	
3	Cadang cadang viroid	
4	Cotton leaf curl bigeminivirus	
5	Maize rough dwarf fijivirus	
6	Tomato bushy stunt tombusvirus	
7	Tomato ringspot nepovirus	

Appendix 2

Species Listed as Permitted Alien Species

The list of species that are permitted for introduction into South Africa is based on species that have already been introduced into South Africa and have been adjudged not to be invasive. This list will be compiled over time, to facilitate the ease of introduction of species with an acceptably low risk of becoming invasive.

Permitted Species are those that have been found or ascertained to have a negligible or no risk of invasion as a species, and which are therefore allowed to be brought into the country without having to undergo a Species Risk Assessment. The authorities will retain the right to require new genotypes (e.g. cultivars, varieties, genotypes) to undergo a Species Risk Assessment.

The approval to import a species into the country without any permit conditions may result in the species being put onto the list of Permitted Species.

The status as a Permitted Species does not absolve an importer from the requirements of a Vector Risk Assessment.

It will be possible to appeal against the presence of a species in the Permitted Species list, at the cost of the person or group lodging the appeal. This cost may be waived should the appeal succeed.

The following species are listed as Permitted Species:

2.1 Mammals

No	Species	Common name - English
1	*Bubalus bubalis*	Water buffalo
2	*Bos taurus*	Cattle
3	*Bos indicus*	Cattle
4	*Camelus dromedarius*	Camels
5	*Canis lupus*	Wolves
6	*Equus asinus*	Donkey
7	*Lama glama*	Lama
8	*Ovis aries*	Sheep

NB: The DoA's list of all legal imports of mammals for agricultural purposes, that are not listed as invasive, to be added here.

2.2 Birds

No	Species	Common name - English
1	*Struthio camelus camelus / c. massaicus*	Ostrich

2.3 Reptiles

No	Species	Common Name
1	*Acrantophis dumerili*	Dumeril's boa
2	*Acrantophis madagascariensis*	Ground boa
3	*Acrochordus javanicus*	Java wart snake
4	*Agkistrodon bilineatus*	Cantil
5	*Ahaetulla prasina*	Long nosed tree snake
6	*Anolis carolinensis*	Green anole
7	*Antaresia childreni*	Childrens python
8	*Antaresia maculosus*	Large blotched python
9	*Apodora papuana*	Papuan olive python
10	*Aspidites melanocephalus*	Black headed python
11	*Aspidites ramsayi*	Woma

12	*Atheris chlorechis*	West African bush viper
13	*Atheris nitschei*	Sedge viper
14	*Atheris squamigera*	Green/Common bush viper
15	*Basiliscus plumifrons*	Plumed basilisk
16	*Bitis nasicornis*	Rhinocerus viper
17	*Boa constrictor*	Boa constrictor
18	*Boiga blandingii*	Blandings treesnake
19	*Boiga dendrophila*	Mangrove treesnake
20	*Bothriechis lateralis*	Yellow lined palmviper
21	*Bothriechis marchi*	Honduran palmviper
22	*Bothriechis schlegelii*	Eyelash viper
23	*Bothrochilus boa*	Bismarck ringed python
24	*Bothrops alternatus*	Urutu
25	*Bothrops atrox*	Fer-de-lance
26	*Bothrops neuwiedi*	Neuwieds pitviper
27	*Boulengerina annulata*	Water cobra
28	*Brachylophus fasciatus*	Banded iguana
29	*Bungarus fasciata*	Banded krait
30	*Calabaria reinhardti*	Calabars burrowing python
31	*Calloselasma rhodostoma*	Malayan pitviper
32	*Candoia aspera*	Viper boa
33	*Candoia bibroni*	Pacific tree boa
34	*Candoia carinata*	Solomon island boa
35	*Cerastes vipera*	Sahara sand viper
36	*Chamaeleo jacksonii*	Jacksons chameleon
37	*Chamaeleo melleri*	Mellers chameleon
38	*Chondropython viridis*	Green tree python
39	*Chrysopelea ornate*	Flying snake
40	*Chrysopelea paradisi*	Paradise tree snake
41	*Corallus carinus*	Emerald tree boa
42	*Corallus cookii*	Cooks tree boa
43	*Corallus enhydris*	Garden tree boa
44	*Corucia zebrata*	Monkey tailed skink
45	*Crotalus adamanteus*	Eastern diamondback rattlesnake
46	*Crotalus basiliscus*	Mexican rattlesnake
47	*Crotalus cerastes*	Sidewinder
48	*Crotalus durissus*	Neotropical rattlesnake
49	*Crotalus enyo*	Californian rattlesnake
50	*Crotalus lepidus*	Rock rattlesnake
51	*Crotalus ruber*	Red diamondback rattlesnake
52	*Crotalus vegrandis*	Uracoan rattlesnake
53	*Cyclura cornuta*	Cuban iguana,
54	*Dasypeltis fasciata*	Banded eggeater
55	*Deinagkistrodon acutus*	Chinese sharp nosed viper
56	*Dendroaspis jamesoni*	Jamesons mamba
57	*Dendroaspis viridis*	West African green mamba
58	*Egernia major*	Land mullet
59	*Elaphe carinata*	Stinking goddess
60	*Elaphe climacophora*	Japanese ratsnake
61	*Elaphe mandarina*	Mandarin ratsnake
62	*Elaphe moellendorffi*	Hundred flower snake
63	*Elaphe porphyracea*	Bamboo ratsnake
64	*Elaphe prasina*	Green ratsnake
65	*Elaphe quadrivirgata*	Japanese four lined ratsnake
66	*Elaphe radiata*	Radiated ratsnake
67	*Elaphe schrenckii*	Russian ratsnake
68	*Elaphe taeniura*	Striped tailed ratsnake

69	*Elaphe vulpine*	Fox snake
70	*Epicrates angulifer*	Cuban boa
71	*Epicrates cenchria*	Rainbow boa
72	*Epicrates striatus*	Haiti boa
73	*Eristicophis macmahonii*	Leaf nosed viper
74	*Eunectes murinus*	Green anaconda
75	*Eunectes notaeus*	Yellow anaconda
76	*Furcifer oustaleti*	Oustelets chameleon
77	*Furcifer pardalis*	Panther chameleon
78	*Gehyra oceanica*	Giant island gecko
79	*Gehyra vorax*	Halmahera gecko
80	*Gekko monarchus*	Monarch gecko
81	*Gekko siamensis*	Chinese gecko
82	*Gekko smithii*	Smiths gecko
83	*Gekko vittatus*	White lined gecko
84	*Gonyosoma jansenii*	Jansens ratsnake
85	*Gonysoma oxycephalum*	Red-tailed green racer
86	*Heloderma horridum*	Beaded lizard
87	*Heloderma suspectum*	Gila monster
88	*Hemitheconyx caudicinctus*	Fat tailed gecko
89	*Hydrosaurus weberi*	Sailfin lizard
90	*Iguana delicatissima*	Antilles iguana
91	*Lachesis muta*	Bushmaster
92	*Langaha nasuta*	Leaf nosed snake
93	*Laticauda colubrinae*	Giant sea snake
94	*Laticauda laticauda*	Banded sea snake
95	*Leiopython albertisii*	White lipped python
96	*Liasis fuscus*	Water python
97	*Liasis mackloti*	Freckled python
98	*Morelia amethistina*	Amethistine python
99	*Morelia boeleni*	Boelens/black python
100	*Morelia bredli*	Bredli python
101	*Naja atra*	Chinese spitting cobra
102	*Naja kaouthia*	Monocled cobra
103	*Naja naja*	Spectacled cobra
104	*Naja oxiana*	Central Asian cobra
105	*Naja siamensis*	Siamese cobra
106	*Naja sputatrix*	Black and white spitting cobra
107	*Ophiophagus hannah*	King cobra
108	*Paroedura bastardi*	Madagascan ground gecko
109	*Paroedura picta*	Painted gecko
110	*Phelsuma comorensis*	Comoro day gecko
111	*Porthidium nummifer*	Jumping viper
112	*Proatheris superciliaris*	Lowland swamp viper
113	*Ptyas korros*	Brown ratsnake
114	*Python anchietae*	Angolan dwarf python
115	*Python breitensteini*	Short tailed python
116	*Python brongersmai*	Borneo python
117	*Python curtus*	Blood python
118	*Python molurus*	Indian/Burmese python
119	*Python regius*	Royal python
120	*Python reticulatus*	Reticulated python
121	*Rhacodactylus auriculatus*	Gargoyle gecko
122	*Rhacodactylus ciliatus*	Crested gecko
123	*Rhacodactylus leachianus*	New Caledonian gecko
124	*Sanzina madagascariensis*	Madagascan tree boa
125	*Sceloporus mala chiticus*	Emerald swift

126	*Spilotes pullatus*	Chicken snake
127	*Thamnophis radix*	Plains garter snake
128	*Thrasops jacksonii*	Jackson tree snake
129	*Tiliqua gerrardii*	Pinktongue skink
130	*Tiliqua gigas*	New Guinea bluetongue skink
131	*Tiliqua nigrolutea*	Blotched bluetongue skink
132	*Tiliqua occipitalis*	Western bluetongue skink
133	*Tiliqua scincoides*	Eastern bluetongue skink
134	*Trimeresurus albolabris*	White lipped tree viper
135	*Trimeresurus macrops*	Green tree viper
136	*Trimeresurus popeiorum*	Popes tree viper
137	*Trimeresurus purpureomaculatus*	Mangrove pitviper
138	*Trimeresurus trigonocephalus*	Sri Lankan tree viper
139	*Tropidolaemus wagleri*	Temple viper
140	*Uroplatus fimbriatus*	Common leaf tailed gecko
141	*Uroplatus henkeli*	Henkels leaf tailed gecko
142	*Uroplatus phantasticus*	Mossy tailed gecko
143	*Varanus komodoensis*	Komodo dragon
144	*Varanus salvator*	Indonesian water monitor

2.4 Amphibians

No species listed.

2.5 Fishes (Fresh-water)

Note: This is an existing permitted list that is still being vetted by specialists.

No	Species	Common Name
1	*Abramites hypselonotus*	Headstander
2	*Abramites microcephalus*	Marble headstander
3	*Acanthodoras cataphractus*	Painted talking catfish
4	*Acanthodoras spinosissimus*	Talking/spiny catfish
5	*Acanthodoras hancocki*	Hancock's catfish
6	*Acanthophthalmus javanicus*	Japanese loach/Javenese loach
7	*Acanthophthalmus kuhli*	Coolie/Leopard/Spined loach
8	*Acanthophthalmus myersi*	Slimy loach
9	*Acanthophthalmus semicinctus*	Half-banded loach
10	*Acanthophthalmus shelfordi*	Shelfords loach
11	*Acanthopsis choirorhynchus*	Longnose/horseface loach
12	*Acanthicus adoris*	
13	*Acuticurimath macrops*	Hooked-nose characin
14	*Adinia xenica*	Diamond killfish
15	*Aequidens aff dorsigerus*	Greencheeked aequidens
16	*Aequidens awani*	Golden ciclid
17	*Aequidens coeruleopunctatus*	Blue-point flag cichlid
18	*Aequidens curviceps*	
19	*Aequidens dorsicerus*	Red breasted cichlid
20	*Aequidens duopunctatus*	Double spot aequidens
21	*Aequidens hercules*	Hercules cichlid
22	*Aequidens itanyi*	Dolphin cichlid
23	*Aequidens latifrons*	Blue acara
24	*Aequidens metae*	Shunk acara
25	*Aequidens metae*	Shunk acara
26	*Aequidens maroni*	Keyhole cichlid
27	*Aequidens pallidus*	Pale cichlid
28	*Aequidens paraguayenis*	Cold cheeked flag c ichlid
29	*Aequidens portalegrensis*	Port/black acara
30	*Aequidens pulcher*	Blue acara
31	*Aequidens rivulatus*	Green terror
32	*Aequidens sapayensis*	Sapaya cichlid
33	*Aequidens* sp "gold saum"	Orange fringed terror
34	*Aequidens syspilum*	Somber flag cichlid
35	*Aequidens tetramerus*	Saddle/pishuna cichlid
36	*Aequidens thayeri*	Redbellied flag cichlid
37	*Aequidens vittatus*	Half banded flag cichlid

38	*Agamyxis costatus*	While line doradid
39	*Agamyxis pectinifrons*	White spotted doradid
40	*Agmus lyriformis*	Flatnose catfish
41	*Aconeiosus caucanus*	Lumpy banjo catfish
42	*Aconeiosus madeirensis*	Flathead catfish
43	*Aidaprora carteri*	
44	*Alepidomus evermanni*	Cuban glass fish
45	*Alestes chaperi*	Chaper’s characin
46	*Alestes longipinnis*	Long-finned characin
47	*Alestes nurse*	Nurse tetra
48	*Alestes taeniurus*	African/lined tetra
49	*Alestopetersius caudalis*	Yellow congo characin
50	*Alfaro cultratus*	Knife edged livebearer
51	*Ambassis (Chanada) lala*	Glassfish
52	*Amblydoras hancocki*	Talking catfish
53	*Anableps anableps*	Four eyes
54	*Ancistrus dolichopterus*	Bristle nose catfish
55	*Ancistrus hoplogenys*	Pearl sucker catfish
56	*Ancistrus lineolatus*	Bristle nose catfish
57	*Ancistrus multispinus*	Bristle nose catfish
58	*Ancistrus* sp “brown blotch”	Brown blotch bristlenose
59	*Ancistrus temmincki*	Temminck’s bristle nose catfish
60	*Ancistrus triradiatus*	Branched bristle nose catfish
61	*Anoptichthys jordani*	Blind cave tetra
62	*Anostomus anastomus*	Striped headstander
63	*Anostomus fasciatus*	Six-barred anostomus
64	*Anostomus gracilis*	Four-spot anostomus
65	*Anostomus taeniata*	Lisa
66	*Anostomus ternetzi*	Red-mojuth headstander
67	*Anostomus trimaculatus*	Three-spotted headstander
68	*Anostomus varius*	Checkerboard anostomus
69	*Apcreiodon pongoensis*	Pongo-pongo
70	*Aphyocharax alburnus*	Golden crowned aphyocharax
71	*Aphyocharax anisiti*	Bloodfin
72	*Aphyocharax axelrodi*	Travassos/calypso tetra
73	*Aphyocharax dentatus*	Falso bloodfin
74	*Aphyocharax erythrurus*	Flame tail tetra
75	*Aphyocharax rathburni*	Rathburn’s bloodfin
76	*Aphyocharax rubripinnis*	Bloodfin tetra
77	*Aphyocharax* sp “bleher”	Bleher’s bloodfin
78	*Aphyosemion ahli*	Ahl’s lyratail
79	*Aphyosemion arnoldi*	Arnold’s lyretail
80	*Aphyosemion australe australe*	Cape lopez/lyratail panchax
81	*Aphyosemion bertholdi*	Berthold’s killie
82	*Aphyosemion bitaeniatum*	Coppery killie
83	*Aphyosemion bivattatum bivattatum*	Red lyretail/two striped aphyosemion
84	*Aphyosemion breuningi*	Breuning’s killie
85	*Aphyosemion bualanum*	African swamp killie
86	*Aphyosemion calabaricus*	Calabar lyretail
87	*Aphyosemion calliurum ahli*	Ahl’s aphyosemion/blue calliurum
88	*Aphyosemion calliurum calliurum*	Red chinned aphyosemion
89	*Aphyosemion cameronensi*	Camaroon round tailed aphyosemion
90	*Aphyosemion celiae*	Celia’s aphyosemion
91	*Aphyosemion chaytori*	Chaytor’s killie
92	*Aphyosemion christyi*	Christy’s lyretail
93	*Aphyosemion cinnamomum*	Cinnamon killie
94	*Aphyosemion cognatum*	Red spotted/speckled killie
95	*Aphyosemion (diapteron) cyanostictum*	Blue spotted killfish
96	*Aphyosemion exigum*	Exiguum killie
97	*Aphyosemion fallax*	Ghana killie
98	*Aphyosemion filamentosum*	Plumed lyretail/togo lyretail
99	*Aphyosemion gubunense*	Yellowgreen killie
100	*Aphyosemion gardneri*	Steel blue aphiosemion
101	*Aphyosemion geryi*	Gery’s aphiosemion
102	*Aphyosemion guineense*	Guinea aphiosemion/geuinean killie
103	*Aphyosemion gulare caeruleum*	Blue gularis
104	*Aphyosemion gulare gulare*	Yellow gularis
105	*Aphyosemion kiyawense*	Loennberg’s killie
106	*Aphyosemion labarrei*	Labares aphyosemion
107	*Aphyosemion liberiense*	Liberian killie
108	*Aphyosemion loennbergi*	Lonnber’s aphyiosemion
109	*Aphyosemion marmoratum*	Green spangled chocolate killie
110	*Aphyosemion meinkerii*	Meinken’s aphyosemion
111	*Aphyosemion multicolor*	Many coloured lyretail
112	*Aphyosemion nigerianum*	Nigerian killie

113	*Aphyosemion occidentale*	Golden pheasant
114	*Aphyosemion oeseri*	Emerald aphyosemion
115	*Aphyosemion ogoense ottogartneri*	Broken striped killie
116	*Aphyosemion petersi*	Peter's round tailed aphyosemion/yellow-edged aphyosemion
117	*Aphyosemion puerzli*	Puerzls killfish
118	*Aphyosemion rectogoense*	Silver yellow killie
119	*Aphyosemion riggenbachi*	Red spotted purple killie
120	*Aphyosemion roloffi*	Rolff's round tailed aphyosemion
121	*Aphyosemion rubrilabiale*	Red lipped killie
122	*Aphyosemion scheeli*	Scheel's killfish
123	*Aphyosemion schiotzi*	Filamentous lyretail
124	*Aphyosemion schoultendeni*	Schouteden's aphyosemion
125	*Aphyosemion seymouri*	Seymour's killfish
126	*Aphyosemion sjoestedi*	Golden pheasant/blue throat aphyosemion/blue gular ?
127	*Aphyosemion splendopleure*	Splendid killie
128	*Aphyosemion spurrelli*	Turquoise killie
129	*Aphyosemion striatum*	Five lined killie
130	*Aphyosemion toddi*	Bluethroat killie
131	*Aphyosemion walkeri*	Walker's killfish
132	*Apistogramma agassizi*	Agassis dwarf cichlid
133	*Apistogramma amboplitoides*	Peruvian dwarf cichlid
134	*Apistogramma bitaeniaztum*	Two striped dwarf cichlid
135	*Apistogramma borelli*	Borelli's dwarf cichlid/umbrella dwarf cichlid
136	*Apistogramma cacatuoides*	Cockatoo dwarf cichlid
137	*Apistogramma corumbae*	Corumba dwarf cichlid
138	*Apistogramma eunotus*	B lue-cheek dwarf cichlid
139	*Apistogramma gibbiceps*	Yellow-cheeked dwarf cichlid
140	*Apistogramma gossei*	Brilliant dwarf cichlid
141	*Apistogramma hippolytae*	Empress dwarf cichlid
142	*Apistogramma klausewitzi*	Klauswitz dward cichlid
143	*Apistogramma kleyi*	B anded dwarf cichlid
144	*Apistogramma luelingi*	Golden dwarf cichlid
145	*Apistogramma macmasteri*	Red-tailed dwarf cichlid
146	*Apistogramma marmoratus*	
147	*Apistogramma ocellatus*	See astronotus occelatus
148	*Apistogramma ornatipinis*	
149	*Apistogramma ortmanni*	Ortman's dwarf cichlid
150	*Apistogramma pretense*	Yellow dwarf cichlid/amazon dwarf cichlid
151	*Apistogramma pleurotaenia*	Chequered dwarf cichlid
152	*Apistogramma ramirezi = Microgeophagus ramirezi*	Ramirez's/butterfloy dwarf cichlid/ram
153	*Apistogramma reitzigi*	Yellow dwarf cichlid/reitzig's dwarf cichlid
154	*Apistogramma steindachneri*	Steindachner's dwarf cichlid
155	*Apistogramma trifasciatum*	Blue apistogramma
156	*Apistogramma wesei*	Wise's dwarf cichlid
157	*Apistogramma wickleri*	Wickler's dwarf cichlid
158	*Apistogrammoides pucallprensis*	Dwarf cichlid
159	*Aplocheilichthys flavipinnis*	Yellow-finned lampeye
160	*Aplocheilichthys loemensis*	Loeme lampeye
161	*Aplocheilichthys macrophthalmus*	Lampeye/bigeye lampeye
162	*Aplocheilus slocki*	Dwarf/green panchax
163	*Aplocheilus dayi*	Ceylon killfish
164	*Aplocheilus lineatus*	Panchax lineatus/striped panchax
165	*Aplocheilus normani*	Norman's panchax
166	*Aplocheilus panchax*	Blue panchax
167	*Aplocheilus siamensis*	Panchax
168	*Apteronotus albifrons*	Black gost/black nose knifefish
169	*Apteronotus leptorhynchus*	Long nosed black ghost
170	*Aristochromis christus*	White top
171	*Arnoldichthys spilopterus*	Red-eye characin
172	*Asiphonichthys condei*	Glassy tetra/transparent tetra
173	*Aspidoras fuscoguttatus*	Spotted aspidoras
174	*Aspidoras lokoi*	Spot-line aspidoras/catfish
175	*Aspidoras pauciradiatus*	Fin blotch aspidoras
176	*Aspredo aspredo*	Slender banjo catfish
177	*Astronotus ocellatus*	Oscar/velvet cichlid/red oscar/peacock cichlid
178	*Astyanax abramis*	Confusing astyanax
179	*Astyanax bimaculatus*	Two-spot astyanax
180	*Astyanax daguae*	Plan-tailed astyanax
181	*Astyanax fasciatus fasciatus*	Sanded astyanax/blind cave tetra/Mexican tetra
182	*Astyanax kennedyi*	Kennedy's astyanax
183	*Astyanax metae*	Rio meta astyanax
184	*Astyanax mexicanus = Anoptichthys jordanii*	Mexican tetra (blind cave tetra)
185	*Astyanax mutator*	Mutato

186	*Astyanax* sp “big scale”	Big scale astyanax
187	*Astyanax zonatus*	False kennedy tetra
188	*Ataeniobius taweri*	Blue0-tailed gocoleid ?
189	*Auchenipterichthys thoracatus*	Zamora woodcat
190	*Auchenipterus demerare*	Demerara woodcat
191	*Auchitoglanis spp*	
192	*Aulonocara nyasse*	African peacock cichlid
193	*Aulonocara nyasse var*	Yellow peacock cichlid
194	*Aulonocara* sp “night aulonocara”	Night aulonocara
195	*Aulonocara* sp “white edged”	White edged aulonocara
196	*Austrofundulus dolichopterus = Cynolebias dolichopterus*	Sickelfin killie/saberfin
197	*Austrofundulus limnaeus*	Schultz’s spot finned killie
198	*Austrofundulus meyersi*	Meyer’s killifish
199	*Austrofundulus transilis*	Venesuela killfish
200	*Awaous grammepomus*	Strippled river coby
201	*Axelrodia lindeae*	Blushing tetra
202	*Axelrodia riesei*	Ruby tetra
203	*Axelrodia stigmatis*	Whit star tetra
204.	*Badis badis*	Badis/dwarf chameleon fish
205.	*Badis badis burmanicus*	Burmese badis
206.	*Bagrichthys hypselopterus*	Black lancer catfish
207.	*Balantiocheilos melanopterus*	Bala/tri-coloured/silver shark
208.	*Barbichthys hysclopterus*	Black lancer catfish
209.	*Barbichthys laevis*	Round/nose shark
210.	*Barbodes acrislongae*	Three-spotted barb
211.	*Barbodes arulius*	Long-finned barb
212.	*Barbodes binotatus*	Spotted barb
213.	*Barbodes callipterus*	Clipper barb
214.	*Barbodes chola*	Swamp barb
215.	*Barbodes comptacanthus*	African red-finned barb
216.	*Barbodes conchonius*	Rosy/red barb
217.	*Barbodes cumingi*	Cuming’s barb
218.	*Barbodes daruphani*	Pale barb
219.	*Barbodes dorsimaculatus*	Sumatra/black lined barb
220.	*Barbodes dunckeri*	Duncker’s/big spot barb
221.	*Barbodes everetti*	Clown barb
222.	*Barbodes fasciatus*	Striped barb
223.	*Barbodes filamentosus*	Black-spot barb
224.	*Barbodes gelius*	Golden barb
225.	*Barbodes guirali*	Redfin barb
226.	*Barbodes halei*	Hale’s barb
227.	*Barbodes hexazona*	Belted barb/six-banded barb
228.	*Barbodes holotaenia*	Spot-scale barb
229.	*Barbodes kostorii*	Koster’s barb
230.	*Barbodes lateristriga*	Spanner barb /T-barb
231.	*Barbodes macrops*	Slender black striped barb
232.	*Barbodes nicholsi*	Redfin black striped barb
233.	*Barbodes nigrofasciatus*	Black ruby/purple-headed barb
234.	*Barbodes oligolepis*	Checkered/island/tridecent barb
235.	*Barbodes pentazona pentazona*	Five banded barb
236.	*Barbodes pentazona rhomboocellatis*	Ocellatged barg
237.	*Barbodes pentazona rhomboocellata*	Round-banded barb
238.	*Barbodes phutunio*	Dwarf/pigmy barb
239.	*Barbodes prionocanthus*	Greater black-striped barb
240.	*Barbodes roloffi*	Roloff’s barb
241.	*Barbodes sachi*	Golden barb
242.	*Barbodes schuberti*	Schubert’s barb
243.	*Barbodes schwanenfeldi*	Tin-foil barb
244.	*Barbodes semifasciolatus*	Chinese/green/half-stripe barb
245.	*Barbodes setivimensis*	Algerian barb
246.	*Barbodes stoliczhanus*	Stolzicka’s barb
247.	*Barbodes terio*	One-spot barb
248.	*Barbodes tetrazona tetrazona*	Tiger/Sumatra barb
249.	*Barbodes ticto*	Tic tac toe/two-spot barb
250.	*Barbodes titteya*	Cherry barb
251.	*Barbodes trispilos*	African three-spot barb
252.	*Barbodes usambarae*	Peppered barb/algolan barb
253.	*Barbodes werneri*	Werner’s barb
254.	*Bario steindachneri*	Blotch-tailed barrio
255.	*Barombia* spp	
256.	*Bathethiops fanleri*	African moonfish
257.	*Bedotia geayi*	Madagascar rainbow fish
258.	*Belontia hasseltii*	Combtail/java combtail
259.	*Belontia signata*	Combtail paradise fish
260.	*Betta akarensis*	Sarawak betta

261.	*Betta bellica*	Slender/slim fighting fish
262.	*Betta brederi*	Javan mouth-brooding fighting fish
263.	*Betta coccina*	Red fighting fish/claret betta
264.	*Betta edithae*	Edith’s betta
265.	*Betta faseila*	Striped fighting fish
266.	*Betta imbellis*	Peaceful/crescent fighting fish
267.	*Betta macrostoma*	Peacock fighting fish/Brunei beauty
268.	*Betta pictum*	Javan fighting fish
269.	*Betta pugnax*	Penang mouth-brooding fighting fish ? (breeding)?
270.	*Betta smaragdina*	Emerald betta/peaceful betta
271.	*Betta splendens*	Libby’s betta/split-tailed/Siamese fighting fish
272.	*Betta taeniata*	Striped fighting fish
273.	*Betta unimaculata*	One-spot fighting fish
274.	*Biotodoma cupido*	Cupid cichlid
275.	*Bivibranchia bimacula*	Two-spot sandsucker
276.	*Bivibranchia protactila*	Silver sandsucker
277.	*Boehlkea fredcochui*	Cochu’s blue tetra
278.	*Boilius christy*	Copper nose
279.	*Boleophthalmus pectinirostris*	Comb-toothed mudskipper
280.	*Botia beaufortii*	Beauford’s loach
281.	*Botia bredmorei*	Bredmore’s loach
282.	*Botia dario*	Bengal loach
283.	*Botia horae*	Skunk/yellowtail loach/hora’s loach
284.	*Botia hymenophysa*	Bande/tiger loach
285.	*Botia lohachata*	Pakistani loach
286.	*Botia lecontei*	Red-tail loach
287.	*Botia lucas-bahi*	Barred loach
288.	*Botia macracanthas*	Clown loach
289.	*Botia modesta*	Orange finned blue loach
290.	*Botia morleti*	Hora’s loach
291.	*Botia pulchripinnis*	Red finned blue loach
292.	*Botia sidthimunki*	Dwarf loach
293.	*Botia striata*	Zebra loach
294.	*Brachydanio albolineatus*	Pearl/gold danio
295.	*Brachydanio frankei*	Leopard danio
296.	*Brachydanio kerri*	Kerrs danio
297.	*Brachydanio malabaricus*	Giant danio
298.	*Brachydanio nigrofasciatus*	Spotted danio
299.	*Brachydanio rerio*	Zebra/spotted danio
300.	*Brachydanio rerio var*	Long finned zebra danio
301.	*Brachydanio aggregatus*	Phillipine bumele bee fish
302.	*Brachydanio doriae*	Doria’s bumble bee fish
303.	*Brachydanio dorile*	Wasp goby
304.	*Brachydanio Brachydanio nunus*	Golden banded goby
305.	*Brachydanio xanthozona*	Bumble bee fish
306.	*Brachyrhaphis episcope*	Bishop
307.	*Brachyrhaphis dolminger*	Red-finned bishop
308.	*Brachyrhaphis terrabensis*	Upland live b earer
309.	*Brachyrhaphis rhabdophora*	Regan’s; bishop
310.	*Brienomyrus brachyistius*	Whale-faced marcusenius
311.	*Brittanichthys axelrodi*	Blood-red tetra
312.	*Brochis britskii*	Giant brochis
313.	*Brochis coeruleus*	Short-bodied catfish
314.	*Brochis multibradiatus*	Long-finned brochis/hob nosed brochis
315.	*Brochis splendens*	Short-bodied catfish
316.	*Bryanis longipinnis = Brycinus* spp	Long finend tetra
317.	*Bryanis nurse = Brycinus* spp	Nurse tetra
318.	*Bryanis taeniurius = Brycinus* spp	Lined tetra = syn. Brycinus spp
319.	*Brycinus chaperi*	Chaper’s characin
320.	*Brycinus longipinnis = Bryanis longipinnis*	Long-finned African tetra
321.	*Brycinus nurse = Bryanis nurse*	Nurse tetra
322.	*Brycinis taeniurus*	Lined African tetra
323.	*Brycon brevicauda*	Short-tailed troud tetra
324.	*Brycon cephalus*	Black-tailed brycon
325.	*Brycon melanopterus*	Sickle-band brycon
326.	*Bryconamericus loisae*	Pico peixe
327.	*Bryconops affinis*	Red-tipped tetra
328.	*Bryconoops caudomaculatus*	Red cross tetra
329.	*Bryconops melanurus*	Jumping tetra-jumping anchovy
330.	*Bunocephalus amaurus*	Banjo catfish
331.	*Bunocephalus coracoideus*	Two-coloured banjo catfish
332.	*Bunocephalus spp*	
333.	*Bunocephalus knerii*	Kner’s banjo catfish
334.	*Caenotropus maculosus*	Slender headsgtander
335.	*Calamoichthys calabricus*	Rope/snake fish

336.	*Callichthys calabricus*	Armoured catfish
337.	*Callichthys callichthys*	Slender armored catfish
338.	*Callophysus macropterus*	Callophysus
339.	*Campylomormyrus cassaicus*	Angolan elephantnose
340.	*Campylomormyrus rhyncophorus*	Down-poker
341.	*Campylomormyrus tamandua*	Worm jawed mormyrid
342.	*Capoeta arulius*	Long-finned barb
343.	*Capoeta chola*	Swamp barb
344.	*Capoeta conchonus*	Rosy barb
345.	*Capoeta fasciatus = Capoeta melanampyx*	Ember barb
346.	*Capoeta hulstaerti*	Butterfly barb
347.	*Capoeta melanampyx = Capoeta fasciatus*	Ember barb
348.	*Capoeta nigrofasciatus*	Nigger barb
349.	*Capoeta oligolepis*	Chequered barb
350.	*Capoeta partipentazona*	Banded barb
351.	*Capoeta puckelli*	Two-spot African barb
352.	*Capoeta semifasciolatus*	Semi barb/half-striped barb
353.	*Capoeta somphongs*	Somphong's barb
354.	*Capoeta tetrazona*	Tiger bars
355.	*Capoeta titteya*	Cherry barb
356.	*Carassius auratus auratus*	Gold fish (all varieties)
357.	*Cariotetraodon somphongsi*	Somphong's puffer fish
358.	*Carlastyanax aurocaudatus*	Gold-tailed tetra
359.	*Carlhubbsia stuarti*	Banded widow
360.	*Carnegiella marthae*	Black winged hatchet fish
361.	*Carnegiella meyersi*	Marbled hatchet fish
362.	*Carnegiella strigiata fasciata*	Marbled banded hatchet fish
363.	*Carnegiella strigiata strigiata*	Marbled hatchet fish
364.	*Carnegiella vesca*	Marbled hatchet fish
365.	*Centromochlus heckeli*	Heckel's woodcat
366.	*Chaenobryttus culosus*	
367.	*Chaetobranchus bitaeniatus*	Two-striped cichlid
368.	*Chaetobranchus flavescens*	Red-eye cichlid
369.	*Chalceus erythrurus*	Yellow-finned chalceus
370.	*Chalceus macrolepidotus*	Pink-tailed characin
371.	*Chalinochromis brichardi*	Brichard's chalinochromis
372.	*Chanda asiatica*	
373.	*Chanda baculis*	Malaysian veiltailed glass fish
374.	*Chanda buruensis*	Siamese/east Indian glass fish
375.	*Chanda lala*	Glass fish
376.	*Chanda nama*	Elongated glass fish
377.	*Chanda ranga*	Indian glass fish/glass perch
378.	*Chanda saculis*	Glass fish
379.	*Characidium fasciatum*	Banded characidium
380.	*Characidium* sp "Gery"	"Gery's" characidium
381.	*Cheirodon axelrodi*	Cardinal tetra
382.	*Cheirodon kreigi*	Three-spot tetra
383.	*Chilatherina axelrodi*	Axelrod's rainbowfish
384.	*Chilatherina campsi*	Highlands rainbowfish
385.	*Chilatherina crassispinosa*	Silver rainbowfish
386.	*Chilatherina fasciata*	Barred rainbowfish
387.	*Chilodus punctatus*	Spotted/pearl headstander
388.	*Chilodus* sp "Black band"	Balack banded headstander
389.	*Chilogobio czerskii*	Rainbow gudgeon
390.	*Chilotilapia rhoades*	Rhoades chilo
391.	*Chilotilapia* spp	
392.	*Chiopeops goodie*	Blue-fin top
393.	*Cichlasoma altifrons*	Hi-head cichlid
394.	*Cichlasoma atromaculatum*	Three-spot cichlid
395.	*Cichlasoma aureum*	Golden cichlid
396.	*Cichlasoma axelrodi*	Black chuco
397.	*Cichlasoma bartoni*	Barton's cichlasoma
398.	*Cichlasoma bifasciatum*	Red spotted cichlid/usmacinta cichlid
399.	*Cichlasoma bimaculatum*	Two-spot cichlid
400.	*Cichlasoma biocellatum*	Jack Dempsey
401.	*Cichlasoma carpintis*	Pearlscale cichlid
402.	*Cichlasoma centrachus*	Flier cichlid
403.	*Cichlasoma citrinellum*	Midas cichlid
404.	*Cichlasoma coryphaenoides = Cichlasoma carpinis*	Chocolate cichlid
405.	*Cichlasoma crassa*	Flame cichlid
406.	*Cichlasoma cutteri*	Cutter's cichlid
407.	*Cichlasoma dovii*	Dows cichlid
408.	*Cichlasoma erythraem*	Red devil
409.	*Cichlasoma facetum*	Chanchito/chameleon cichlid
410.	*Cichlasoma festae*	Festae cichlid

411.	*Cichlasoma festivum*	Flag/banded cichlid
412.	*Cichlasoma grammodes*	Many-pointed cichlid
413.	*Cichlasoma guttulatum*	Gold-cheek cichlid
414.	*Cichlasoma hartwegi*	Hartweg's cichlid
415.	*Cichlasoma hellabruni*	Hellabrun's/chocolaty cichlid
416.	*Cichlasoma intermedium*	Jordan's cichlid
417.	*Cichlasoma kraussi*	Sharphead cichlid
418.	*Cichlasoma labiatum*	Red devil
419.	*Cichlasoma longimanus*	Longfin cichlasoma
420.	*Cichlasoma macracanthus*	Highspine cichlid
421.	*Cichlasoma maculate*	Chessboard cichlid
422.	*Cichlasoma managuense*	Managuense
423.	*Cichlasoma meeki*	Firemouth cichlid
424.	*Cichlasoma minkleyi*	Minkley's cichlid
425.	*Cichlasoma nicaraguense*	Spilotum
426.	*Cichlasoma nigrofasciatum*	Convict/zebra cichlid
427.	*Cichlasoma psittacum*	Parrot cichlid
428.	*Cichlasoma robertsoni*	Robertson's cichlid
429.	*Cichlasoma sajica*	t-bar convict
430.	*Cichlasoma salvini*	Yellow belly/salvinis cichlid
431.	*Cichlasoma septemfasciatum*	Cutter's cichlid
432.	*Cichlasoma severum*	Deacon/banded cichlid
433.	*Cichlasoma spectabile*	
434.	*Cichlasoma spilurum*	Blue-eyed cichlid
435.	*Cichlasoma synspilum*	Red-headed cichlid
436.	*Cichlasoma temporale*	Flag/golden cichlid
437.	*Cichlasoma tetracanthum*	Cuban cichlid
438.	*Cichlasoma tuyrense*	Tuyre cichlid
439.	*Cichlasoma umbriferum*	Umbriferum
440.	*Cichlasoma zonatum*	Dappled cichlid
441.	*Citharinus citharus*	Lined citharinid
442.	*Cleithrochromis bowleyi*	Mae west cichlid
443.	*Cnesterodon decemmaculatus*	Ten spotted livebearer
444.	*Cochiodon plecostomoides*	Spoonmouth sucker catfish
445.	*Cochliodon hondae*	Hondae cochliodon
446.	*Coelurichthys microlepis*	Croaking tetra
447.	*Coelurichthys tenuis*	Tenuis tetra
448.	*Colisa chuna*	Honeycomb gourami
449.	*Colisa fasciata*	Giant/striped/banded gourami
450.	*Colisa labiosa*	Thick lipped gourami
451.	*Colisa lalia*	Dwarf gourami
452.	*Colomesus asellus*	Brazilian freshwater puffer
453.	*Colomesus psittacus*	Parrot/South American puffer fish
454.	*Copella arnoldi*	Splash tetra/spraying characin
455.	*Copella callolepis*	False splashing tetra/spotted/beautiful scaled charac
456.	*Copella eicenmanni*	Eigenmann's copella
457.	*Copella metae. = Copella nigrofasciata*	Brown-banded copella
458.	*Copella multispinis*	
459.	*Copella nattereri*	Plain nattereri/spotted cobella
460.	*Copella nigrofasciata = Copella metae*	Brown-banded copella
461.	*Copella vilmae*	Rainbow copella
462.	*Copeina cuttata*	Red-spotted characin
463.	*Corydoras acutus*	Black top catfish
464.	*Corydoras adulfoi*	Adolfo's cory
465.	*Corydoras aeneus*	Bronze catfish
466.	*Corydoras agassizi*	Agassizi's catfish
467.	*Corydoras aldino*	Albino catfish
468.	*Corydoras ambiacus*	Half-masked cory
469.	*Corydoras arcuatus*	Skunk/arched catfish
470.	*Corydoras armatus*	
471.	*Corydoras atropersonatus*	Masked cory
472.	*Corydoras axelrodi*	Axelrod's cory
473.	*Corydoras barbatus*	Banded catfish
474.	*Corydoras bondi*	Bond's cory
475.	*Corydoras caquetae*	
476.	*Corydoras caudimaculatus*	Tail-spot catfish
477.	*Corydoras cervinus*	
478.	*Corydoras cochui*	Cochus catfish
479.	*Corydoras decker*	
480.	*Corydoras delphax*	Delphax
481.	*Corydoras erhardti*	Erhardt's cory
482.	*Corydoras elegans*	Elegant catfish
483.	*Corydoras eques*	Golden eared cory
484.	*Corydoras evelynae*	Evelyn's cory
485.	*Corydoras garbei*	Garbei

486.	*Corydoras gracilis*	Pretty dwarf cory
487.	*Corydoras griseus*	Grey catfish
488.	*Corydoras guapore*	Guapore cory
489.	*Corydoras harbrosus*	Rio Salinas cory
490.	*Corydoras haraldschultzi*	Herald schultz's cory
491.	*Corydoras hastatus*	Dwarf/pigmy catfish
492.	*Corydoras juli*	Leopard catfish
493.	*Corydoras latus*	Iridescent cory
494.	*Corydoras leopardus*	Leopard catfish
495.	*Corydoras leucomelas*	Blackfin cory
496.	*Corydoras loxozonus*	Slant-bar cory
497.	*Corydoras macropterus*	Long-fin cory
498.	*Corydoras melanistius*	Black-spotted catfish
499.	*Corydoras melanotaenia*	Blackband cory
500.	*Corydoras melini*	Striped catfish
501.	*Corydoras metae*	Masked/bandit catfish
502.	*Corydoras microps*	Light spot catfish
503.	*Corydoras yersi*	Myer's catfish
504.	*Corydoras multimaculatus*	Soldier catfish
505.	*Corydoras nanus*	Mini cory
506.	*Corydoras napoensis*	Napo cory
507.	*Corydoras narcissus*	Narcissus cory
508.	*Corydoras nattereri*	Blue catfish
509.	*Corydoras ornatus*	Ornate cory
510.	*Corydoras orphnopterus*	Spotfin cory
511.	*Corydoras osteocarus*	Pepper-spot cory
512.	*Corydoras paleatus*	Peppered catfish
513.	*Corydoras panda*	Panda cory
514.	*Corydoras pastazensis*	Pastaza cory
515.	*Corydoras polystictus*	Mini spotted cory
516.	*Corydoras pulcher*	Dash-line cory
517.	*Corydoras punctatus*	Spotted catfish
518.	*Corydoras pygmaeus*	Pycmy catfish
519.	*Corydoras rabauti*	Dwarf/rabaut's catfish
520.	*Corydoras reticulates*	Network/reticulated catfish
521.	*Corydoras robinae*	Robina's cory
522.	*Corydoras sanchesi*	Sachez's cory
523.	*Corydoras schwartzi*	Schwartz's catfish
524.	*Corydoras semiaquilus*	Sharp-nosed cory
525.	*Corydoras septentrionalis*	Southern green cory
526.	*Corydoras simulates*	Copy catfish
527.	*Corydoras spilurus*	
528.	*Corydoras sterbai*	Fat catfish
529.	*Corydoras sychri*	Sychri
530.	*Corydoras treitlii*	Longnose catfish
531.	*Corydoras trilineatus*	Three line cory
532.	*Corydoras undulates*	Wavy catfish
533.	*Corydoras xinguensis*	Rio xingu cory
534.	*Corydoras zygatus*	Zygnatus
535.	*Corynopoma riisei*	Longnose catfish/swordtail charaein
536.	*Craetochanes caudomaculatus*	Tail-spot tetra
537.	*Craetochanes inaequalis*	Orange finend characin
538.	*Craterocephalus stercusmuscarum*	Fly-specked hardyhead
539.	*Creagrutus beni*	Gold striped characin
540.	*Creagrutus cochui*	Fred cochu's tetra
541.	*Crenicara adolfi*	Adolfis cichlid
542.	*Crenicara filamentosa*	Checkerboard lyretail
543.	*Crenicara maculate*	Checkerboard cichlid
544.	*Crenicara punculata*	Hercules cichlid
545.	*Crenuchus spiluris*	Sailfin tetra/sailfin characin
546.	*Ctenobrycon spiluris*	Silver tetra
547.	*Ctenopoma acutirostre*	Spotted/leopard climbing perch
548.	*Ctenopoma ansorgei*	Ornate ctenopoma
549.	*Ctenopoma congicum*	Congo climbing perch
550.	*Ctenopoma fasciolatum*	Banded climbing perch
551.	*Ctenopoma kingsleyae*	Tail-spot climbing perch
552.	*Ctenopoma maculate*	Single spot ctenopom
553.	*Ctenopoma nanum*	Dwarf climbing perch
554.	*Ctenopoma nigropannosum*	Two-spot climbing perch
555.	*Ctenopoma ocellatum*	Eye-spot climbing perch
556.	*Ctenopoma oxyrhynchum*	Marbled/sharp-nose climbing perch
557.	*Ctenpoma* spp	
558.	*Ctenops pumilis*	Pigmy gourami
559.	*Ctenops schalleri*	Three-striped gourami
560.	*Ctenops vittatus*	Croaking gourami

561.	*Cubanichthys cubensis*	Cuban killie
562.	*Curimata ciliate*	Hairy curimata
563.	*Curimata elegans*	Spotted cirimata
564.	*Curimata isognatha*	Chunkey curimata
565.	*Curimata microcephala*	Curimata
566.	*Curimata rhomboids*	Silver curimata
567.	*Curimata spilura*	Diamond-spot curimata
568.	*Curimata vittata*	Banded curimata
569.	*Curimatella alburna*	Common curimata
570.	*Curimatopsis evelynae*	Evelyn axelrod's curimata
571.	*Curimatopsis macrolepis*	Shiny scaled curimata
572.	*Cyathochromis* spp	
573.	*Cyclocheilichthys apogon*	Indian river barb/skin-head barb
574.	*Cynolebias adolfi*	Adolff's pearl fish
575.	*Cynolebias alexandri*	Alexander's pearl fish
576.	*Cynolebias antenori*	Red-finned pearl fish
577.	*Cynolebias bellotti*	Argentine pearl fish
578.	*Cynolebias boitonei*	Brazilian lyrefin
579.	*Cynolebias brucei*	Turner's gaucho
580.	*Cynolebias dolichofterus = Austrofunculus dolichopterus*	Sicklefin killie
581.	*Cynolebias melanotaenia*	Fighting gaucho
582.	*Cynolebias nigripinnis*	Black-finned pearl fish
583.	*Cynolebias porosus*	Big-lip pearl fish
584.	*Cynolebias wolterstorffi*	Wolterstorff's pearl fish
585.	*Cynopoecilus ladigesi*	Ladigesi gaucho
586.	*Cynopoecilus melanothaenia*	Fighting gaucho
587.	*Cynatilapia afra*	Dwarf zebra
588.	*Cynotilapia axelrodi*	Axelrod's Dogtooth cichlid
589.	*Cynotilapia frontosa*	Frontosa
590.	*Cynotilapia* spp	
591.	*Cynotilapia* spp	
592.	*Cyprichromis brient*	Slender cichlid
593.	*Cyprichromis leptosoma*	Slender cichlid
594.	*Cyprinus carpio*	Koi carp
595.	*Danio aequipinnatus*	Giant danio
596.	*Danio devario*	Bengal danio
597.	*Danio malabaricus*	Giant danio
598.	*Dasyloricaria filamentosa*	Rineloricaria filamentosa
599.	*Dermogenys pusillus*	Malayan halfbeak
600.	*Dermogenys sumatrans*	
601.	*Dianema longibarbis*	Porthole catfish
602.	*Dianema urostriata*	Striped-tail catfish
603.	*Dinopterchis* spp	
604.	*Distichodus affinis*	Silver/Red-finned distochodus
605.	*Distichodus decemmaculatus*	Dwarf distichodus
606.	*Distichodus fasciolatus*	Shark-tailed distichodus
607.	*Distichodus lusosso*	Long-nose distichodus
608.	*Distichodus noboli*	Nobol
609.	*Distichodus sexfasciatus*	Six-barred distichodus
610.	*Distocyclus conirostris*	Yellow knife fish
611.	*Doras eigenmannii*	Eicenmann's doradid
612.	*Dorichthys retzi*	Ragged-tail pipefish
613.	*Dormitator latifrons*	Western/broadhead sleeper goby
614.	*Dormitator maculatus*	Spotted sleeper goby
615.	*Duopalathinus barbatus*	Swallowtail pimelodid
616.	*Duopalatinus goeldii*	Goeld's pimelodid
617.	*Ectodus* spp	
618.	*Eigenmannia virescens*	Green knifefish
619.	*Eirmotus octozona*	False barb
620.	*Elachocharax junki*	Junk's darter tetra
621.	*Elachocharax pulcheer*	Dwarf darter tetra
622.	*Entomocorus benjamini*	Benjamin woodcat
623.	*Epalzeophyncus kalopterus*	Flying fox
624.	*Epalzeorhyncus siamensis*	Siarese flying fox
625.	*Ephippicharax orbicularis*	Salmon discus
626.	*Epicyrtos microlepsis*	Glass characin
627.	*Epiplatys annulatus*	Clown killie/Rocket panchax
628.	*Epiplatys fasciatus*	Two-striped panchax
629.	*Epiplatys chaperi*	Firemouth/Orange throated panchax
630.	*Epiplatys chevalieri*	Chevalier's epiplatys
631.	*Epiplatys dageti*	Black-lipped panchax/Red-chinned panchax
632.	*Epiplatys dageti monroviae*	Arnold's killie
633.	*Epiplatys dorsalis*	Jewelled Epiplatys
634.	*Epiplatys duboisi*	Dubois Epiplatys
635.	*Epiplatys esekans*	

636.	*Epiplatys fasciolatus*	Banded epiplatys/Striped panchax
637.	*Epiplatys grahami*	Graham's epiplatys
638.	*Epiplatys huberi*	Green-bellied panchax
639.	*Epiplatys longiventralis*	Banded panchax
640.	*Epiplatys macrostigma*	Large spotted panchax
641.	*Epiplatys nigromarginates*	Black-edge epiplatys
642.	*Epiplatys njalensis*	Red-spotted panchax
643.	*Epiplatys ornatus*	Emerald epiplatys
644.	*Epiplatys senegalensis*	Senegal epiplatys
645.	*Epiplatys sexfasciatus*	Six-barred epiplatys/Green panchax
646.	*Epiplatys sheljuzhkoi*	Sheljuzhkoi panchax
647.	*Epiplatys singa*	One-bar panchax
648.	*Epiplatys spilargyreius*	Green Panchax
649.	*Erpetoichthys calabaricus*	Reed fish
650.	*Esomus danrica*	Flying barb
651.	*Esomus malayensis*	Malawi flying barb
652.	*Esomus metallicus*	Silver flying barb
653.	*Etroplus maculatus*	Orange chromide
654.	*Eutropiellus debauwi*	Three-striped glass catfish
655.	*Eutropiellus* spp	
656.	*Exodon paradoxus*	Bucktooth characin
657.	*Farlowella acus*	Twig catfish
658.	*Farlowella amazonica*	Twig catfish
659.	*Farlowella gracilis*	Twig catfish
660.	*Gagata cenia*	
661.	*Carmanella pulchra*	Yucatin pupfish
662.	*Garra lamta*	
663.	*Garra taeniata*	Stone lapping fish
664.	*Gasteropelecus levis*	Giant silver hatchet fish
665.	*Gasteropelecus maculatus*	Spotted hatchet fish
666.	*Gasteropelecus sternicla*	Silver hatchet fish
667.	*Geophagus acuticeps*	Cutic
668.	*Geophagus balzanii*	Paraguay mouthbrooder
669.	*Geophagus braziliensis*	Peal cichlid/Brazilian high hat
670.	*Geophagus daemon*	Slender Geophagus
671.	*Geophagus hondae*	Redump gephagus
672.	*Geophagus jurupari*	Eartheater/Demon Fish
673.	*Geophagus rhabdotus*	Pearl striped geophagus
674.	*Geophagus steindachneri*	Redhump geophagus
675.	*Geophagus surinamensis*	Surinam geophagus
676.	*Geophagus thayeri*	Eartheater
677.	*Gephyrocharax atrocaundatus*	Platinum tetra
678.	*Gephyrocharax caucanus*	Arrowhead tetra
679.	*Gephyrochromis lawsi*	Violet cichlid
680.	*Gephyrochromis moorii*	Yellow-tailed violet cichlid
681.	*Gephyrochromis* spp	
682.	*Girardinus metallicus*	Giradimus
683.	*Glanucauda ?nequalis*	Croaking tetra
684.	*Glaridichthys falcatus*	Yellow belly
685.	*Glossamia sandei*	Giant cardinalfish
686.	*Glossamia trifasciata*	Three-banded cardinalfish
687.	*Glossamia wichmanni*	Wichmann's gardinalfish
688.	*Glossolepis incisus – melactaenia incisus*	Red rainbowfish
689.	*Glossolepis maculosus*	Spotted rainbowfish
690.	*Glossolepis multisquamatus*	Sepik rainbowfish
691.	*Glossolepis wanamensis*	Lake wanam rainbowfish
692.	*Gnathobagrus depressus*	Golden flathead catfish
693.	*Gnathonemus moori*	
694.	*Gnathonemus petersii*	Peter's elephant-nose fish
695.	*Gnathonemus shilthuis*	Elephant trunk fish
696.	*Gnathonemus tamandua*	Worm-jawed mormyrid
697.	*Gnathopogon chankaensis*	Amur minnow
698.	*Gobiopterus chuno*	Glass goby
699.	*Gobius jonklaasi = Sicyopterus jonklaasi*	Mountain goby
700.	*Gobius sadanundio = Stigmatogobius sadanundio*	The knight goby
701.	*Gobius vaimosa*	
702.	*Gymnocorymbus ternetzi*	Black tetra/Black widow/Petticoat fish
703.	*Gymnocorymbus thayeri*	Straight finned black tetra
704.	*Gymnorhamphichthys hypostomus*	Long-nosed knife fish
705.	*Gymnorhamphichthys rondoni*	Mousetail knife fish
706.	*Gymnotis anguillaris*	Slant-bar knife fish
707.	*Gymnotis carapo*	Banded knife fish
708.	*Gyrinocheilus ymonieri*	Algae eater
709.	*Halophryne trispinosus*	Todfish/Freshwater lionfish
710.	*Hampala macrolepidota*	Sidebar barb

711.	*Haplochromis ahli*	
712.	*Haplochromis of fenestratus*	Fenestratus no. 2
713.	*Haplochromis of labridens*	Black-striped greed hap
714.	*Haplochromis of taeniolatus*	Blue-faced dusky hap
715.	*Haplochromis compressiceps*	Malawian eye-biter
716.	*Haplochromis electra*	Deepwater mouthbrooder
717.	*Haplochromis euchilus*	Big lips
718.	*Haplochromis fenestratus*	Fenestratus
719.	*Haplochromis fuscoteniatus*	Fascotoeniatus
720.	*Haplochromis horei*	Spotted haplochromis
721.	*Haplochromis kingsleyae*	
722.	*Haplochromis labrosus*	Labrosus
723.	*Haplochromis linni*	Elephant-nose polystigma
724.	*Haplochromis livingstoni*	Livingstoni
725.	*Haplochromis mloto*	Ivory-topped hap
726.	*Haplochromis moori*	Blue moori/blue lumphead
727.	*Haplochromis polystigma*	Pollystigma
728.	*Haplochromis rostratus*	Rostratus
729.	*Haplochromis similis*	Red empress
730.	*Haplochromis* sp “electric blue”	Electric blue hap
731.	*Haplochromis spilonotus*	Spilonotus
732.	*Haplochromis strigatus*	Broken-striped hap
733.	*Haplochromis venustus*	Venustus
734.	*Haplochromis virginalis*	Yellow-finned hap
735.	*Haplochromis wingati*	Nigerian mouth brooder
736.	*Hara hara*	
737.	*Hasemania marginata*	Silver tips
738.	*Hasemania melania*	Copper characin
739.	*Hasemania nana*	Silver-tipped tetra
740.	*Hassar notospilus*	Black-finned doradid
741.	*Helogenes marmoratus*	Marbled helogenes
742.	*Helostoma rudolfi*	Kissing gourami
743.	*Helostma temmincki*	Green kissing gourami
744.	*Hemiancistrus nicefordi*	Clown sucker catfish
745.	*Hemiancistrus vittatus*	Striped sucker catfish
746.	*Hemibrycon guppyi*	Guppy’s characin
747.	*Hemibrycon tridens*	Jumping anchovy
748.	*Hemibrycon thomasi = Pelmatochromis thomasi*	African butterfly cichlid
749.	*Hemigrammocypris lini*	Garner tetra
750.	*Hemigrammopetersius caudalis*	Yellow-tailed congo tetra
751.	*Hemigrammopetersius intermedius*	Short-finned congo tetra
752.	*Hemigrammus armstrongi*	Golden tetra
753.	*Hemigrammus belottii*	Dash-Dot tetra
754.	*Hemigrammus boesemani*	Boeseman’s tetra
755.	*Hemigrammus coeruleus*	Cerulean tetra
756.	*Hemigrammus erythrozonus*	Glowlight tetra
757.	*Hemigrammus gracilis*	Glowlight tetra
758.	*Hemigrammus hyanuary*	January tetra
759.	*Hemigrammus levis*	Golden neon
760.	*Hemigrammus luelingi*	Lueling’s tetra
761.	*Hemigrammus marginatus*	Bassam tetra
762.	*Hemigrammus mattei*	Slender head and tail light
763.	*Hemigrammus nanus*	Silver tip tetra
764.	*Hemigrammus ocellifer*	Head-and-tail tetra
765.	*Hemigrammus proncki*	Pronck’s tetra
766.	*Hemigrammus pulcher*	Garnet/pretty tetra/black wedge
767.	*Hemigrammus rhodostomus*	Red/rummy-nose tetra – H. Bleheri
768.	*Hemigrammus rodwayi*	Gold/Rodway’s tetra
769.	*Hemigrammus schmardae*	Schmards tetra
770.	*Hemigrammus ulreyi*	Ulrey’s tetra
771.	*Hemigrammus unilineatus*	One-lined/Featherfin tetra
772.	*Hemigrammus vorderwinkleri*	Vorderwinkler’s tetra
773.	*Hemiodontichthys acipenserinus*	Knobnose whiptail catfish
774.	*Hemiodopsis goeldii*	Goeld’s hemiodus
775.	*Hemiodopsis gracilis*	Slender hemiodus
776.	*Hemiodopsis immacalatus*	Silver hemiodus
777.	*Hemiodopsis microlepis*	Red-tailed hemiodus
778.	*Hemiodopsis parnaguae*	One-spor hemiodus
779.	*Hemiodopsis quadrimaculatus*	Barred hemiodus
780.	*Hemiodopsis semitaeniatus*	Half-lined hemiodus
781.	*Hemiodopsis sterni*	Sterns hemiodus
782.	*Hemiodopsis unimaculatus*	Yellow-tailed hemiodus
783.	*Hemiodopsis chrysopunctatus*	Gold-spot halfbeak
784.	*Hemiodopsis pongonognathus*	Longfinned halfbeak
785.	*Hephaestus carbo*	Coal grunter

786.	*Heterandia formosa*	Dwart mosquito fish
787.	*Heterophallus rachovi*	Rachow's livebearer
788.	*Hippichthys spicifer*	Belly-barred pipefish
789.	*Holobrycon pesu*	Mourning tetra
790.	*Homoloptera orthogoniata*	Saddlespot loach
791.	*Homoloptera of zollingeri*	Zollinger's hillstream loach
792.	*Hoplerythrinus unitaeniatus*	Golden thahira
793.	*Hoplosternum littorale*	Cuscudo
794.	*Hoplosternum pectorale*	Spotted hoplo
795.	*Hoplosternum thoractum*	Port hoplo/hoplo cat/atipa
796.	*Horandandia atukorali*	Midget minnow/Green Carpet
797.	*Hypancistrus zebra*	
798.	*Hyphessobrycon of bifasciatus*	Yellow tetra
799.	*Hyphessobrycon agulha*	Flag tetra
800.	*Hyphessobrycon bellottii*	Dash-dot tetra
801.	*Hyphessobrycon bentosi*	Rosy tetra
802.	*Hyphessobrycon bifasciatus*	Yellow/Bronze tetra
803.	*Hyphessobrycon callistus serpae*	Serpae tetra
804.	*Hyphessobrycon callistus minor*	Minor tetra
805.	*Hyphessobrycon cardinalis*	Cardinal tetra
806.	*Hyphessobrycon copelandi*	Copeland's tetra
807.	*Hyphessobrycon eos*	Dawn tetra
808.	*Hyphessobrycon erythrostigma*	Bleeding heart tetra
809.	*Hyphessobrycon erythrozones*	Glowlight tetra
810.	*Hyphessobrycon erythurus*	Red-tailed tetra
811.	*Hyphessobrycon flammeus*	Flame tetra/tetra von rio
812.	*Hyphessobrycon greimi*	Griem's tetra
813.	*Hyphessobrycon haroldschultzi*	Harold schultz's tetra
814.	*Hyphessobrycon herbertaxelrodi*	Black neon tetra
815.	*Hyphessobrycon heterorhabdus*	Flag tetra
816.	*Hyphessobrycon innesi*	Neon tetra
817.	*Hyphessobrycon loretoensis*	Tetra loreto
818.	*Hyphessobrycon luetheni*	Luthen's tetra
819.	*Hyphessobrycon maculicauda*	Tail spot tetra
820.	*Hyphessobrycon metae*	Purple tetra
821.	*Hyphessobrycon minimus*	Dwarf tetra
822.	*Hyphessobrycon nigrifrons*	Black crowned tetra
823.	*Hyphessobrycon omatus*	Rosy tetra
824.	*Hyphessobrycon ornatus*	Ornate tetra
825.	*Hyphessobrycon peruvianus*	Loreto tetra
826.	*Hyphessobrycon pulchirpinnis*	Lemon tetra
827.	*Hyphessobrycon reticulatis*	Netted tetra
828.	*Hyphessobrycon* sp	Robert's tetra
829.	*Hyphessobrycon rubrostigma*	Bleeding heart/perez tetra
830.	*Hyphessobrycon saizi*	Saiz's tetra
831.	*Hyphessobrycon scholzei*	Black-lined tetra
832.	*Hyphessobrycon serpae-haraldschultzi*	
833.	*Hyphessobrycon serpae-serpae*	Serpae tetra
834.	*Hyphessobrycon simulans = Paracheirodon simulans*	False tetra
835.	*Hyphessobrycon socolofi*	Lesser bleeding heart
836.	*Hyphessobrycon stegemanni*	Savanna tetra
837.	*Hyphessobrycon takasei*	Coffee bean tetra
838.	*Hyphessobrycon vilmae*	Vilma's tetra
839.	*Hypopmus artedi*	Motted/spotted knife fish
840.	*Hypopmus brevirostris*	Blunt-headed knife fish
841.	*Hypopmus brevirostris*	Blunt-headed knife fish
842.	*Hypoptopoma gulare*	Flatnose dwarf sucker catfish
843.	*Hypostomus jaguribensis*	Rio jaguribe hypostomus
844.	*Hypostomus niceferoi*	Nicefero's hypostomus
845.	*Hypostomus plecostomus*	Plecostomus/Sucker catfish
846.	*Hypostomus punctatus*	Spotted hypostomus
847.	*Hypostomus* sp "diagonal bar"	Diagonal bar hypostomus
848.	*Hypostomus varimaculosus*	Variable spot hypostomus
849.	*Hypostomus watwata*	Watwata pleco
850.	*Hypseleotris compressus*	Empire goby
851.	*Hypseleotris cyrinoides*	Chameleon goby
852.	*Hypseleotris guentheri*	Chameleon sleeper
853.	*Hypseleotris klunzingeri*	Australian pink sleeper
854.	*Incuanodeltes spilurus*	Slender tetra
855.	*Iguanodectes tenius*	Splender barb
856.	*Ilyodon* sp "black band"	Black-banded goodeid
857.	*Indostomus paradoxus*	Paradox fish
858.	*Inpaichthys kerri*	Blue emperor cichlid
859.	*Iodotropheus* spp	
860.	*Iodotropheus sprengerae*	Rusty cichlid

861.	*Iriaterhrina werneri*	Featherfin/new guinea rainbow
862.	*Julidochromis dickfeldii*	Dickfeldi
863.	*Julidochromis marlieri*	Marlier's julie
864.	*Julidochromis ornatus*	Julie
865.	*Julidochromis regani*	Convict julie
866.	*Julidochromis* spp	
867.	*Julidochromis transcriptus*	Masked julie
868.	*Knodus breviceps*	Soap eater
869.	*Kryptopterus bicirrhis*	Glass catfish
870.	*Kryptopterus macrocephalus*	Poorman's glass catfish
871.	*Labeo albino*	Albino shark
872.	*Labeo bala-melanopterus*	Bala/silver shark
873.	*Labeo bicolor*	Red-tailed black shark
874.	*Labeo erythurus*	Red-fin shark
875.	*Labeo frenatus*	Rainbow shark
876.	*Labeo rohita*	Rohita
877.	*Labeochromis caeruleus = Labidochromis caeruleus*	Sky- blue labiolo
878.	*Labeochromis vellicans = Labidochromis vellicans*	Vellicans
879.	*Labeotropheus fuelleborni*	Fuelleborni cichlid
880.	*Labeotropheus* spp	
881.	*Labeotropheus travawasae = Labidochromis trevawasae*	Red-top trewavasae
882.	*Labidochromis exaxperatus = Melanochromis exasperatus*	Orange-lined cichlid
883.	*Labidochromis joanjohnsonae = Melanochromis joanjohnsonae*	Banner cichlid
884.	*Labidochromis* spp	
885.	*Labidochromis textilis*	Cloth-of-gold cichlid
886.	*Labiobarsus burmanicus*	Long-finned shark
887.	*Labiobarsus festivus*	Signal barb
888.	*Ladigesia roloffi*	Jely-bean tetra
889.	*Lamprichthys tanganicanus*	Tanganyika pearl killie
890.	*Lamprologus attenuatus*	Marbled lamprologus
891.	*Lamprologus brevis*	Brevis
892.	*Lamprologus brichardi = Nelamprologus elongatus*	Lyretail lamprologus
893.	*Lamprologus buscheri*	Striped lamprologus
894.	*Lamprologus callipterus*	Callipterus
895.	*Lamprologus calvus*	Pearly lamprologus
896.	*Lamprologus compressiceps*	Compressiceps
897.	*Lamprologus congolensis*	Congo lamprologus
898.	*Lamprologus cunningtoni*	Black lamprologus
899.	*Lamprologus elongatus*	Elongatus
900.	*Lamprologus fasciatus*	Barred lamprologus
901.	*Lamprologus furcifer*	
902.	*Lamprologus kendali = Leidiolamprilogus kendali*	Nkambe
903.	*Lamprologus leleupi*	Lemon cichlid
904.	*Lamprologus melas*	Dusky lemon cichlid
905.	*Lamprologus moerii*	Moer's lemprologus
906.	*Lamprologus pleuromaculatus*	Pleuromaculatus
907.	*Lamprologus savoryi elongatus*	Lyretail lamprologus
908.	*Lamprologus* sp "Margarae"	Margare/Magara shell dweller
909.	*Lamprologus* spp	
910.	*Lamprologus tretocephalus*	Five bar cichlid
911.	*Lamprologus werneri*	
912.	*Laubuca dadiburjori*	Dadio
913.	*Laubuca laubuca*	Indian hatchett fish
914.	*Lebiasina bimaculata*	Two-spotted lebiasina
915.	*Lebiasina Panamensis*	Panama pencilfish
916.	*Lebistes recticulatus = Poecilia reticulate*	Guppy/million's fish
917.	*Lefua costata*	Lefua
918.	*Leiarius pictus*	Salfin pimelodid
919.	*Leiocassis branshnikowi*	Russian catfish
920.	*Leiocassis poeciliopterus*	Bee cat
921.	*Leiocassis siamensis*	Barred siamese catfish
922.	*Lepidarchus adonis*	Adonis
923.	*Lepidocephalus goalparensis*	
924.	*Lepidocephalus guniea*	
925.	*Lepidocephalus thermalis*	Lesser loach
926.	*Lepidogalaxias salamandroides*	Long-finned galaxias
927.	*Leporellus vittatus*	Leporellus
928.	*Leporinus affinis*	
929.	*Leporinus agassizi*	Half-striped leporinus
930.	*Leporinus arcus*	Lipstick leporinus
931.	*Leporinus* cf *granti*	Red leporinus
932.	*Leporinus desmotes*	Black and yellow leporinus
933.	*Leporinus fasciatus*	Banded leprinus
934.	*Leporinus frederici*	Frederici

935.	*Leporinus maculatus*	Spotted leporinus
936.	*Leporinus megalepis*	Three-spot leporinus
937.	*Leporinus melanostictus*	Silver leporinus
938.	*Leporinus melanopleura*	Spot-tailed leporinus
939.	*Leporinus multifasciatus*	Multi banded leporinus
940.	*Leporinus nigrotaeniatus*	Black-lined leporinus
941.	*Leporinus ???ctofasciatus*	Eight-banded leporinus
942.	*Leporinus pearsoni*	Pearson’s leporellus
943.	*Leporinus pellegrini*	Spotted/pelegrins leporinus
944.	*Leporinus* sp “Golden”	Golden leporinus
945.	*Leporinus striatus*	Striped leporinus
946.	*Leptobarsus hoeveni*	Pink-tailed barb
947.	*Limia caudofasciata*	Steel blue lima/blue poecilia
948.	*Limia heterandria*	Dwarf/haiti lima
949.	*Limia melanogaster*	Black bellied/Blue lima
950.	*Limia nicholsi*	Nichol’s lima
951.	*Limia nirofasciata*	Humpback/Black barred lima
952.	*Limia ornata*	Ornate limia
953.	*Limia versicolor*	Olive limia
954.	*Limia vittata*	Cuban limia
955.	*Limnochromis auritus*	Auritus
956.	*Limnochromis* spp	
957.	*Limnotilapia* spp	
958.	*Liosomadoras oncinus*	Jaguar catfish
959.	*Liphiobagrus cyclurus*	Poison slime catfish
960.	*Loricaria filamentosa*	Whiptail catfish
961.	*Loricaria parva*	Alligator catfish
962.	*Luciania goodey*	Bluefin killfish
963.	*Luciocharax insculptus*	
964.	*Macrognathus aculeatus*	Spiny eel
965.	*Macrognathus siamensis*	Spot-finned spiny eel
966.	*Macropodus chinesis*	Round-tailed paradise fish
967.	*Macropodus concolor*	Black paradise fish
968.	*Macropodus cupanus cupanus*	Spike-tailed paradise fish
969.	*Macropodus capanus dayi*	Brown paradise fish/day’s fish
970.	*Macropodus opercularis*	Paradise fish/blue combtail
971.	*Macropodus opercularis concolor = M opercularis*	Black paradise fish
972.	*Malpulutta kretseri*	Malpulutta
973.	*Mastacembelus armatus*	White spotted spiny eel
974.	*Mastacembelus erythrotaenia*	Spotted fire eel
975.	*Mastacembelus pancalus*	Spiny eel
976.	*Mastacembelus paucispinnis*	Short finned spiny eel
977.	*Mastacembelus zebrinus*	Zebra spiny eel
978.	*Mastacembelus circumcinctus*	Half-banded spiny eel
979.	*Mastacembelus* sp “Smith”	False armatus spiny eel
980.	*Megalamphodus* sp “Rubra”	Red megalamphodus
981.	*Megalamphodus axelrodi*	Calypso tetra/Red pristella
982.	*Megalamphodus megalopterus*	Black phantom tetra
983.	*Megalamphodus sweglesi*	Red phantom tetra
984.	*Melanochromis auratus = Pseudotropheus auratus*	Malawi golden cichlid
985.	*Melanochromis chipokee*	Chipokee
986.	*Melanochromis crabro*	Chameleon cichlid
987.	*Melanochromis johanni*	Johanni
988.	*Melanochromis melanopterus*	Black mbuna
989.	*Melanochromis parallelus*	Parallel-striped mbuna
990.	*Melanochromis perspicax*	Violet-striped mbuna
991.	*Melanochromis simulans*	Longsnout mbuna
992.	*Melanochromis* spp	
993.	*Melanochromis vermivorus*	Purple mbuna
994.	*Melanotaenia affinis*	North new guinea rainbowfish
995.	*Melanotaenia boesemani*	Boesman’s rainbowfish
996.	*Melanotaenia coatesi*	Coates’s rainbowfish
997.	*Melanotaenia fluvuatilis*	Pink-ear rainbowfish
998.	*Melanotaenia goldei*	Goldie river rainbowfish
999.	*Melanotaenia herbertaxelrodi*	Lake tebera rainbowfish
1000.	*Melanotaenia lacustris*	Lake kutuba rainbowfish
1001.	*Melanotaenia maccullochi*	Dwarf australian rainbowfish
1002.	*Melanotaenia monticola*	Mountain rainbowfish
1003.	*Melanotaenia nigrans*	Dark australian rainbowfish
1004.	*Melanotaenia octediensis*	Oktedi rainbowfish
1005.	*Melanotaenia parkinsoni*	Parkinson’s rainbowfish
1006.	*Melanotaenia sexlineata*	Fly river rainbowfish
1007.	*Melanotaenia splendida*	Pink-tailed australian rainbowfish
1008.	*Melanotaenia trifasciata*	Banded rainbowfish
1009.	*Melanotaenia tigrinus*	Tiger striped catfish

1010.	*Metynnis calichromus*	
1011.	*Metynnis hypsauchen*	Plain metynnis/Silver dollar
1012.	*Metynnis luna*	Moon metynnis
1013.	*Metynnis maculatus*	Spotted metynnis/Silver dollar
1014.	*Metynnis roosevelti*	Silver dollar
1015.	*Metynnis schreitmuelleri*	Schreitmueller's metynnis
1016.	*Migralestes interruptus*	Congo tetra
1017.	*Migralestes stormsi*	Blue-barred congo tetra
1018.	*Mycrobrycon fredcochui*	Cochui's blue tetra
1019.	*Microgeophagus ramirezi – apistogramma ramirezi*	Ram/butterfly cichlid
1020.	*Microglanis poecilus*	Dwarf marbled catfish
1021.	*Micropanchax macrophthalus*	Lapeye panchax
1022.	*Microphis smithi*	Large freshwater pipefish
1023.	*Micropoecillia branneri*	Branner's liverbearer
1024.	*Micropoecillia malanozorus*	Blue guppy
1025.	*Mimagoniates barberi*	Blue tetra/barber's tetra
1026.	*Mimagoniates inaequalis*	Croaking tetra
1027.	*Mimagoniates microlepis*	Blue tetra
1028.	*Miroglanis paraphybae*	Bumble bee catfish
1029.	*Moenkhausia agnesae*	Agnes' tetra
1030.	*Moenkhausia collettii*	Collett's tetra
1031.	*Moenkhausia copei*	Cope's moenkhausia
1032.	*Moenkhausia dichroupa*	Spot tailed moenhaustia
1033.	*Moenkhausia lepidura*	Half-mast flag tetra
1034.	*Moenkhausia oligolepsis*	Glass tetra
1035.	*Moenkhausia pittieri*	Diamond tetra
1036.	*Moenkhausia robertsi*	Iquitos moenkhausia
1037.	*Moenkhausia sanctae-filomenae*	Red-eye tetra
1038.	*Moenkhausia takasei*	Takase's moenkhausia
1039.	*Mollienesia caucana*	South american molly
1040.	*Mollienesia dominocensis*	Domingo molly
1041.	*Mollienesia latipinna*	Green sailfin molly
1042.	*Mollienesia sphenops*	Black marble molly
1043.	*Mollienesia velifera*	Salfin molly
1044.	*Monocirrhus polycanthus*	South american leaf fish/leather fish
1045.	*Monodactylus sebae = Psettus sebae*	Finger fish
1046.	*Morgurnda morgurnda*	Persian carpet/sleepy trout
1047.	*Mormyrops nigricans*	
1048.	*Morulius chrysophekaidon*	Black shark
1049.	*Myloplus asterias*	Starry myloplus
1050.	*Myloplus schultzei*	Schultzei myloplus
1051.	*Mylossoma argenteum*	Silver mylossoma
1052.	*Mylossoma aureum*	Silver dollar
1053.	*Mylossoma duriventres*	Hard-bellied characin
1054.	*Mylossoma parguayensis*	Paraguayan metynnis
1055.	*Mystus bleekeri*	
1056.	*Mystus keletius*	Dwarf catfish
1057.	*Mystus tengara*	Pearl catfish
1058.	*Mystus vittatus*	Striped catfish
1059.	*Nandus nandus*	Nandus
1060.	*Nannacara anomala*	Golden-eyed dwarf cichlid
1061.	*Nannacara aureocephalus*	Golden-head dwarf cichlid
1062.	*Nannacara taenia*	Lattice dwarf cichlid
1063.	*Nannaethiops tritaeniatus*	Three-line african characin
1064.	*Nannaethiops unitaeniatus*	One-lined african characin
1065.	*Nannaethiops unitaeniatus*	One-lined african tetra
1066.	*Nannabrycon eques*	Tube-moutherd pencil fish
1067.	*Nannochrax fasciatus*	African characidium
1068.	*Nannostomus anomalis*	Anomalus pencil fish
1069.	*Nannostomus atripirangensis*	Aripara/brown pencil fish
1070.	*Nannostomus beckfordi*	Golden/beckford's pencil fish
1071.	*Nannostomus bifasciatus*	Two-banded pencil fish
1072.	*Nannostomus digrammus*	Twin-stripe pencil fish
1073.	*Nannostomus eques*	Brown-tailed pencil fish
1074.	*Nannostomus espei*	Barred pencil fish
1075.	*Nannostomus harrisonii*	Harrison's pencil fish
1076.	*Nannostomus marginatus*	Dwarf pencil fish
1077.	*Nannostomus trifasciatus*	Three-lined pencil fish
1078.	*Nannostomus unifasciatus*	One-lined pencil fish
1079.	*Nanochromis dimidiatus*	Dimidiatus
1080.	*Nannochromis nudiceps*	Blue dwarf/nudiceps cichlid
1081.	*Nannochromis paralius*	Dwarf cichlid
1082.	*Nanochromis splendens*	Congo dwarf cichlid
1083.	*Neetroplus nematopus*	Little lake cichlid
1084.	*Nemacheilus kessleri*	Kessler's loach

1085.	*Nematobrycon amphiloxus*	Rainbow tetra
1086.	*Nematobrycon lacortei*	Rainbow tetra
1087.	*Nematobrycon palmeri*	Emperor tetra
1088.	*Nematobrycon* sp "gery"	Gery's emperor tetra
1089.	*Neolebias ansorgi*	Ansorge's neolebias
1090.	*Neolebias landgrafi*	Blue banded neolebias
1091.	*Neolebias trewavasae*	Trewava's neolebias
1092.	*Neolebias trilineatus*	Three-lined neolebias
1093.	*Nerophis opaidon*	Straight-nosed pipefish
1094.	*Nomorhamphus celebensis*	Celebes half-break
1095.	*Nomorhamphus liemi*	Black-finned celebes halfbeak
1096.	*Nothobranchius foerschi = Nothobranchius palmouisii*	
1097.	*Nothobranchius furzeri*	Turquoise killerfish
1098.	*Nothobranchius guentheri*	Gunter's nothobranch
1099.	*Nothobranchius janpappi*	Topwater fire killie
1100.	*Nothobranchius jubbi*	Blue notho
1101.	*Nothobranchius korthausae*	Kothaus's killie
1102.	*Nothobranchius lourensi*	Green notho
1103.	*Nothobranchius mayeri*	Meyer's nothobranch
1104.	*Nothobranchius melanospilus*	Beira nothobranch
1105.	*Nothobranchius palmouisti = Nothobranchius foerschi*	Palmouists's nothobranch
1106.	*Nothobranchius patrizii*	Red-tailed turquoise notho/patrizis not
1107.	*Notopterus chitala*	Clown knifefish
1108.	*Notopterus mikereedi*	Clown knifefish
1109.	*Ocellatus astronatus*	Oscar
1110.	*Oligolepsis acutipennis*	Sharp tail goby
1111.	*Ophthalmochromis* spp	
1112.	*Ophthalmochromis ventralis*	Yellow-tipped ventralis
1113.	*Ophthalmochromis nasutus*	Long-nosed gold-tip cichlid
1114.	*Ophthalmotilapia* spp	
1115.	*Ophthalmotilapia ventralis*	Blue cold-tip cichlid
1116.	*Osphronemus couramy*	Giant courami
1117.	*Osteochilus ferreirai*	Black arowana
1118.	*Osteochilus hasselti*	Hard-lipped barb
1119.	*Osteochilus vittatus*	Bony-lipped barb
1120.	*Osteoglossum bicirrhosum*	Arowna
1121.	*Osteoglossum ferreirai*	Black arowana
1122.	*Otocinclus affinis*	Midget sucker catfish
1123.	*Otocinclus arnoldi*	Alcae eater/sucker catfish
1124.	*Otocinclus flexilis*	Imitator sucker catfish
1125.	*Otocinclus mariae*	Maria's otocinclus
1126.	*Otocinclus nattereri*	Natterer's otocinclus
1127.	*Otocinclus nigricauda*	Black-tail sucker catfish
1128.	*Otocinclus vittatus*	Striped sucker catfish
1129.	*Oxyeleotris marmorata*	Marbled sleeper coby
1130.	*Pachypanchax homalonotus*	Powder-blue panchax/green panchax
1131.	*Pachypanchax playfairi*	Golden panchax
1132.	*Panaque nigrolineatus*	Panaque catfish
1133.	*Panaque suttoni*	Blue-eyed panaque
1134.	*Pangasius pangasius*	
1135.	*Pangasius sutchi*	Pangasius cat/bkye catfusg/suanese sha ??
1136.	*Pantodon buchholzi*	Butterfly fish
1137.	*Paracheirodon axelrodi*	Cardinsal tetra
1138.	*Paracheirodon innesi*	Neon tetra
1139.	*Paracheirodon simulans*	False neon
1140.	*Parailia* spp	Glass knifefish
1141.	*Parailia congica*	Congo African glass catfish
1142.	*Parailia longifilis*	Mottled glass catfish
1143.	*Parailia occidentalis*	West African glass catfish
1144.	*Parapocryptes serperaster*	Slim mudskipper
1145.	*Parapristella georgiae*	Plain jane
1146.	*Parauchenipterus galeatus*	Starry cat
1147.	*Parauchenoglanis guttatus*	African flathead catfish
1148.	*Parauchenoglanis macrostoma*	African spotted catfish
1149.	*Parodon affinis*	Paraguay darter tetra
1150.	*Parodon caliensis*	Barred dartertetra
1151.	*Parodon piracicabae*	Brazilian darter tetra
1152.	*Parodon pongoense*	Pongo pongo
1153.	*Parosphromenus nagyi*	Nagy's licorice gourami
1154.	*Parosphromenus paludicola*	Pallid licorice gourami
1155.	*Parosphromenus parvulus*	Pycmy licorice gourami
1156.	*Parosphromenus deissneri*	Licorice gourami
1157.	*Parosphromenus filamentosus*	Spike-tailed licorice gourami
1158.	*Parotocinclus maculicauda*	Redfin otocinclus
1159.	*Peckoltia brevis*	Spotted head peckoltia

1160.	*Peckoltia pulcher*	Pretty peckoltia
1161.	*Peckoltia vittata*	Banded peckolita
1162.	*Pedalibrycon felipponei*	Uruguay characin
1163.	*Pelmatochromis arnoldi*	Arnold's cichlid
1164.	*Pelmatochromis guentheri*	Guenther's mouthbrooder
1165.	*Pelmatochromis humilis*	Kasewe krib
1166.	*Pelmatochromis klugei*	Kluge's dwarf cichlid
1167.	*Pelmatochromis kribensis*	Kribensis/purple cichlid
1168.	*Pelmatochromis pulcher = Pelvicachromis pulcher*	Kribensis
1169.	*Pelmatochromis subocellatus*	Violet cichlid
1170.	*Pelmatochromis taeniatus*	Striped African dwarf cichlid
1171.	*Pelmatochromis thomasi = Hemichromis thomasi*	Butterfly/Thomas's cichlid
1172.	*Pelmatochromis pulcher = Pelmatochromis pulcher*	Kribensis
1173.	*Pelmatochromis roloffi*	Roloff's kribensis
1174.	*Pelvicachromis subocellatus*	Ocellated kribensis
1175.	*Pelmatochromis taeniatus*	Striped kribensis
1176.	*Periophthalmus barbarus*	Mudskipper
1177.	*Periophthalmus papilo*	Butterfly mudskipper
1178.	*Periophthalmus vulgaris*	Flagfin mudskipper
1179.	*Perrunichthys perruno*	Reticulated pimelodid/ perrunc/leopard catfish
1180.	*Petersius occidentalis*	
1181.	*Petitella georgiae*	False rummynose
1182.	*Petrotilapia nigra*	Dusky petrotilapia
1183.	*Petrotilapia tridentiger*	Blue petrotilapia
1184.	*Phallichthys amates*	Merry widow
1185.	*Phallichthys pittieri*	Orange dorsal-fin livebearer
1186.	*Phenacogaster pectinatus*	Pectinatus
1187.	*Phenacogrammus caudomaculatus*	Moon tetra
1188.	*Phenacogrammus deheyni*	Deheyni's congo tetra
1189.	*Phenacogrammus interruptus*	Congo tetra
1190.	*Phenacogrammus* sp "Diamond Congo"	Diamond Congo tetra
1191.	*Phenaconiates macrolepis*	Barred glass tetra
1192.	*Phoxinopsis typicus*	Black-line tetra
1193.	*Phractocephalus hemiliopterus*	Red-tailed catfish
1194.	*Phractolaemus ansorgei*	African mudfish
1195.	*Phractura ansorgei*	African whiptailed catfish
1196.	*Phyllonemus typus*	Spatula-barbeled catfish
1197.	*Physailia pellucida*	African glass fish
1198.	*Piabucus caudomaculatus*	Spot-tailed slender tetra
1199.	*Piabucus dentatus*	Golden-striped slender tetra
1200.	*Pimelodella clarias*	Spotted pimelodella
1201.	*Pimelodella dorseyi*	Dorsey's pimelodella
1202.	*Pimelodella lateristrica*	
1203.	*Pimelodella linami*	Linam's pimelodella
1204.	*Pimelodella metae*	Pimelodella
1205.	*Pimelodella parnahybae*	Parnahyba pimelodella
1206.	*Pimelodella pictus*	Angelicus catfish
1207.	*Pimelodus albofasciatus*	White-striped pimelodid
1208.	*Pimelodus blochi*	Dusky pimelodid
1209.	*Pimelodus mucalatus*	Spotted pimelodid
1210.	*Pimelodus ornatus*	Ornate pimelodus
1211.	*Pimelodus pictus*	Angelicus pimelodus
1212.	*Pimelodus megalopterus*	Pictus
1213.	*Pinirampus piranampu*	Long-finned catfish
1214.	*Platydoras costatus*	White candy striped American catfish
1215.	*Platystacus cotylephorus*	Mottled whiptailed banjo catfish
1216.	*Platystomatichthys sturio*	Sturgeon catfish
1217.	*Platytropius siamensis*	False siamese shark
1218.	*Plecostomus bolivianus*	Bolivian sucker catfish
1219.	*Plecostomus hypostomus*	Sucker catfish
1220.	*Plecostomus plecostomus*	Pluto/sucker catfish
1221.	*Plecostomus punctatus*	Plecostomos/sucker catfish
1222.	*Poecilia caucana*	Cauca molly
1223.	*Poecilia dominicensis*	Dominican limia
1224.	*Poecilia latipinna*	Sailfin molly
1225.	*Poecilia melanogaster*	Black-bellied limia
1226.	*Poecilia Mexicana*	Mixican molly/shortfin molly
1227.	*Poecilia Nigrofasciata*	Humpbacked limia
1228.	*Poecilia ornata*	Ornate limia
1229.	*Poecilia parae*	Two-spot livebearer
1230.	*Poecilia reticulata = Lebistes reticulatus*	Guppy/million's fish
1231.	*Poecilia sphenops*	Sphenops molly/molly
1232.	*Poecilia velifera*	Sailfin molly
1233.	*Poecilia vittata*	Molly/banded lima
1234.	*Poecilia vivipara*	One-spot livebearer

1235.	*Poeciliopsis gracilis*	Porthole livebearer
1236.	*Poecilistes pleurospilus*	Port hole fish/port hole live-bearer
1237.	*Poecilobrycon digrammus*	Two-striped pencil fish
1238.	*Poecilobrycon eques*	Pencil/black-tailed pencil fish
1239.	*Poecilobrycon espei*	Barred pencil fish
1240.	*Poecilobrycon unifasciatus*	Pencil fish
1241.	*Poecilocharax weitzmani*	Black darter tetra
1242.	*Polycentropsis abbreviata*	African leaf fish
1243.	*Polycentrus schomburgki*	South American leaf fish/Schomburgki
1244.	*Polypterus bichir*	Nile bichir
1245.	*Polypterus enlicheri*	Serpent head
1246.	*Polypterus ornatipinnis*	Ornate bichir
1247.	*Polypterus palmas*	Polypterus palmas/marbled bichir
1248.	*Polypterus retrapinnis*	Marbled bichir
1249.	*Polypterus* spp	Bichir
1250.	*Popondetta connieae*	Popondetta rainbowfish
1251.	*Popondetta furcata*	Forktail fainbowfish
1252.	*Poptela orbicularis*	Silver disc tetra
1253.	*Potamorraphis guianensis*	Freshwater needle fish
1254.	*Potamorhina latior*	Silver curimata
1255.	*Potamorhina squamoralevis*	Big-tail curimata
1256.	*Priapella intermedia*	Blue-eyed livebearer
1257.	*Priapichthys chocoensis*	Colombian diamond-scale
1258.	*Prionobrama filigera*	Glass bloodfin tetra
1259.	*Prionobrama paraguayensis*	Southern bloodfin
1260.	*Pristella maxillaris*	Pristella
1261.	*Pristella riddlei*	X-Ray fish/watergold finch
1262.	*Pristigaster cayana*	Amazonian hatchet herring
1263.	*Procatopus aberrans*	Grren lampeye
1264.	*Procatopus gracilis*	Powder-blue lampeye
1265.	*Procatopus nototaenia*	Blue-lady minnow
1266.	*Procatopus similis*	Nigerian lampeye
1267.	*Procatopus* sp “fire tail”	Firetail lampeye
1268.	*Prochilodus insignius*	Prochilodus/insignius tetra
1269.	*Prochilodus nigricans*	Black prochilodus/spotted smallmouth
1270.	*Prochilodus ortonianus*	Gray prochilodus
1271.	*Prochilodus taeniurus*	Silver prochilodus
1272.	*Psettus sebae = Monodactylus sebae*	Finger fish
1273.	*Pseudacanthicus leopardus*	Orange trim sucker catfish
1274.	*Pseudauchenipterus nodosus*	Black tailband catfish
1275.	*Pseudepapterus hasemani*	Haseman’s woodcat
1276.	*Pseudobagrus fulvidraco*	Tawny dragon catfish
1277.	*Pseudochaleaus kyburzi*	Kyburz tetra
1278.	*Pseudocorynopoma doriae*	Dragon-finned characin
1279.	*Pseudodoras niger*	Black doradid
1280.	*Pseudogastromyzon myersi*	Myer’s hillstream loach
1281.	*Pseudomugil gertrudae*	Gertrude’s blue eye
1282.	*Pseudomugil inconspicuus*	Inconspicuous blue-eye
1283.	*Pseudomugil paludicola*	Swamp blue-eye
1284.	*Pseudomugil signifer*	Southern blue eye
1285.	*Pseudomugil tenellus*	Delicate blue eye
1286.	*Pseudopimelodus albomarcinatus*	Black-banded pimelodid
1287.	*Pseudopimelodus nigricauda*	Mottled pseudopimelodid
1288.	*Pseudopimelodus transmontanus*	Peruvian mottled catfish
1289.	*Pseudoplatystoma fasciatum*	Tiger catfish
1290.	*Pseudosphromenus cupanus cupanus*	Spiketail paradise fish
1291.	*Pseudotropheus auratus = Melanochromis auratus*	Auratus/lake Nyassa characin
1292.	*Pseudotropheus brevis*	Silver-blue mbuna
1293.	*Pseudotropheus crabro*	
1294.	*Pseudotropheus elongatus*	Slender mbuna
1295.	*Pseudotropheus lanisticola*	Slender mbuna/snail shell mbuna
1296.	*Pseudotropheus livingstoni*	Snail shell mbuna
1297.	*Pseudotropheus lombardoi*	Kennyi
1298.	*Pseudotropheus microstoma*	Small-mouth tropheops
1299.	*Pseudotropheus novemfasciatus*	Novemfasciatus
1300.	*Pseudotropheus ornatus*	Bumble-bee characin
1301.	*Pseudotropheus* sp “dusky lavender cichlid”	Dusky lavender cichlid
1302.	*Pseudotropheus* sp “dusky”	Dusky mbuna
1303.	*Pseudotropheus* sp “eduardi”	Eduardi
1304.	*Pseudotropheus* sp “orange”	Orange mbuna
1305.	*Pseudotropheus* spp	
1306.	*Pseudotropheus tropheops*	Mblina/tropheopos
1307.	*Pseudotropheus williamsi*	William’s mbuna
1308.	*Pseudotropheus zebra*	Zebra characin/Nyassa blue cichlid
1309.	*Pseudoxiphophorus bimaculatus*	False swordtail

1310.	*Pterodiscus levis*	Silver hatchett fish
1311.	*Pterophyllum altum*	Deep angel fish
1312.	*Pterophyllum dumerili*	Long-nose angel fish
1313.	*Pterophyllum eimekei*	Lesser angel fish
1314.	*Pterophyllum scalare*	Angel fish
1315.	*Pterycoplichthys anisitsi*	Snow-king sucker catfish
1316.	*Pterycoplichthys bolivianus*	Bolivian sucker catfish
1317.	*Pterycoplichthys gibbiceps*	Sailfin sucker catfish
1318.	*Pterolebias longipinnis*	Peruvian longfin
1319.	*Pterolebias peruensis*	Peruvian longfin
1320.	*Pterolebias wischmanni*	Wischmann's pterolebias
1321.	*Pterolebias zonatus*	Lace finned killie
1322.	*Puntiaplicus practozyoron*	Yellow chequered barb
1323.	*Puntius bimaculatus*	Two spot barb
1324.	*Puntius conchonius*	Rosy barb
1325.	*Puntius cummingi*	Cumming's barb
1326.	*Puntius everetti*	Clown barb
1327.	*Puntius filamentosus*	Filament/black spot barb
1328.	*Puntius celius*	Dwarf/miniature barb
1329.	*Puntius lateristiga*	Spanner barb
1330.	*Puntius lineatus*	Striped barb
1331.	*Puntius nigrofasciatus*	Black ruby barb
1332.	*Puntius oligolepis*	Cgequered barb ??
1333.	*Puntius pentazona*	Fuve babded barb
1334.	*Puntius phutunio*	Dwarf barb
1335.	*Puntius sachsi*	Cold barb
1336.	*Puntius semifasciolatus*	Half banded barb
1337.	*Puntius sonphongsi*	Sonphong's barb
1338.	*Puntius stigma*	Two-spot barb
1339.	*Puntius stigmatopygus*	Mid-spot barb
1340.	*Puntius stoliczkai*	Stoliczka's barb
1341.	*Puntius sylvaticus*	Sylvan barb
1342.	*Puntius tetrazona*	Tiger barb
1343.	*Puntius ticto*	Ticto/tic-tac-toe barb
1344.	*Puntius vittatus*	Banded barb
1345.	*Puntius wohlerti*	Sickle barb
1346.	*Pyrrhulina brevis*	Short pyrrhulina
1347.	*Pyrrhulina laeta*	Half banded pyrrhulina
1348.	*Pyrrhulina nattersi*	Natter's pyrrhulina
1349.	*Pyrrhulina nigrofasciata*	Black banded pyrrhulina
1350.	*Pyrrhulina rachoviana*	Rachow's pyrrhulina/fanning characin
1351.	*Pyrrhulina* sp "red spotted"	Red spotted pyrrhulina
1352.	*Pyrrhulina* sp "short-lined No 2"	Short-lined pyrrhulina No 2
1353.	*Pyrrhulina spicota*	
1354.	*Pyrrhulina* spp	
1355.	*Pyrrhulina vittata*	Striped/banded pyrrhulina
1356.	*Quintana …..isona ??*	Black-barred livebearer
1357.	*Rachovia brevis*	Magdalena spot-finned killie
1358.	*Rachovia hummelincki*	Coastal spot-finned killie
1359.	*Rachovia maculipinnis*	Venezuelan spot-finned killie
1360.	*Rachovia pyropunctata*	Red-spotted spot-finned killie
1361.	*Raiamas ansorghi*	Black-barred barilius
1362.	*Rasbora agilis*	Black striped rasbora
1363.	*Rasbora argyrotaenia*	Silver rasbora
1364.	*Rasbora altior*	Green false rasbora
1365.	*Rasbora bankanensis*	Banka rasbora
1366.	*Rasbora borapetens*	Red tailed rasbora
1367.	*Rasbora brittani*	Brittan's rasbora
1368.	*Rasbora caudimaculata*	Greater scissor tail rasbora
1369.	*Rasbora cephalotaenia*	Port hole rasbora
1370.	*Rasbora chrysotaenia*	Gold striped rasbora
1371.	*Rasbora daniconius*	Slender rasbora
1372.	*Rasbora dorsiocellata*	Eye-spot/hi-spot rasbora
1373.	*Rasbora dusonensis*	Yellowtail rasbora
1374.	*Rasbora einthoveni*	Brilliant rasbora
1375.	*Rasbora elegans*	Two-spot/elegant rasbora
1376.	*Rasbora hengeli*	Red rasbora
1377.	*Rasbora heteromorpha*	Harlequin fish
1378.	*Rasbora jacobsoni*	Jacobson's rasbora
1379.	*Rasbora kalochroma*	Big spot rasbora
1380.	*Rasbora kobonensis*	
1381.	*Rasbora kobonensis*	Copper striped rasbora
1382.	*Rasbora laptosoma*	Yellow/elegant rasbora
1383.	*Rasbora maculate*	Spotted/pygmy rasbora
1384.	*Rasbora meinkeni*	Meinken's rasbora

1385.	*Rasbora myersi*	Meyer's rasbora
1386.	*Rasbora pauciperforata*	Red lined/glowlight rasbora
1387.	*Rasbora phillippina*	Phillippine rasbora
1388.	*Rasbora rasbora*	Rasbora
1389.	*Rasbora somphangsi*	Samphang's rasbora
1390.	*Rasbora sarawakensis*	Sarawak's rasbora
1391.	*Rasbora steineri*	Chinese rasbora
1392.	*Rasbora sumatrana*	Sumatran rasbora
1393.	*Rasbora taeniata*	Black-striped rasbora
1394.	*Rasbora tornieri*	Yellow tailed rasbora
1395.	*Rasbora trilineata*	Three-lined/scissor-tail rasbora
1396.	*Rasbora urophthalma*	Miniature/dwarf/ocellated/point rasbora
1397.	*Rasbora vaterifloris*	Ceylon fire barb/fire rasbora
1398.	*Rasborichthys altior*	Green rasbora
1399.	*Reganochromis calliurus*	Calliurus
1400.	*Rhabdolichops troscheli*	Short-headed knife fish
1401.	*Rhadinocentrus ornatus*	Southern soft-spined rainbow fish
1402.	*Rhinomugil corsula*	
1403.	*Rineloricaria castroi*	Castro's whiptail catfish
1404.	*Rineloricaria fallax*	
1405.	*Rineloricaria filamentosa = Dasyloricardia filamentosa*	
1406.	*Rineloricaria hasemani*	Haseman's whiptail catfish
1407.	*Rineloricaria lanceolagta*	Lanceolate whiptail catfish
1408.	*Rineloricaria microlepidogaster*	Small-scaled whiptail catfish
1409.	*Rineloricaria parva*	
1410.	*Rita rita*	
1411.	*Rivulus agilae*	Agila rivulus
1412.	*Rivulus amphoreus*	Guianas rivulus
1413.	*Rivulus atratus*	Butterfly rivulus
1414.	*Rivulus beniensis*	Reticulated rivulus
1415.	*Rivulus compressus*	Blue rivulus
1416.	*Rivulus cryptocallus*	Iridescent rivulus
1417.	*Rivulus c….indraceus ???*	Cuban/green brown rivulus
1418.	*Rivulus dorni*	Dorn's rivulus
1419.	*Rivulus harti*	Hart's rivulus
1420.	*Rivulus holmiae*	Golden-tailed rivulus
1421.	*Rivulus limoncochae*	Rio napo rivulus
1422.	*Rivulus magdalenae*	Bar-tailed rivulus/Magdalena rivulus
1423.	*Rivulus ocellatus*	Ocellated rivulus
1424.	*Rivulus peruanus*	Perimparoo rivulus
1425.	*Rivulus punctatus*	Spotted rivulus
1426.	*Rivulus roloffi*	Rolff's rivulus
1427.	*Rivulus santensis*	Santo's rivulus
1428.	*Rivulus strigatus*	Herringbone rivulus
1429.	*Rivulus urophthalmus*	Golden/green rivulus
1430.	*Rivulus xanthonotus*	Yellowback rivulus
1431.	*Rivulus xiphidius*	Band-tailed rivulus
1432.	*Roedoibes gautemalensis*	Guatemala glass characin
1433.	*Roedoibes microlepis*	Small scaled glass characin
1434.	*Rohtee alfrediana*	Copper minnow
1435.	*Roloffia chayteri*	Chayter's killie
1436.	*Sartor respectus*	Peculiar-mouthed anastomid
1437.	*Scatophagus argus*	Scat/argus/spotted catfish
1438.	*Scatophagus argus var rubrifrons*	Tiger scat
1439.	*Scatophagus tetracanthus*	African scat/African banded cat
1440.	*Schizodon fasciatum*	Four-barred leporinus
1441.	*Schultzites axelrodi*	Axelrod's moenkhausia
1442.	*Sciades marmoratus*	Marbled catfish
1443.	*Sciades pictus*	Painted fish
1444.	*Scortum barcoo*	Barcoo grunter
1445.	*Selenotoca multifasciata*	False scat
1446.	*Semaprochilodus squamilentus*	Sailfinned prochilodus
1447.	*Semaprochilodus taeniurus*	Silver prochilodus
1448.	*Semaprochilodus theraponura*	Flag tailed prochilodus
1449.	*Sicyopterus jonklaasi = Gobius jonklaasi*	Mountain coby
1450.	*Simochromis curvifrons*	Thick-headed cichlid
1451.	*Simochromis diagramma*	Diagramma
1452.	*Simochromis* spp	
1453.	*Simpsonichthys boitonei*	Brazilian lyrefin
1454.	*Sorubim lima*	Shovelnose catfish
1455.	*Spathodus erythrodon*	Blue spotted coby cichlid
1456.	*Spathodus spp*	
1457.	*Sphaerichrhys selatanansis*	Thin-barred chocolate gourami
1458.	*Sphaerichrhys acrostoma*	Black-tailed chocolate gourami
1459.	*Sphaerichrhys osphromenoides*	Chocolate gourami

1460.	*Steatocranus casuarius*	Lionhead/African blockhead cichlid
1461.	*Steatocranus mpozoensis*	Mpozo lionhead cichlid
1462.	*Steatocranus tinanti*	Slender lionhead cichlid
1463.	*Steatogenys duidae*	Barred knife fish
1464.	*Steatogenys elegans*	Mottled knife fish
1465.	*Sternarchorhamphus muelleri*	Mueller's knife fish
1466.	*Sternarchorhamphus oxyrhynchus*	Elephant-nose knifefish
1467.	*Sternarchus albifrons*	Black ghost
1468.	*Sternopygus macrurus*	Bumpy-back silver dollar
1469.	*Stigmatogobius sadanundio = Gobius sadanundio*	Knight coby
1470.	*Stoneiella leopardus*	Orange-trim sucker catfish
1471.	*? (indistinct - to be added)*	Whiptailed catfish/royal farowella
1472.	*Symphysodon aequifasciatus*	Pompadour/discus
1473.	*Symphysodon aequifasciatus aequifasciatus*	Green discus
1474.	*Symphysodon aequifasciatus axelrodi*	Brown discus
1475.	*Symphysodon aequifasciatus haraldi*	Blue discus
1476.	*Symphysodon discus discus*	Red discus/pompadour
1477.	*Symphysodon discus willischwartzi*	Pineapple discus
1478.	*Symphysodon cingulatus*	Funny-mouthed anostomid
1479.	*Syngnathus pulchellus*	African freshwater pipefish
1480.	*Syngnathus spicifer = Hippichthys spicifer*	Belly-barbed pipefish
1481.	*Synodontis acanthomias*	Black-spotted synodontis
1482.	*Synodontis angelicus*	Polka-dot African catfish
1483.	*Synodontis brichardi*	Brichard's catfish
1484.	*Synodontis budgetti*	Brown synodontis
1485.	*Synodontis congicus*	Congo synodontis
1486.	*Synodontis contraqctus*	African upside-down catfish
1487.	*Synodontis davidi*	David's upside-down catfish
1488.	*Synodontis decorus*	Upside-down catfish
1489.	*Synodontis flavitaeniatus*	Striped synodontis
1490.	*Synodontis greshoffi*	Greshoff's synodontis
1491.	*Synodontis haugi*	Black synodontis
1492.	*Synodontis longifrostris*	Long-nosed synodontis
1493.	*Synodontis multipunctatus*	Spotted catfish
1494.	*Synodontis nigriventris*	Black-spotted catfishbellied upside-down catfish
1495.	*Synodontis notatus*	Spotted synodontis
1496.	*Synodontis ornatipinnis*	Bar-finned upside-down catfish
1497.	*Synodontis petricola*	Even-spotted synodontis
1498.	*Synodontis pleurops*	Big-eyed upside-down catfish
1499.	*Synodontis polli*	Poll's upside down catfish
1500.	*Synodontis robbianus*	Spotfin synodontis
1501.	*Synodontis robertsi*	Large-blotched synodontis
1502.	*Synodontis schoutedeni*	Vermiculated synodontis
1503.	*Taeniacara candidi*	Black-striped dwarf cichlid
1504.	*Tanakia tanago*	Miyakotanago
1505.	*Tanichthys albonubes*	White cloud mountain
1506.	*Tatia aulopygia*	Snowflake woodcast
1507.	*Tatia perugiae*	Perugia's woodcat
1508.	*Teleogramma brichardi*	Brichard's African dwarf cichlid
1509.	*Telestes agassizi*	Stromer
1510.	*Telmatherina ladigesi*	Glass neon/Celebes rainbow fish
1511.	*Telmatochromis bifrenatus*	Stripped telmat
1512.	*Telmatochromis caninus*	Caninus
1513.	*Telmatochromis* spp	
1514.	*Telmatochromis temporalis*	Temporalis
1515.	*Telmatochromis vittatus*	Bluntheaded telmat
1516.	*Tetragonopterus argenteus*	Silver tetra
1517.	*Tetragonopterus chalceus*	False silver tetra
1518.	*Tetraodon cutcutia*	Common/Malayan puffer fish
1519.	*Tetraodon duboisi*	Stanley pool puffer
1520.	*Tetraodon erythrotaenia*	Red-lined puffer fish
1521.	*Tetraodon fluviatilis*	Green/spotted puffer fish
1522.	*Tetraodon leiurus*	Twin-spot puffer
1523.	*Tetraodon palembangensis*	Figure eight puffer fish
1524.	*Tetraodon schoutedeni*	Puffer fish
1525.	*Thayeria oblique*	Short-striped penguin
1526.	*Thayeria boehlkei*	Short-striped penguin fish
1527.	*Thayeria sancgtaemariae*	Penguin fish
1528.	*Thoracocharax maculatus*	Spotted hatchet fish
1529.	*Thoracocharax seguris*	Long-fin hatchet fish
1530.	*Thoracocharax stellatus*	Deep-bodied silver hatchet fish
1531.	*Thysia ansingi*	Five-spot African cichlid
1532.	*Toxotes chatareus*	Seven-spot archer fish
1533.	*Toxotes jaculator*	Archer fish
1534.	*Trachelyopterichthys taenatius*	Striped woodcat

1535.	*Trachycorystes trachycorystes*	Indistinguished woodcat
1536.	*Trachydoras paraguayensis*	Paraguay doradid
1537.	*Trematocara* spp	
1538.	*Trematocranus jacobfreibergi*	Jacobfreibergi
1539.	*Trematocranus pterdaviesti*	Banded trematocranus
1540.	*Trematocranus* spp	
1541.	*Triacanthus brevirostris*	Tripod fish
1542.	*Trichogaster leeri*	Pearl/mosaic/large gourami
1543.	*Trichogaster marmoratus*	Alpine gourami
1544.	*Trichogaster microlepis*	Moonlight/silver gourami
1545.	*Trichogaster pectoralis*	Snakeskin gourami
1546.	*Trichogaster sumatrans* "cosby"	Cosby gourami
1547.	*Trichogaster trichopterus*	Gold gourami
1548.	*Trichogaster trichopterus sumatrans*	Blue gourami
1549.	*Trichopsis pumilis*	Pygmy/dwarf/sparkling gourami
1550.	*Trichopsis schalleri*	Three-striped croaking gourami
1551.	*Trichopsis vittatus*	Croaking gourami
1552.	*Trichonectes balzanii*	Rivulichthys
1553.	*Triconectes strigabundus*	Brazilian false false panchaz
1554.	*Triportheus albus*	Schalcinus
1555.	*Triportheus angulatus*	Narrow hatchef fish
1556.	*Triportheus rotundatus*	Black-winged triportheus
1557.	*Tropheus brichardi*	Blue-eyed tropheus
1558.	*Tropheus duboisi*	Duboisi
1559.	*Tropheus moorii*	Malawi cichlid/moorii
1560.	*Tropheus moorii kasabae*	Flat tropheus
1561.	*Tropheus* sp "Mpulunga"	Mpulunga tropheus
1562.	*Tropheus spp*	
1563.	*Trylochromis lateralis*	Congo tylochromis
1564.	*Trylochromis polylepis*	Lake Tanganyika tylochromis
1565.	*Tyttocharax madeirae*	Bristle-mouthed tetra
1566.	*Uara amphiacanthoides*	Triangle cichlid/uaru
1567.	*Urumara rondoni*	Mousetail knife fish
1568.	*Vesicatrus tegatus*	Tegatus
1569.	*Wallago attu*	Sheat catfish
1570.	*Xenocara dolichoptera*	Blue chin
1571.	*Xenochromis spp*	
1572.	*Xenomystus nigri*	Black/African knife fish
1573.	*Xenophorus captivus*	Green goodeid
1574.	*Xenotilapia flavipinnis*	Yellow-finned xenotilapia
1575.	*Xenotilapia ochrogenys*	Red-striped xenotilapia
1576.	*Xenotilapid sima*	Big-eyed xenotilapia
1577.	*Xenotilapia spiloptrus*	Spilopterus
1578.	*Xenotoca eiseni*	Dusky goodeid
1579.	*Xenotoca melanosoma*	Red-tailed goodeid
1580.	*Xiphophorus cortezi*	Cortez swordtail
1581.	*Xiphophorus couchianus*	Montery platy
1582.	*Xiphophorus helleri*	Sword-tail
1583.	*Xiphophorus maculates*	Red platy
1584.	*Xiphophorus montezumae*	Montezuma swardtail
1585.	*Xiphophorus pygmaeus*	Pygmy swordtail
1586.	*Xiphophorus variatus*	Variegated/sunset platy
1587.	*Xiphophorus xiphidium*	Northern swordtail platy
1588.	*Zoogoneticus quitzeoensis*	Picotee goodeid
1589.	*Xiphophorus cortezi*	Cortez swordtail
1590.	*Xiphophorus couchianus*	Montery platy
1591.	*Xiphophorus helleri*	Sword-tail
1592.	*Xiphophorus maculates*	Red platy
1593.	*Xiphophorus montezumae*	Montezuma swardtail
1594.	*Xiphophorus pygmaeus*	Pygmy swordtail
1595.	*Xiphophorus variatus*	Variegated/sunset platy
1596.	*Xiphophorus xiphidium*	Northern swordtail platy
1597.	*Zoogoneticus quitzeoensis*	Picotee goodeid

2.6 Fishes (Marine) and Other Marine Species

Still under discussion.

2.7 Invertebrates (Fresh-water)

Still under discussion.

2.8 Invertebrates (Terrestrial)

No	Species	Family
	**Insects for the biological control of invasive alien plants**	
1	*Acacia malherbae*	Eriophyidae
2	*Acanthoscelides macrophthalmus*	Bruchidae
3	*Agrilus hyperici*	Buprestidae
4	*Alagoasa parana*	Chrysomelidae
5	*Alcidion cereicola*	Cerambycidae
6	*Algarobius bottimeri*	Bruchidae
7	*Algarobius prosopis*	Bruchidae
8	*Aphis chloris*	Aphididae
9	*Aplocera efformata*	Geometridae
10	*Autoplusia illustrate*	Noctuidae
11	*Calycomyza lantanae*	Agromyzidae
12	*Carposina autologa*	Carposinidae
13	*Charidotus auroguttata*	Chrysomelidae
14	*Chrysolina hyperici*	Chrysomelidae
15	*Chrysolina quadrigemina*	Chrysomelidae
16	*Cydmaea binotata*	Curculionidae
17	*Cyrtobagous salviniae*	Curculionidae
18	*Dactylopius austrinus*	Pseudococcidae
19	*Dactylopius ceylonicus*	Pseudococcidae
20	*Dactylopius coccus*	Pseudococcidae
21	*Dactylopius opuntiae*	Pseudococcidae
22	*Dactylopius tomentosus*	Pseudococcidae
23	*Dasineura dielsi*	Cecidomyidae
24	*Dasineura xxx Leptosp.*	Cecidomyidae
25	*Eccritotarsus catarinensis*	Miridae
26	*Erytenna consputa*	Curculionidae
27	*Eutreta xanthochaeta*	Tephritidae
28	*Eutreta xanthochaeta*	Tephritidae
29	*Falconia intermedia*	Miridae
30	*Frumenta nephelomicta*	Gelechiidae
31	*Gargaphia decoris*	Tingidae
32	*Gratiana spadicea*	Chrysomelidae
33	*Hypogeococcus festerianus*	Pseudococcidae
34	*Leptinotarsa defecta*	Chrysomelidae
35	*Leptinotarsa texana*	Chrysomelidae
36	*Leptobyrsa decora*	Tingidae
37	*Lysathia sp.*	Chrysomelidae
38	*Melanterius species (all)*	Curculionidae
39	*Mimorista pulchellais*	Pyralidae
40	*Neltumius arizonensis*	Bruchidae
41	*Neochetina bruchi*	Curculionidae
42	*Neochetina eichhorniae*	Curculionidae
43	*Neodiplograsmmus quadrivittatus*	Curculionidae
44	*Neohydronomus affinis*	Curculionidae
45	*Niphograpta albiguttalis*	Pyralidae
46	*Octotoma championi*	Chrysomelidae
47	*Octotoma scrabipennis*	Chrysomelidae
48	*Ophiomyia lantanae*	Agromyzidae
49	*Orthogalumna terebrantis*	Galumnidae
50	*Parectopa thalassias*	Gracillariidae
51	Pareuchaetes aurata	Arctiidae
52	*Pareuchaetes pdeudoinsulata*	Arctiidae
53	*Phenrica guerini*	Chrysomelidae
54	*Plagiohammus spinipennis*	Cerambycidae
55	*Procecidochares utilis*	Tephritidae
56	*Procecidochares utilis*	Tephritidae
57	*Rhynocyllus conicus*	Curculionidae
58	*Rhyssomatus marginatus*	Curculionidae
59	*Sulcobruchus bakeri*	Bruchidae
60	*Teleonemia elata*	Tingidae
61	*Teleonemia scrupulosa*	Tingidae
62	*Trichapion lativentre*	Apionidae
63	*Trichilogaster xxx*	Pteromalidae
64	*Trichilogaster acaciaelongifoliae*	Pteromalidae
65	*Tucumania tapiacola*	Pyralidae
66	*Urophora stylata*	Tephritidae
67	*Urophora stylata*	Tephritidae
68	*Uroplata fulvopustula*	Chrysomelidae
69	*Uroplata girardi*	Chrysomelidae
70	*Uroplata lantanae*	Chrysomelidae

71	*Zeuxidiplosis giardi*	Cecidomyidae
	**Commercially available natural enemies for insect pest control**	
	**Insects**	
1	*Amblyseius californicus*	Phytoseiidae
2	*Amblyseius cucmeris*	Phytoseiidae
3	*Aphidius matricariae*	Braconidae
4	*Aphytis linganensis*	Aphelinidae
5	*Aphytis melinus*	Aphelinidae
6	*Apidoletes aphidimyza*	Braconidae
7	*Chilocorus nigritus*	Coccinellidae
8	*Coccidoxenoides peregrinus*	Coccinellidae
9	*Cryptolaemus montrouzieri*	Coccinellidae
10	*Cybocephalus binotatus*	Coccinellidae
11	*Dacnusa sibirica*	Braconidae
12	*Diglyphus isae*	Braconidae
13	*Encarsia formosa*	Aphelinidae
14	*Nephus reunioni*	Coccinellidae
15	*Phytoseiulus persimilis*	Phytoseiidae
16	*Trichogramma pretiosum*	Trichogrammatidae
17	*Trichogrammatoidea cryptophlebia*	Trichogrammatidae
	**Nematodes**	
1	*Deladenus siricidicola*	
2	*Heterorhabdites megidis*	
3	*Heterorhabditis becteriophora*	
4	*Phasmarhabdites hermaphrodita*	
5	*Steinernema carpocaosae*	
6	*Steinernema feltiae*	
7	*Steinernema glaseri*	
8	*Steinernerma riobrave*	
9	*Steinernerma scapterisci*	
	**Introduced and established natural insect enemies against alien insect pests**	
1	*Acerophagus notativentris*	Encyrtidae
2	*Anagyrus bipunctata*	Encyrtidae
3	*Anaphes nitens*	Mymaridae
4	*Apanteles subandinus*	Braconidae
5	*Aphelinis asychis*	Aphelinidae
6	*Aphelinus hordei*	Aphelinidae
7	*Aphelinus mali*	Aphelinidae
8	*Aphelinus varipes*	Aphelinidae
9	*Aphidius matricariae*	Braconidae
10	*Aphytis chionaspis*	Aphelinidae
11	*Aphytis chrysomphali*	Aphelinidae
12	*Aphytis coheni*	Aphelinidae
13	*Aphytis holoxanthus*	Aphelinidae
14	*Aphytis lepidosaphes*	Aphelinidae
15	*Aphytis linganensis*	Aphelinidae
16	*Aphytis melinus*	Aphelinidae
17	*Ascogaster quadridentata*	Braconidae
18	*Avetianella longoi*	Encyrtidae
19	*Bdellodes lapidaria*	Ibaliidae
20	*Chilocorus cacti*	Coccinellidae
21	*Coccophagus gurneui*	Aphelinidae
22	*Comperiella bifasciata*	Encyrtidae
23	*Copidosoma koehleri*	Encyrtidae
24	*Cryptolaemus montrouzieri*	Coccinellidae
25	*Dendrosoter caenopachoides*	Braconidae
26	*Encarsia cf. smithii*	Aphelinidae
27	*Ennogera politica*	Pteromalidae
28	*Ennogera reticulate*	Pteromalidae
29	*Eretmocereus serius*	Aphelinidae
28	*Ibalia leucospoides*	Ibaliidae
29	*Jarra phoracanthae*	Braconidae
30	*Pauesia cinarivora*	Braconidae
31	*Rodolia cardinalis*	Coccinellidae
32	*Syngaster lepidus*	Braconidae
33	*Trisolcus basalis*	Scelionidae

2.9 Plants

	Species	Common name
**Cultivated crops**		
1.	***Aliem cepa***	Union
2.	***Alium porrum***	Leek
3.	***Anthephora pubescens***	Bottle brush grass
4.	***Arachis hypogeae***	Groundnut
5.	***Asparagus officinalis***	Asparagus
6.	***Avena nuda***	Naked oats
7.	***Avena sativa (A.byzantina)***	Oats
8.	***Beta vulgaris*** (and varieties)	Fodder/garden bee, Swiss chardt
9.	***Brassica napus*** (and var. ***napobrassica)***	Forage rape
10.	***Brassica oleraceae*** (and varieties)	Fodder kal,kohlrabi, curly cale, cauliflower, broccoli, cabbage, savoy cabbage, brussel sprouts
11.	***Brassica rapa (B. chinensis, B. campestris and B.pekinensis)***	Turnip
12.	***Bromus catharticus***	Rescue grass
13.	***Capsicum sp.***	Peppers, paprika
14.	***Cenchrus ciliaris***	Blue buffalo grass
15.	***Chloris gayana***	Rhodes grass
16.	***Citrullus lanatus***	Watermelon
17.	***Cucumis melo***	Sweet melon
18.	***Cucumis sativus***	Cucumber
19.	***Cucurbita maxima***	Pumkin, squash
20.	***Cucurbita moschata***	Pumkin, squash
21.	***Cucurbita pepo***	Squash
22.	***Cydonia sp.***	Quince
23.	***Dactylis glomerata***	Cocksfoot
24.	***Daucus carota***	Carrot
25.	***Desmodium intortum***	Green leaf desmodium
26.	***Desmodium uncinatum***	Silver leaf desmodium
27.	***Digitaria smutsii***	Smuts finger grass
28.	***Eragrostis curvula***	Weeping lovegrass
29.	***Eragrostis tef***	Teff
30.	***Festuca arundinaceae***	Teff fescue
31.	***Fragaria x ananassa***	Strawberry
32.	***Glycine max***	Soya bean
33.	***Gossypium hirsutum***	Cotton
34.	***Helianthus anuus***	Sunflower
35.	***Hordeum vulgare***	Barley
36.	***Lactuca sativa***	Lettuce
37.	***Lespedeza cuneata***	Lespedeza
38.	***Lespedeza striata***	Lespedeza
39.	***Lolium x boucheanum***	Hybrid ryegrass
40.	***Lolium multiflorum***	Italian and westerworld ryegrass
41.	***Lolium perenne***	Perennial ryegrass
42.	***Lolium rigidum***	Annual ryegrass
43.	***Lotus corniculatus***	Bird's foot trefoil
44.	***Lupinus albus***	White lupin
45.	***Lupinus angustifolius***	Narrow leaf lupin
46.	***Lupinus luteus***	Yellow lupin
47.	***Lycopersicon esculentum***	Tomato
48.	***Malus spp***	Apple
49.	***Medicago littoralis***	Strand medic
50.	***Medicago polymorpha***	Burr medic
51.	***Medicago rugosa***	Gama medic
52.	***Medicago sativa (M. varia)***	Lucerne
53.	***Medicago scutellata***	Snail medic
54.	***Nicoteana tabacum***	Tobacco
55.	***Ornithopus compressus***	Yellow serradella
56.	***Ornithopus sativus***	Serradella
57.	***Oryza sativa***	Rice
58.	***Panicum maximum***	White buffalo grass
59.	***Paspalum dilatatum***	Dallis grass
60.	***Pastinaca sativa***	Parsnip
61.	***Pennisetum clandestinum***	Kikuyu
62.	***Pennisteum glaucum***	Pearl millet
63.	***Petroselinum crispum***	Parsley
64.	***Phalaris aquatica***	Phalaris
65.	***Phaseolus coccineus***	Kidney bean
66.	***Phaseolus vulgaris***	Dry bean, garden bean
67.	***Pisum sativum***	Dry pea, garden pea
68.	***Prunus amygdalus***	

69.	***Prunus dulcis***	Almond
70.	***Prunus armeniaca***	Apricot
71.	***Prunus avium***	Sweet cherry
72.	***Prunus cerasus***	Sour cherry
73.	***Prunus domestica***	Plum
74.	***Prunus persica and varieties***	Peach, nectarine
75.	***Prunus salicina***	Japanese plum
76.	***Pyrus sp.***	Pear
77.	***Raphanus sativus*** and varieties	Fodderand garden radish
78.	***Ricinus communis***	Castor bean (castor oil pnt)
79.	***Secale cereale***	Rye
80.	***Setaria sphacelata***	Common setaria
81.	***Sinapsis alba***	White mustard
82.	***Solanum melongena***	Eggplant
83.	***Solanum tuberosum***	Potato
84.	***Sorghum bicolor***	Grain sorghum
85.	***Sorghum almum***	Forage sorghum
86.	***Sorghum sudanense***	Forage sorghum
87.	***Stylosanthes hamate***	Caribbean stylo
88.	***Trifolium fragiferum***	Strawberry clover
89.	***Trifolium hirtum***	Rose clover
90.	***Trifolium incamatum***	Crimson clover
91.	***Trifolium pratense***	Red clover
92.	***Trifolium repens***	White clover
93.	***Trifolium resupinatum***	Persian clover
94.	***Trifolium subterraneum***	Subterranean clover
95.	***Trifolium vesiculosum***	Arrow leaf clover
96.	***Triticum x secale***	Triticale
97.	***Triticum aestivum***	Wheat
98.	***Triticum durum***	Durum wheat
99.	***Vicia faba***	Broad bean
100.	***Vicia sativa***	Common vetch
101.	***Vicia villosa***	Hairy vetch
102.	***Vigna unguiculata***	Cowpea
103.	***Vitis sp***	Grape
104.	***Zea mays***	White and yellow maize, sweetcorn
**Ornamental plants**		
These are still under discussion.		

2.10 Microbes

No	Species	Benefit
	**Bacteria**	
1	*Agrobacterium radiobacter*	Biocontrol crown gall
2	*Azospirillum brasiliense*	Plant growth stimulant
3	*Azospirillum lipoferum*	Plant growth stimulant
4	*Bacillus licheniformis* (Bacterium)	Foliar and soil fungal pathogen control
5	*Bacillus pumillus* (Bacterium)	As above
6	*Bacillus sphaericus* (Bacillaceae)	Insect pest control
7	*Bacillus subtilis* (Bacterium)	Biocontrol soil fungal pathogens
8	*Bacillus thuringiensis* (Bacillaceae)	Insect pest control
9	*Paenibacillus popillae* (Paenibacillaceae)	Insect pest control
10	*Rhyzobium lophantae*	
11	*Serratia entomophila* (Enterobacteriaceae)	Biocontrol insect pests
	**Fungi**	
1	*Beauveria bassiana* (Deuteromycetes)	Biocontrol insect pests
2	*Beauveria brongniartii* (Deuteromycetes)	Biocontrol insect pests
3	*Cryptococcus albidus* (Hyphomecetes)	Biocontrol of pathogens
4	*Entyloma ageratinae* (Basidiomycetes)	Biocontrol of Ageratina
5	*Lagenidium giganteum* (Oomycetes)	Biocontrol insect pests
6	*Lecanicillium lecanii* (Deuteromycetes)	Biocontrol insect pests
7	*Metarhizium anisoplae* (Hyphomycetes)	Biocontrol of locusts
8	*Mycovellosiella lantantae* var. *lantanae*	Biocontrol of Lantana
9	*Paecilomyces lilacinus* (Hyphomycetes)	Biocontrol nematodes
10	*Paecilomycetes fumosoroseus* (Hyphomycetes)	Biocontrol nematodes
11	*Pandora neoaphidis* (Hyphomycetes)	Biocontrol of aphids
12	*Trichoderma harzianum*	Biocontrol of pathogens
13	*Trichoderma koningii*	Biocontrol of pathogens
14	*Trichoderma viridae*	Biocontrol of pathogens
15	*Uromycladium tepperianum* (Basiodiomycetes)	Biocontrol of *Acacia saligna*
16	*Verticillium chlamydosporium* (Hyphomycetes)	Biocontrol nematodes
17	*Zoophthora radicans* (Entomophthoraceae)	Biocontrol insects
	**Microsporidia**	

1	*Nosema locustae (*Nosematidae*)*	Biocontrol of locusts
2	*Varimorpha necatrix (*Burenellidae*)*	
	**Virusses**	
1	*Cydia pomonella* granulosus virus (CpGV)	Biocontrol of codling moth
2	*Helicoverpa armigera* Nucleopolyhedrovirus (SeNVP) Baculoviridae	Biocontrol of stalk borer
3	*Spodoptera exigua* (Nucleopolyhedrovirus) (SeNVP) Baculoviridae	Biocontrol of armyworm

Appendix 3

Species Listed as Invasive Species

1. The species are listed alphabetically by scientific name for each of the following groupings:
   3.1 Mammals
   3.2 Birds
   3.3 Reptiles
   3.4 Amphibians
   3.5 Fishes (Fresh-Water)
   3.6 Fishes (Marine) and Other Marine Species
   3.7 Invertebrates (Fresh-water)
   3.7 Invertebrates (Terrestrial)
   3.9 Plants
   3.10 Microbes

2. The following categories of invasive species are provided for in terms of the Regulations:
   Category 1a: Prohibited Invasive Species
   Category 1b: Invasive Species Controlled by Programme
   Category 2: Invasive Species Controlled by Area
   Category 3: Invasive Species Controlled by Activity
   Category 4: Extra-Limital Species
   Category 5: Species Under Surveillance

3. The conditions set out in the Regulations, and in Schedule 3 to the Regulations, apply to the regulation of the species listed in these six categories.

4. Category 4 species are only considered invasive outside of the area deemed as part of the distribution range of a species indigenous to South Africa.

5 Category 5 species are potentially invasive species, or invasive species where the benefits of controlling them are to be weighed up against the costs of doing so.

6. The maps showing the deemed natural distribution ranges for the extra-limital species (Category 2) can be found in Schedule 3 to the Regulations.

7. The lists will be consolidated over the next two years, and updated on a regular basis.

3.1 Mammals

No	Species	Common Name	Category
1	*Addax nasomaculatus*	Addax	2
2	*Aepyceros melampus melampus*	Impala	4 (Map)
3	*Aepyceros melampus petersi*	Black-faced impala	2
4	*Alcelaphus lichtensteinii*	Lichtenstein's hartebeest	2
5	*Ammotragus lervia*	Barbary sheep	2
6	*Antilope cervicapra*	Indian blackbuck	2
7	*Axis axis*	Axis deer (Chital)	2
8	*Axis porcinus*	Hog deer	2
9	*Boselaphus tragocamelus*	Nilgai	2
10	*Capra hircus*	Feral goat	1b - for islands
11	*Cephalophus natalensis*	Red duiker	4 (Map)
12	*Cercopithecus mitis*	Samango monkey	4 (Map)
13	*Cervus elaphus*	Red deer	2
14	*Cervus nippon*	Sika deer	2
15	*Chlorocebus aethiops*	Vervet monkey	4 (Map)
16	*Connochaetes gnou*	Black wildebeest	4 (Map)
17	*Connochaetes gnou x taurinus taurinus*	Hybrid (black / blue wildebeest)	1a
18	*Connochaetes taurinus taurinus*	Blue wildebeest	4 (Map)
19	*Dama dama*	Fallow deer	2
20	*Damaliscus pygargus phillipsi*	Blesbok	4 (Map)
21	*Damaliscus pygargus phillipsi x pygargus pygargus*	Hybrid (blesbok / bontebok)	1a
22	*Damaliscus pygargus pygargus*	Bontebok	4 (Map)
23	*Diceros bicornis*	Black south-western rhinoceros	4 (Map)
24	*Diceros bicornis michaeli*	Black Kenya rhinoceros	2
25	*Diceros bicornis minor*	Black south-central rhinoceros	4 (Map)
26	*Elaphurus davidianus*	Pere David's deer	2
27	*Equus zebra hartmannae*	Hartmann's mountain zebra	4 (Map)
28	*Equus zebra zebra*	Cape mountain zebra	4 (Map)
29	*Felis catus*	Domestic cat	1b on island
30	*Giraffa camelopardalis giraffa*	Giraffe	4 (Map)
31	*Hemitragus jemlahicus*	Himalayan tahr	1b
32	*Hippotragus koba*	Western roan	2
33	*Hydrochaeris hydrochaeris*	Cappibara	3
34	*Kobus ellipsiprymnus crawshayi*	Crawshay's waterbuck (Zambia)	2
35	*Kobus ellipsiprymnus defassa*	Defassa waterbuck (Kenya)	3
36	*Kobus leche*	Red lechwe	2
37	*Kobus vardonii*	Puku	2
38	*Mus musculus*	House mouse	1b on islands
39	*Myocastor coypus*	Coypu	3
40	*Neotragus moschatus zuluensis*	Suni	4 (Map)
41	*Oreotragus oreotragus*	Klipspringer	4 (Map)
42	*Oryctolagus cuniculus*	European rabbit	1b on islands
43	*Oryx dammah*	Scimitar-horned oryx	2
44	*Ourebia ourebi*	Oribi	4 (Map)
45	*Ovis musimon*	Mouflon	2
46	*Panthera pardus melanotica*	Leopard	5
47	*Papio cynocephalus*	Baboon	5
48	*Papio ursinus*	Baboon	4 (Map)
49	*Pelea capreolus*	Grey rhebok	4 (Map)
50	*Phacochoerus africanus*	Warthog	4 (Map)
51	*Philantomba monticola*	Blue duiker	4 (Map)
52	*Potamochoerus larvatus koiropotamus*	Bushpig	4 (Map)
53	*Raphicerus campestris*	Steenbok	4 (Map)

54	*Raphicerus melanotis*	Cape grysbok	4 (Map)
55	*Raphicerus sharpei*	Sharpe's grysbok	4 (Map)
56	*Rattus norvegicus*	Brown rat	1b for islands
57	*Rattus rattus*	House rat	1b for islands
58	*Redunca arundinum arundinum*	Southern reedbuck	4 (Map)
59	*Redunca fulvorufula*	Mountain reedbuck	4 (Map)
60	*Rusa unicolor*	Sambar deer	2
61	*Sciurus carolinensis*	Grey squirrel	5
62	*Silvicapra grimmia*	Gray Duiker	4 (Map)
63	*Sus scrofa*	Feral pig	1b
64	*Tragelaphus derbianus*	Derby eland	3
65	*Tragelaphus angasii*	Nyala	4 (Map)
66	*Tragelaphus scriptus*	Bushbuck	4 (Map)
67	*Tragelaphus spekii*	Sitatunga	2
68	*Tragelaphus livingstonii*	Livingstone's eland	2

3.2 Birds

No	Species	Common Name	Category
1	*Acridotheres tristis*	Indian myna	2
2	*Alectoris chukar*	Chukar partridge	2 on mainland; 1b on islands
3	*Alopochen aegyptiacus*	Egyptian goose	5
4	*Anas platyrhynchos*	Mallard duck	1b
5	*Corvus splendens*	Indian house crow	1b
6	*Cygnus olor*	Mute swan	5
7	*Fringilla coelebs*	Chaffinch	5
8	*Numida meleagris coronata*	Helmeted guineafowl (Eastern Cape)	4 (Map)
9	*Numida meleagris galeata*	Helmeted guineafowl (West African)	3
10	*Oxyura leucocephala*	White-headed duck	5
11	*Passer domesticus*	House sparrow House	5
12	*Pavo cristatus*	Peacock	5
13	*Psittacula cyanocephala*	Plumheaded parakeet	5
14	*Psittacula krameri*	Rose-ringed parakeet	5
15	*Sturnus vulgaris*	Eurasian starling	2

3.3 Reptiles

No	Species	Common Name	Category
1	*Acanthophis antarcticus*	Grey death adder	5
2	*Acanthophis pyrrhus*	Desert death adder	5
3	*Afroedura pondolia*	Pondola fat-tailed gheko	5
4	*Afrogecko porphyreus*	Marbled gheko	5 on islands
5	*Agkistrodon contortrix*	Copperhead	5
6	*Agkistrodon piscivorous*	Cottonmouth	5
7	*Agkistrodon contortrix*	Copperhead	5
8	*Agkistrodon piscivorus*	Water/cottonmouth moccasin	5
9	*Aspidelaps cowlesi*	Angolan coral snake	5
10	*Aspidelaps fulafula*	Eastern shieldnose	5
11	*Bitis gabonica rhinoceros*	West Arican gaboon viper	5
12	*Bogertophis subocularis*	Ratsnake transpecos	5
13	*Bradypodion damaranum*	Knysna dwarf chameleon	5
14	*Cerastes cerastes*	Desert horned viper	5
15	*Chalcides guentheri*	Gunthers skink	5
16	*Chalcides ocellatus*	Ocellated skink	5

17	*Chameleo calyptratus*	Yemen chameleon	5
18	*Charina bottae*	Rubber boa	5
19	*Charina trivirgata*	Rosy boa	5
20	*Chelydra serpentina*	Common snapper turtle	1a
21	*Chlamydosaurus kingii*	Frilled lizard	5
22	*Coluber hippocrepis*	Whip snake	5
23	*Crotalus atrox*	Western diamond-backed rattler	5
24	*Crotalus horridus*	Canebrake timber/rattlesnake	5
25	*Crotalus scutulatus*	Mojave rattlesnake	5
26	*Crotalus viridis*	Prairie rattlesnake	5
27	*Crotaphytus collaris*	Collared lizard	5
28	*Cyrtopodion caspius*	Caspian gecko	5
29	*Cyrtopodion kotschyi*	Kotchys gecko	5
30	*Daboia russelli*	Russels viper	5
31	*Drymarchon corais*	Cribo	5
32	*Drymarchon couperi*	Indigo snake	5
33	*Echis carinatus*	Saw scaled viper	5
34	*Echis coloratus*	Carpet viper	5
35	*Egernia cunninghami*	Cunninghams skink	5
36	*Elaphe bairdi*	Bairds ratsnake	5
37	*Elaphe dione*	Diones ratsnake	5
38	*Elaphe emoryi*	Great plains ratsnake	5
39	*Elaphe guttata*	Corn snake	5
40	*Elaphe helena*	Trinket ratsnake	5
41	*Elaphe obsoleta*	American ratsnake	5
42	*Elaphe quatuorlineata*	European ratsnake	5
43	*Eryx colubrinus*	Kenyan sandboa	5
44	*Eryx conicus*	Rough scaled sandboa	5
45	*Eryx miliaris*	Military sandboa	5
46	*Eryx tataricus*	Russian sandboa	5
47	*Eublepharis macularius*	Leopard gecko	5
48	*Eumeces callicephalus*	Mountain skink	5
49	*Eumeces egregious*	Royal skink	5
50	*Eumeces elegans*	Five lined skink	5
51	*Gekko gecko*	Tokay gecko	3
52	*Geochelone sulcata*	African spur-thighed tortoise	5
53	*Heterodon nasicus*	Western hognose snake	5
54	*Hemidactylus cf. mabouia*	Tropical house gecko	5
55	*Iguana iguana*	Green iguana	3
56	*Lacerta viridis*	Green wall lizard	5
57	*Lampropeltis spp*	Kingsnakes	5
58	*Getulus (ssp. californiae, nigritus, splendida)*	Californian/Mxican/desert kingsnake	5
59	*Lampropeltis mexicana (ssp. alterna)*	Gray-banded kingsnake	5
60	*Lampropeltis pyromelana*	Arizona mountain kingsnake	5
61	*Lampropeltis triangulum*	Mexican milk snake	5
62	*Lamprophis (Boaedon) lineatus*	Lined house snake	5
63	*Lamprophis (Boaedon) olivaceus*	Olive house snake	5
64	*Leioheterodon geayi*	Speckled hognosed snake	5
65	*Leioheterodon madagascariensis*	Giant hognosed snake	5
66	*Leioheterodon modestus*	Blond hognosed snake	5
67	*Lepidodactylus lugubris*	Mourning gecko	5
68	*Macrovipera deserti*	Desert viper	5
69	*Macrovipera lebetina*	Levant viper	5
70	*Naja nigricincta*	Western barred cobra	5
71	*Naja pallida*	Red spitting cobra	5
72	*Natrix maura*	Checkered grass snake	5

73	*Natrix natrix*	European grass snake	5
74	*Ophisaurus apodus*	European legless lizard	5
75	*Uromastyx aegypticus*	Egyptian spiny-tailed lizard	5
76	*Phelsuma lineata*	Lined day gecko	5
77	*Phelsuma madagascariensis*	Giant day gecko	5
78	*Phelsuma standingi*	Yellow spotted day gecko	5
79	*Physignathus cocincinus*	Chinese water dragon	5
80	*Physignathus lesueurii*	Australian water dragon	5
81	*Pituophis catenifer*	Pine and gopher snakes	5
82	*Pituophis melanoleucus*	Bullsnake	5
83	*Pogona barbata*	Bearded dragon	5
84	*Pogona vitticeps*	Inland bearded dragon	5
85	*Pseudocerastes fieldi*	False horned viper	5
86	*Ptyas mucosus*	Indian ratsnake	5
87	*Rankinia adelaidensis*	Jacky dragon	5
88	*Rankinia diemensis*	Mountain dragon	5
89	*Rhinotyphlops*	Spp	1a
90	*Sistrurus catenatus*	Massasauga	5
91	*Sistrurus miliarius*	Pigmy rattlesnake	5
92	*Sistrurus ravus*	Mexican pigmy rattlesnake	5
93	*Spalaerosophis diadema*	Diadem ratsnake	5
94	*Teratolepis fasciata*	Viper gecko	5
95	*Thamnophis marcianus*	Chequered garter snake	5
96	*Trachemys scripta elegans*	Red-eared slider	1a
97	*Thamnophis sirtalis*	Common garter snake	5
98	*Trachydosaurus rugosa*	Pine cone skink	5
99	*Tupinambis merianae*	Argentine tegu	5
100	*Tupinambis rufescens*	Red tegu	5
101	*Tympanocryptis cephalus*	Earless dragon	5
102	*Tympanocryptis lineata*	Striped dragon	5
103	*Uromastyx acanthinura*	North African dab lizard	5
104	*Uromastyx gehri*	Nigerian dab lizard	5
105	*Uromastyx malienis*	Mali dab lizard	5
106	*Uromastyx ocellata*	Spotted dab lizard	5
107	*Uromastyx ornata*	Egyptian dab lizard	5
108	*Varanus acanthurus*	Ridge tailed monitor	5
109	*Varanus bengalensis*	Bengal monitor	5
110	*Varanus gouldii*	Goulds monitor	5
111	*Vipera ammodytes*	Long nose viper	5
112	*Vipera berus*	European adder	5
113	*Vipera kaznakovi*	Russian adder	5
114	*Vipera palaestinae*	Palestine viper	5
115	*Vipera raddei*	Radds viper	5
116	*Vipera xanthina*	Ottomans viper	5
117	*Zonosaurus madagascariensis*	Madagascan plated lizard	5
118	*Zonosaurus ornatus*	Ornate plated lizard	5
119	*Zonosaurus quadrilineatus*	Four lined plated lizard	5

3.4 Amphibians

No	*Species*	*Common Name*		Category
1	*Bufo gutturalis*	Guttural toad	5	
2	*Hyperolius marmoratus*	Painted reed frog	4	

3.5 Fishes (Fresh-water)

No	Species	Common name	Category
1	*Carassius auratus*	goldfish	5
2	*Clarias gariepinus*	sharptooth catfish	4
3	*Ctenopharyngodon idella*	grass carp	1b
4	*Ctenopharyngodon idella (triploid)*	grass carp (triploid)	2
5	*Cyprinus carpio*	carp	2
6	*Cyprinus carpio*	koi carp	5
7	*Gambusia affinis*	mosquitofish	1b
8	*Hypopthalmichthys molitrix*	silver carp	1b
9	*Labeo capensis*	Orange River mudfish	4
10	*Labeo umbratus*	moggel	4
11	*Labeobarbus aeneus*	Vaal-Orange smallmouth yellowfish	4
12	*Labeobarbus capensis*	Clanwilliam yellowfish	4
13	*Labeobarbus natalensis*	KwaZulu-Natal yellowfish	4
14	*Lepomis macrochirus*	bluegill sunfish	1b
15	*Micropterus dolomieu*	smallmouth bass	2
16	*Micropterus punctulatus*	spotted bass	2
17	*Micropterus salmoides*	largemouth bass	2
18	*Oncorhynchus mykiss*	rainbow trout	2
19	*Oreochromis mossambicus*	Mocambique tilapia	4
20	*Oreochromis niloticus*	Nile tilapia	1b
21	*Salmo trutta*	brown trout	2
22	*Sandelia capensis*	Cape kurper	4
23	*Tilapia rendalli*	redbreast tilapia	4
24	*Tilapia sparrmanii*	banded tilapia	4
25	*Tinca tinca*	Tench	1b

3.6 Fishes (Marine) and other Marine Species

No	Species	Common Name	Category
1	*Carcinus maenas*	European shore crab / Green crab	1b
2	*Mytilus galloprovincialis*	Mediterranean mussel	1b
3	All introduced aquaculture species	SEE POINTS 1 & 2 BELOW	2

NB.
1. Lists to be completed (eg, Japanese oyster, Atlantic salmon, Turbot)
2. Sale of live oysters not covered by (3), but may not be returned to the sea.

3.7 Invertebrates (Fresh-water)

No	*Species*	Common Name	Category
1	*Aedes albopictus*	Asian tiger mosquito	1b
2	*Aplexa marmorata*	marbled tadpole snail	1b
3	*Cherax quadricarinatus*	Redclaw crayfish	1b
4	*Cherax tenuimanus*	marron	2
5	*Lymnaea columella*	amphibious pond snail	1b
6	*Marisa cornuarietis*	apple snail	1a
7	*Procambarus clarkii*	red swamp crayfish	1a
8	*Tarebia granifera*	quilted malania	1b

3.8 Invertebrates (Terrestrial)

No	Species	Common Name	Category
1	*Apis mellifera scutellata*	African bee	4
2	*Apis mellifera capensis*	Cape bee	4
3	*Chlorophorus annularis* (Fabricius)	Bamboo longhorn	5
4	*Cosmopolites sordidus* (Germar)	Banana root borer	1b
5	*Dirofilaria immitis* Leidy, 1856	Heartworm nematode	1b
6	*Ditylenchus destructor* Thorne, 1945	Potato rot nematode	*1b*
7	*Ditylenchus dipsaci* (Kühn, 1857) Filip'ev, 1936	Stem and bulb nematode	*1b*
8	*Globodera rostochiensis* (Wollenweber, 1923), Behrens, 1975	Golden cyst nematode; potato cyst nematode.	*1b*
9	*Linepithema humile* (Mayr)	Argentine ant	5
10	*Meloidogyne partityla* Kleynhans, 1986	'Pecan nut" nematode	*1b*
11	Oxychilus draparnaudi *(Beck, 1837)*	Draparnaud's glass snail	5
12	*Pieris brassicae* Linnaeus	Cabbage white	5
13	*Prostephanus truncatus* (Horn)	Larger grain borer	5
14	*Radopholus similis* (Cobb, 1893) Thorne, 1949	Burrowing nematode	*1b*
15	*Theba pisana (Muller, 1774)*	white garden snail / vine snail / dune snail / white Italian snail	5
16	*Varroa destructor Anderson & Trueman*	Varroa mite	*1b*
17	*Vespula germanica (Fabricius)*	European wasp	*1b*

3.9 Plants

No	Species	Common name	Category
1	*Acacia baileyana* F.Muell.	Bailey's wattle	3
2	*Acacia cyclops* A.Cunn. ex G.Don	Red eye	2
3	*Acacia dealbata* Link	Silver wattle	Category 1a plant in Western Cape, Northern Cape, Limpopo and Free State. Category 1b plant in rest of South Africa
4	*Acacia decurrens* Willd.	Green wattle	Category 1a plant in Western Cape, Northern Cape, Limpopo and Free State. Category 1b plant in rest of South Africa
5	*Acacia elata* A.Cunn. ex Benth. (*Acacia terminalis* misapplied in South Africa)	Pepper tree wattle	1b
6	*Acacia implexa* Benth.	Screw pod wattle	1a
7	*Acacia longifolia* (Andrews) Willd.	Long-leaved wattle	1b
8	*Acacia mearnsii* De Wild.	Black wattle	2
9	*Acacia melanoxylon* R.Br.	Australian blackwood	Category 2 in the Garden Route from Tsitsikamma to George. Category 1b in rest of South Africa
10	*Acacia paradoxa* DC. (= *A. armata* R.Br.)	Kangaroo wattle	1a
11	*Acacia pendula* A.Cunn. ex G.Don	Weeping myall	5
12	*Acacia podalynifolia* A.Cunn. ex G.Don	Pearl acacia	3
13	*Acacia pycnantha* Benth.	Golden wattle	1b
14	*Acacia saligna* (Labill.) H.L.Wendl.	Port Jackson willow	2
15	*Acacia stricta* (Andrews) Willd.	Hop wattle	1a
16	*Acer buergerianum* Miq.	Chinese maple	5
17	*Acer negundo* L.	Ash-leaved maple, Box elder	5
18	*Agave americana* L. var *americana*	American agave, Century-plant	5
19	*Agave americana* L. var *expansa* (Jacobi) Gentry	Spreading century-plant	Category 1a in Western Cape coastal belt
20	*Agave sisalana* Perrine	Sisal hemp, Sisal	2
21	*Ageratina adenophora* (Spreng.) R.M.King & H.Rob. (= *Eupatorium adenophorum* Spreng.)	Crofton weed	1a
22	*Ageratina riparia* (Regel) R.M.King & H.Rob. (= *Eupatorium riparium* Regel)	Mistflower	1a
23	*Ageratum conyzoides* L.	Invading ageratum	1b
24	*Ageratum houstonianum* Mill. Excluding cultivars	Mexican ageratum	1b

25	*Agrimonia procera* Wallr.	Scented agrimony	5
26	*Agrostis castellana* Boiss. & Reut.	Bent grass	Category 1a on Prince Edward Island. Category on 1b Marion Island
27	*Agrostis gigantea* Roth	Black bent grass, redtop	Category 1a on Prince Edward and Marion Islands
28	*Agrostis stolonifera* L.	Creeping bent grass	Category 1a on Prince Edward Island. Category 1b on Marion Island
29	*Ailanthus altissima* (Mill.) Swingle	Tree-of-heaven	1b
30	*Albizia julibrissin* Durazz.	Silk tree, Pink siris	5
31	*Albizia lebbeck* (L.) Benth.	Lebbeck tree	1a
32	*Albizia procera* (Roxb.) Benth.	False lebbeck	1a
33	*Alhagi maurorum* Medik.<br>(= *A. camelorum* Fisch.)	Camel thorn bush	1b
34	*Alisma plantago- aquatica* L.	Mud plantain, Water alisma	1b
35	*Alnus glutinosa* (L.) Gaertn.	Black alder	5
36	*Alopecurus geniculatus* L.<br>(= *A. australis* Nees)	Marsh foxtail, Water foxtail	Category 1a on Prince Edward and Marion Islands
37	*Ammophila arenaria* (L.) Link	Marram grass	1b
38	*Anredera cordifolia* (Ten.) Steenis<br>(*A. baselloides* misapplied in South Africa)	Madeira vine, Bridal wreath	1b
39	*Antigonon leptopus* Hook. & Arn.	Coral creeper	1b
40	*Araujia sericifera* Brot.	Moth catcher	1b
41	*Ardisia crenata* Sims<br>(*Ardisia crispa* misapplied in South Africa)	Coralberry tree, Coral Bush	Category 1a in KwaZulu-Natal, Mpumalanga, Limpopo and Eastern Cape
42	*Ardisia elliptica* Thunb.<br>(= *A. humilis* Vahl)	Shoebutton ardisia	Category 1a in KwaZulu-Natal, Mpumalanga, Limpopo and Eastern Cape
43	*Argemone mexicana*	Yellow-flowered Mexican poppy	1b
44	*Argemone ochroleuca* Sweet subsp. *ochroleuca*<br>(= *A. subfusiformis* G.B.Ownbey)	White-flowered Mexican poppy	1b
45	*Aristolochia elegans* Mast.	Dutchman's pipe	1b
46	*Arundo donax* L.	Giant reed	1b
47	*Atriplex inflata* F.Muell.<br>(= *A. lindleyi* Moq. subsp. *inflata* (F. Muell.) Paul G.Wilson)	Sponge-fruit saltbush	1b
48	*Atriplex nummularia* Lindl. Subsp. *Nummularia*	Old man saltbush	2
49	*Avena* spp. excluding *A. sativa*<br>(includes *A. barbata* Pott ex Link, *A. fatua* L., *A. sterilis* L.)	Wild oats	5
50	*Azolla filiculoides* Lam.	Azolla, Red water fern	1b
51	*Bartlettina sordida* (Less.) R.M. King & H.Rob.<br>(= *Eupatorium atrorubens* (Lem.) G.Nicholson, *E. sordidum* Less.)	Bartlettina	1a
52	*Bauhinia purpurea* L.	Butterfly orchid tree	Category 1a in KwaZulu-Natal, Mpumalanga, Limpopo and Eastern Cape
53	*Bauhinia variegata* L.	Orchid tree	Category 1a in KwaZulu-Natal, Mpumalanga, Limpopo and Eastern Cape
54	*Berberis thunbergii* DC.	Japanese barberry	5
55	*Billardiera heterophylla* (Lindl.) L.W.Cayzer & Crisp<br>(= *Sollya heterophylla* Lindl.)	Bluebell creeper	1a
56	*Bougainvillea glabra* Choisy	Bougainvillea, Paper flower	5
57	*Bougainvillea spectabilis* Willd.	Great bougainvillea	5
58	*Brachychiton populneus* (Schott & Endl.) R.Br.	Kurrajong, Bottle tree	3
59	*Bromus* spp.<br>(includes *B. commutatus* Schrad., *B. diandrus* Roth, *B. japonicus* Thunb., *B. hordaceus* L. subsp. *divaricatus* (Bonnier & Layens) Kerguélen (= *B. molliformis* J.Lloyd), *B. catharticus* Vahl var. *catharticus* (= *B. unioloides* Kunth)	Brome spp.	5
60	*Bryophyllum delagoense* (Eckl. & Zeyh.) Schinz<br>(= *B. tubiflorum* Harv., *Kalanchoe tubiflora* (Harv.) Raym.- Hamet, *K. delagoensis* Eckl. & Zeyh.)	Chandelier plant	1b
61	*Bryophyllum pinnatum* (Lam.) Oken	Green mother of millions	1b
62	*Buddleja davidii* Franch.	Chinese sagewood, Summer lilac	3

63	*Buddleja madagascariensis* Lam.	Madagascar sagewood	3
64	*Caesalpinia decapetala* (Roth) Alston (= *C. sepiaria* Roxb.)	Mauritius thorn	1b
65	*Caesalpinia gilliesii* (Hook.) D.Dietr.	Bird-of-paradise flower	1a
66	*Callisia repens* (Jacq.) L.	Creeping inch plant	1a
67	*Callistemon citrinus* (Curtis) Skeels	Lemon bottlebrush	5
68	*Callistemon rigidus* R.Br.	Stiff-leaved bottlebrush	Category 1a in Western and Eastern Cape
69	*Callistemon viminalis* (Sol. ex Gaertn.) G.Don	Weeping bottlebrush	Category 1a in KwaZulu-Natal, Mpumalanga, Limpopo and Eastern Cape
70	*Calotropis procera* (Aiton) W.T.Aiton	Calotropis, Giant-milkweed	5
71	*Campuloclinium macrocephalum* (Less.) DC. (= *Eupatorium macrocephalum* Less.)	Pom pom weed	Category 1b in Gauteng. Category 1a in rest of South Africa
72	*Canna indica* L. Excluding hybrid cultivars	Indian shot	1b
73	*Cardiospermum grandiflorum* Sw.	Balloon vine	1b
74	*Cardiospermum halicacabum* L.	Lesser balloon vine	3
75	*Casuarina cunninghamiana* Miq.	Beefwood	Category 1b in Western Cape. Category 2 in rest of South Africa
76	*Casuarina equisetifolia* L.	Horsetail tree	2
77	*Catharanthus roseus* (L.) G.Don	Madagascar periwinkle	5
78	*Celtis australis* L.	Nettle tree, European hackberry	5
79	*Celtis occidentalis* L.	Common hackberry	5
80	*Celtis sinensis* Pers.	Chinese nettle tree	5
81	*Cerastium fontanum* Baumg.	Common mouse-ear chickweed	Category 1b on Prince Edward and Marion Islands
82	*Cereus jamacaru* DC. (*C. peruvianus* misapplied in South Africa)	Queen of the night	Category 1b in North West, Gauteng, Mpumalanga and Limpopo. Category 1a in rest of South Africa
83	*Cestrum aurantiacum* Lindl.	Yellow or Orange cestrum	1b
84	*Cestrum elegans* (Brongn.) Schltdl. (= *C. purpureum* (Lindl.) Standl.)	Crimson cestrum	1a
85	*Cestrum laevigatum* Schltdl.	Inkberry	1b
86	*Cestrum parqui* L´Hér.	Chilean inkberry	1a
87	*Cestrum* spp. excluding sterile cultivars	Cestrum spp.	1a
88	*Chondrilla juncea* L.	Skeleton weed	1a
89	*Chromolaena odorata* (L.) R.M.King & H.Rob. (= *Eupatorium odoratum* L.)	Triffid weed, Chromolaena	Category 1b in KwaZulu-Natal, Limpopo, Mpumalanga and Eastern Cape north of East – London. Category 1a in rest of South Africa
90	*Chrysopogon zizanioides* (L.) Roberty (= *Vetiveria zizanioides* (L.) Nash)	Vetiver grass	5
91	*Cichorium intybus* L.	Chicory	2
92	*Cinnamomum camphora* (L.) J.Presl	Camphor tree	Category 1b in KwaZulu-Natal, Mpumalanga, Eastern and Western Cape
93	*Cirsium vulgare* (Savi) Ten. (= *C. lanceolatum* (L.) Scop.)	Spear thistle, Scotch thistle	1b
94	*Coffea arabica* L.	Coffee tree	5
95	*Convolvulus arvensis* L.	Field bindweed, Wild morning-glory	1b
96	*Coreopsis lanceolata* L.	Coreopsis, Tickseed	1a
97	*Cortaderia jubata* (Lemoine ex Carrière) Stapf	Pampas grass	Category 1b in Gauteng. Category 1a in rest of South Africa
98	*Cortaderia selloana* (Schult.) Asch. & Graebn. Excluding sterile cultivars	Pampas grass	Category 1b in Western and Eastern Cape, KwaZulu-Natal and Gauteng, Category 1a in rest of South Africa
99	*Cotoneaster franchetii* Bois	Cotoneaster	1b
100	*Cotoneaster glaucophyllus* Franch.	Late cotoneaster	5
101	*Cotoneaster pannosus* Franch.	Silver leaf cotoneaster	1b
102	*Cotoneaster salicifolius* Franch.	Willow-leaved showberry	5
103	*Cotoneaster simonsii* Baker	Himalayan cotoneaster, Simon's cotoneaster	5
104	*Crataegus mexicana* DC. (= *C. pubescens* misapplied in South Africa.)	Mexican hawthorn	5
105	*Crataegus monogyna* Jacq. (= *C. oxyacantha* L. var. *praecox* hort. ex Loudon)	English hawthorn, Common hawthorn	5
106	*Crataegus phaenopyrum* (L.f.) Medik.	Washington thorn	5
107	*Crotolaria agatiflora* Schweinf.	Canarybird bush, bird flower	1a
108	*Cryptostegia grandiflora* R.Br.	Rubber vine	1a
109	*Cryptostegia madagascariensis* Bojer ex Decne.	Madagascar rubber vine	1a

110	*Cuscuta campestris* Yunck.	Common dodder	1b
111	*Cuscuta suaveolens* Ser.	Lucerne dodder	1b
112	*Cyathea australis* (R.Br.) Domin	Rough tree fern	Category 1a in KwaZulu-Natal, Mpumalanga, Limpopo, Eastern Cape, Western Cape
113	*Cyathea cooperi* (Hook. ex F.Muell.) Domin (= *Sphaeropteris cooperi* (Hook. ex F. Muell.) R.M.Tryon)	Australian tree fern	Category 1a in KwaZulu-Natal, Mpumalanga, Limpopo, Eastern Cape, Western Cape
114	*Cytisus scoparius* (L.) Link (= *Genista scoparia* (L.) Lam.)	Scotch broom	1a
115	*Datura ferox* L.	Large thorn apple	1b
116	*Datura innoxia* Mill.	Downy thorn apple	1b
117	*Datura stramonium* L.	Common thorn apple	1b
118	*Duchesnea indica* (Andrews) Focke	Wild strawberry	1b
119	*Duranta erecta* L. (= *D. repens* L., *D. plumieri* Jacq.)	Forget-me-not-tree, Pigeon berry	3
120	*Echinopsis spachiana* (Lem.) Friedrich & G.D.Rowley (= *Trichocereus spachianus* (Lem.) Riccob.)	Torch cactus	1b
121	*Echium plantagineum* L. (= *E. lycopsis* L.)	Patterson's curse	1b
122	*Echium vulgare* L.	Blue echium	1b
123	*Egeria densa* Planch. (= *Elodea densa* (Planch.) Casp.)	Dense water weed	1a
124	*Eichhornia crassipes* (Mart.) Solms	Water hyacinth	1b
125	*Elodea canadensis* Michx.	Canadian water weed	1a
126	*Elytrigia repens* (L.) Desv. ex Nevski (= *Agropyron repens* (L.) P. Beauv., *Elymus repens* (L.) Gould)	Couch grass	Category 1a on Prince Edward and Marion Islands
127	*Equisetum hyemale* L.	Rough horsetail, Common scouring-rush	1a
128	*Eriobotrya japonica* (Thunb.) Lindl.	Loquat	3
129	*Eucalyptus camaldulensis* Dehnh.	Red river gum	2
130	*Eucalyptus cladocalyx* F.Muell.	Sugar gum	2
131	*Eucalyptus conferruminata* D.J.Carr & S.G.M.Carr (*E. lehmannii* misapplied in South Africa)	Spider gum	1b
132	*Eucalyptus diversicolor* F.Muell.	Karri	2
133	*Eucalyptus globulus* Labill.	Blue gum, Tasmanian blue gum	5
134	*Eucalyptus gomphocephala* DC.	Tuart	5
135	*Eucalyptus grandis* W.Hill ex Maiden (*E. saligna* Sm. in part) and hybrids	Saligna gum, Rose gum	2
136	*Eucalyptus microcorys* F.Muell.	Tallow gum	5
137	*Eucalyptus paniculata* Sm.	Grey ironbark	5
138	*Eucalyptus sideroxylon* A.Cunn. ex Woolls	Black ironbark, Red ironbark	5
139	*Eugenia uniflora* L.	Pitanga, Surinam cherry	1a
140	*Euphorbia leucocephala* Lotsy	White poinsettia	5
141	*Euphorbia pulcherrima* Willd. ex Klotzsch	Poinsettia	5
142	*Fallopia sachalinensis* (F.Schmidt) Ronse Decr. (= *Polygonum sachalinense* F.Schmidt, *Reynoutria sachalinensis* (F.Schmidt) Nakai	Giant knotweed	1a
143	*Festuca rubra* L.	Creeping red fescue	Category 1a on Prince Edward and Marion Islands
144	*Flaveria bidentis* (L.) Kuntze	Smelter's-bush	5
145	*Foeniculum vulgare* Mill.	Fennel	5
146	*Fraxinus americana* L.	American ash	5
147	*Fraxinus angustifolia* Vahl	Algerian ash	5
148	*Genista monspessulana* (L.) L.A.S.Johnson (= *Cytisus monspessulanus* L., *C. candicans* (L.) DC.)	Montpellier broom	1a
149	*Gleditsia triacanthos* L. Excluding sterile cultivars	Honey locust	1b
150	*Grevillea banksii* R.Br.	Australian crimson oak, Red flowering silky oak	1a
151	*Grevillea robusta* A.Cunn. ex R.Br.	Australian silky oak	3
152	*Grevillea rosmarinifolia* A.Cunn.	Rosemary grevillea	5
153	*Hakea drupacea* (C.F.Gaertn.) Roem. & Schult. (= *H. suaveolens* R.Br.)	Sweet hakea	1a
154	*Hakea gibbosa* (Sm.) Cav.	Rock hakea	1b
155	*Hakea salicifolia* (Vent.) B.L.Burtt (= *H. saligna* Knight)	Willow hakea	5
156	*Hakea sericea* Schrad. & J.C.Wendl.	Silky hakea	1b
157	*Harrisia martinii* (Labour.) Britton & Rose	Moon cactus, Harrisia cactus	1b

	(= *Eriocereus martinii* (Labour.) Riccob.)		
158	*Hedera helix* L. subsp. *canariensis* (Willd.) Cout.	Canary ivy, Madeira ivy, Algerian ivy	5
159	*Hedera helix* L. subsp. *Helix*	English ivy	5
160	*Hedychium coccineum* Buch.-Ham. Ex Sm.	Red ginger lily	1a
161	*Hedychium coronarium* J.König	White ginger lily	1a
162	*Hedychium flavescens* Carey ex Roscoe	Yellow ginger lily	1a
163	*Hedychium gardnerianum* Sheppard ex Ker Gawl.	Kahili ginger lily	1a
164	*Houttuynia cordata* Thunb.	Chameleon plant	3
165	*Hydrocleys nymphoides* (Humb. & Bonpl. ex Willd.) Buchenau	Water poppy	1a
166	*Hylocereus undatus* (Haw.) Britton & Rose	Night-blooming cereus	1b
167	*Hypericum perforatum* L.	St. John's wort, Tipton weed	2
168	*Ipomoea alba* L.	Moonflower	1b
169	*Ipomoea carnea* Jacq. subsp. *fistulosa* (Mart. ex Choisy) D.F.Austin (= *I. fistulosa* Mart. ex Choisy)	Morning-glory bush	Category 1b in KwaZulu-Natal, Mpumalanga and Limpopo. Category 1a in rest of South Africa
170	*Ipomoea indica* (Burm.) Merr. (= *I. congesta* R.Br.)	Morning glory	1b
171	*Ipomoea purpurea* (L.) Roth	Morning glory	1b
172	*Iris pseudacorus* L.	Yellow flag	1a
173	*Jacaranda mimosifolia* D.Don	Jacaranda	Category 1b in KwaZulu-Natal, Mpumalanga and Limpopo. Category 2 in Gauteng, Category 3, Northern, Western and Eastern Cape. (For Category 2, this can be demarcated in areas broader than individual properties.)
174	*Jatropha curcas* L.	Physic nut	2
175	*Jatropha gossypiifolia* L.	Cotton-leaf physic nut	1a
176	*Jatropha multifida* L.	Coral plant	5
177	*Juniperus virginiana* L.	Red cedar	Category 3 in Free State and Eastern Cape
178	*Lablab purpureus* (L.) Sweet	Lablab bean	5
179	All seed producing species or seed producing hybrids of *Lantana* that are non-indigenous to Africa.	Lantana, Tickberry, Cherry pie	1b
180	*Lepidium draba* L. (= *Cardaria draba* (L.) Desv.)	Hoary cardaria	1b
181	*Leptospermum laevigatum* (Gaertn.) F.Muell.	Australian myrtle	1b
182	*Leptospermum scoparium* J.R.Forst. & G.Forst.	Manuka myrtle	5
183	*Leucaena leucocephala* (Lam.) de Wit (= *L. glauca* Benth.)	Leucaena	Category 1a in Western Cape. Category 2 in rest of South Africa
184	*Ligustrum japonicum* Thunb.	Japanese wax-leaved privet	Category 1b In KwaZulu-Natal, Mpumalanga, Limpopo, Eastern and Western Cape and Gauteng. Category 3 in Free State and North West
185	*Ligustrum lucidum* W.T.Aiton	Chinese wax-leaved privet	Category 1b In KwaZulu-Natal, Mpumalanga, Limpopo, Eastern and Western Cape and Gauteng. Category 3 in Free State and North West
186	*Ligustrum ovalifolium* Hassk.	Californian privet	Category 1b In KwaZulu-Natal, Mpumalanga, Limpopo, Eastern and Western Cape and Gauteng. Category 3 in Free State and North West
187	*Ligustrum sinense* Lour.	Chinese privet	Category 1b In KwaZulu-Natal, Mpumalanga, Limpopo, Eastern and Western Cape and Gauteng. Category 3 in Free State and North West
188	*Ligustrum vulgare* L.	Common privet	Category 1b In KwaZulu-Natal, Mpumalanga, Limpopo, Eastern and Western Cape and Gauteng. Category 3 in Free State and North West
189	*Lilium formosanum* Wallace (= *L. longiflorum* Thunb. var. *formosanum* Baker)	St Joseph's lily, Trumpet lily, Formosa lily	1a
190	*Litsea glutinosa* (Lour.) C.B.Rob. (= *Litsea sebifera* Pers.)	Indian laurel	1b
191	*Lolium multiflorum* Lam.	Italian ryegrass	5
192	*Lolium perenne* L.	Perennial ryegrass	5

193	*Lonicera japonica* Thunb. 'Halliana'	Japanese or Hall's honeysuckle	3
194	*Luzula sp.*	Woodrush	Category 1a on Prince Edward and Marion Islands
195	*Lythrum hyssopifolia* L.	Hyssop loosestrife	5
196	*Lythrum salicaria* L.	Purple loosestrife	1a
197	*Macfadyena unguis-cati* (L.) A.H.Gentry	Cat's claw creeper	1b
198	*Malva dendromorpha* M.F.Ray (= *Lavatera arborea* L.*)*	Tree mallow	5
199	*Malva verticillata* L.	Mallow	5
200	*Malvastrum coromandelianum* (L.) Garcke	Prickly malvastrum	5
201	*Melaleuca hypericifolia* Sm.	Red-flowering tea tree	1a
202	*Melia azedarach* L.	Seringa	Category 1b in KwaZulu-Natal, Mpumalanga and Limpopo. Category 3 elsewhere except in the Northern Cape with no category
203	*Melilotus indica* (L.) All.	Annual yellow sweet clover	5
204	*Metrosideros excelsa* Sol. ex Gaertn. (= *M. tomentosa* A.Rich.)	New Zealand Christmas tree	Category 1a in Overstrand District
205	*Mimosa pigra* L.	Giant sensitive plant	1b
206	*Mirabilis jalapa* L.	Four-o'clock, Marvel-of-Peru	1b
207	*Montanoa hibiscifolia* Benth.	Montanoa, Tree daisy	1b
208	*Moringa oleifera* Lam. (=*Moringa pterygosperma* Gaertn.)	Horse-radish tree	5
209	*Morus alba* L.	White mulberry, Common mulberry	2
210	*Morus nigra* L.	Black mulberry	5
211	*Murraya paniculata* (L.) Jack. (= *M. exotica* L.)	Orange Jessamine	Category 1a in KwaZulu-Natal, Mpumalanga, Limpopo and Eastern Cape. Category 1b for rest of country
212	*Myoporum* spp.	Manatoka trees	5
213	*Myoporum insulare* R.Br.	Manatoka, Boobyalla	5
214	*Myoporum laetum* G.Forst.	New Zealand manatoka	5
215	*Myoporum tenuifolium* G.Forst. subsp. *montanum* (R.Br.) Chinnock (= *M. montanum* R.Br.), (*M. acuminatum* misapplied in South Africa)	Manatoka	1b
216	*Myriophyllum aquaticum* (Vell.) Verdc.	Parrot's feather	1b
217	*Myriophyllum spicatum* L.	Spiked water-milfoil	1b
218	*Nassella tenuissima* (Trin.) Barkworth (= *Stipa tenuissima* Trin.)	White tussock	1b
219	*Nassella trichotoma* (Nees) Hack. ex Arechav. (= *Stipa trichotoma* Nees)	Nassella tussock	1b
220	*Nasturtium officinale* R.Br. (= *Rorippa nasturtium-aquaticum* (L.) Hayek)	Watercress	2
221	*Nephrolepis cordifolia* (L.) C.Presl (= *Polypodium cordifolium* L.)	Erect sword fern, Ladder sword fern	Category 1b in KwaZulu-Natal, Mpumalanga, Limpopo, Eastern and Western Cape. Category 3 in rest of South Africa
222	*Nephrolepis exaltata* (L.) Schott (= *Polypodium exaltatum* L.)	Sword fern, Boston sword fern	Category 1b in KwaZulu-Natal, Mpumalanga, Limpopo, Eastern and Western Cape. Category 3 in rest of South Africa
223	*Nerium oleander* L. Excluding sterile, double – flowered cultivars	Oleander	Category 1a in Eastern and Western Cape
224	*Nicandra physalodes* (L.) Gaertn.	Apple-of-Peru	5
225	*Nicotiana glauca* Graham	Wild tobacco	1b
226	*Nymphaea mexicana* Zucc. and hybrids	Yellow water lilies	1b
227	*Oenothera biennis* L.	Common evening-primrose	5
228	*Oenothera glazioviana* Micheli	Evening-primrose	5
229	*Oenothera indecora* Cambess.	Evening primrose	5
230	*Oenothera jamesii* Torr. & A.Gray	Giant evening-primrose	5
231	*Oenothera rosea* L´Hér. ex Aiton	Pink evening primrose	5
232	*Oenothera stricta* Ledeb. ex Link	Sweet sundrop	5
233	*Oenothera tetraptera* Cav.	White evening primrose	5
234	*Opuntia aurantiaca* Lindl.	Jointed cactus	1b
235	*Opuntia engelmannii* Salm-Dyck ex Engelm. (= *O. lindheimeri* Engelm.)	Small round-leaved prickly pear	1b
236	*Opuntia exaltata* A.Berger (= *Austrocylindropuntia exaltata* (A.Berger) Backeb.)	Long spine cactus	1a
237	*Opuntia ficus-indica* (L.) Mill. (= *O. megacantha* Salm-Dyck) Excluding all spineless cactus pear cultivars and selections	Mission prickly pear, Sweet prickly pear	1b
238	*Opuntia fulgida* Engelm.	Rosea cactus, Chain-fruit chollar	1b

	(*O. rosea* misapplied in South Africa)		
239	*Opuntia humifusa* (Raf.) Raf.<br>(*O. compressa* misapplied in South Africa)	Large flowered prickly pear, Creeping prickly pear	1b
240	*Opuntia imbricata* (Haw.) DC.<br>(= *Cylindropuntia imbricata* (Haw.) F.M.Knuth)	Imbricate cactus, Imbricate prickly pear	1b
241	*Opuntia microdasys* (Lehm.) Pfeiff.	Yellow bunny-ears, Teddy-bear cactus	1a
242	*Opuntia monacantha* Haw.<br>(*O. vulgaris* misapplied in South Africa)	Cochineal prickly pear, Drooping prickly pear	1b
243	*Opuntia robusta* H.L.Wendl. ex Pfeiff.	Blue-leaf cactus	2
244	*Opuntia spinulifera* Salm-Dyck	Saucepan cactus, Large roundleaved prickly pear	1a
245	*Opuntia stricta* (Haw.) Haw.<br>(= *O. dillennii* (Ker Gawl.) Haw.)	Pest pear of Australia	1b
246	*Orobanche minor* Sm.	Lesser broomrape, Clover broomrape	1b
247	*Orobanche ramosa* L.	Blue broomrape, Branched broomrape	1b
248	*Paraserianthes lophantha* (Willd.) I.C.Nielsen<br>(= *Albizia lophantha* (Willd.) Benth.)	Australian albizia, Stink bean	1b
249	*Parkinsonia aculeata* L.	Jerusalem thorn	Category 5 in North West and Northern Cape
250	*Parthenium hysterophorus* L.	Parthenium	1b
251	*Paspalum dilatatum* Poir.	Common paspalum	5
252	*Paspalum quadrifarium* Lam.		1a
253	*Paspalum urvillei* Steud.	Tall paspalum	5
254	*Passiflora caerulea* L.	Blue passion flower	1b
255	*Passiflora edulis* Sims	Purple granadilla, Passion fruit	Category 2 in KwaZulu-Natal, Mpumalanga, Limpopo and Eatern Cape.
256	*Passiflora tripartita* (Juss.) Poir. var. *mollissima* (Kunth) Holm-Niels. & P.Jorg.<br>(= *P. mollissima* (Kunth) L.H.Bailey	Banana poka, Bananadilla	1b
257	*Passiflora suberosa* L.	Devil’s pumpkin, Indigo berry	1b
258	*Passiflora subpeltata* Ortega	Granadina	1a
259	*Paulownia tomentosa* (Thunb.) Steud.<br>(=*Paulownia imperialis* Siebold & Zucc.)	Empress tree, Princess tree, Royal Paulownia	1b
260	*Pennisetum clandestinum* Hochst. ex Chiov.	Kikuyu grass	2 (This can be demarcated in areas broader than individual properties)
261	*Pennisetum purpureum* Schumach.	Elephant grass, Napier grass	1b
262	*Pennisetum setaceum* (Forssk.) Chiov. Excluding sterile cultivar ‘Rubrum’	Fountain grass	1b
263	*Pennisetum villosum* R.Br. ex Fresen.	Feathertop	1b
264	*Pereskia aculeata* Mill.	Pereskia, Barbados gooseberry	1b
265	*Persicaria capitata* (Buch.-Ham. ex D.Don) H.Gross (= *Polygonum capitatum* Buch.-Ham. ex D.Don*)*	Knotweed	1a
266	*Persicaria limbata* (Meisn.) H.Hara<br>(= *Polygonum limbatum* Meisn.)	Knotweed	5
267	*Phytolacca dioica* L.	Belhambra	3
268	*Phytolacca icosandra* L.<br>(= *P. octandra* L.*)*	Forest inkberry	1b
269	*Pinus canariensis* C.Sm.	Canary pine	3
270	*Pinus elliotti* Engelm.	Slash pine	2
271	*Pinus halepensis* Mill.	Aleppo pine	2
272	*Pinus patula* Schiede ex Schltdl. & Cham.	Patula pine	2
273	*Pinus pinaster* Aiton	Cluster pine	2
274	*Pinus pinea* L.	Umbrella or Stone pine	5
275	*Pinus radiata* D.Don	Radiata pine, Monterey pine	2
276	*Pinus roxburghii* Sarg.<br>(= *P. longifolia* Roxb. ex Lamb.)	Chir pine, Longifolia pine	2
277	*Pinus taeda* L.	Loblolly pine	2
278	*Pistia stratiotes* L.	Water lettuce	1a
279	*Pittosporum crassifolium* Banks & Sol. ex A.Cunn.	Karo, Stiff-leaved cheesewood	3
280	*Pittosporum undulatum* Vent.	Australian cheesewood, Sweet pittosporum	1a
281	*Plectranthus comosus* Sims<br>(= *Coleus grandis* Cramer)<br>*(Plectranthus barbatus* misapplied in South Africa)	‘Abyssinian’ coleus, Woolly plectranthus	1b
282	*Poa pratensis* L.	Kentucky bluegrass	Category 1a on Prince Edward Island. Category 1b on Marion Island

283	*Polypodium aureum* (L.) J.Sm.	Rabbits-foot fern	Category 3 in KwaZulu-Natal, Mpumalanga, Limpopo and Eastern Cape
284	*Pontederia cordata* L.	Pickerel weed	1a
285	*Populus alba* L.	White poplar	2
286	*Populus deltoides* W.Bartram ex Marshall subsp. *deltoides*	Match poplar	5
287	*Populus deltoides* W.Bartram ex Marshall subsp. *wislizeni* (S.Watson) Eckenw. (= *P. wislizeni* (S.Watson) Sarg.)	Valley match poplar	5
288	*Populus nigra* L. var. *italica* Münchh.	Lombardy poplar	5
289	*Populus ×canescens* (Aiton) Sm.	Grey poplar, Matchwood poplar	2
290	*Prosopis glandulosa* Torr. var. *torreyana* (Benson) Johnst. and hybrids	Honey mesquite	Category 1b in North West, Free State, Eastern and Western Cape. Category 2 in Northern Cape
291	*Prosopis velutina* Wooton and hybrids	Velvet mesquite	Category 1b in North West, Free State, Eastern and Western Cape, Category 2 in Northern Cape
292	*Protea aurea* and all derived fertile hybrids	Long-bud protea	Category 4<br>Not permitted outside of natural range in the Western Cape, Eastern Cape and KwaZulu-Natal within 5 km of natural populations of other members of White Water Proteas (*P. lacticolor, P. mundii, P. punctata, P. subvestita*)
293	*Protea lacticolor* and all derived fertile hybrids	Hottentot white protea	Category 4<br>Not permitted outside of natural range in the Western Cape, Eastern Cape and KwaZulu-Natal within 5 km of natural populations of other members of White Water Proteas (*P. aurea, P. mundii, P. punctata, P. subvestita*)
294	*Protea longifolia* and all derived fertile hybrids		Category 4<br>Not permitted within the Western and Eastern Cape outside of its natural range (i.e. Fynbos outside of Kogelberg and Overberg Sandstone Fynbos)
295	*Protea mundii* and all derived fertile hybrids	White protea	Category 4<br>Not permitted outside of natural range in the Western Cape, Eastern Cape and KwaZulu-Natal within 5 km of natural populations of other members of White Water Proteas (*P. aurea, P. lacticolor, P. punctata, P. subvestita*)
296	*Protea neriifolia*	Oleander - leaved protea	Category 4<br>Not permitted within the distribution range of *Protea lepidocarpodendron* or 1 km thereof. (Cape Peninsula, Helderberg, coastal towns and mountains from Pringle Bay to Stanford)
297	*Protea punctata* and all derived fertile hybrids	Water white sugarbush	Category 4<br>Not permitted outside of natural range in the Western Cape, Eastern Cape and KwaZulu-Natal within 5 km of natural populations of other members of White Water Proteas (*P. aurea, P. lacticolor, P. mundii, P. subvestita*)
298	*Protea subvestita* and all derived fertile hybrids	Lipped protea	Category 4<br>Not permitted outside of natural range in the Western Cape and Eastern Cape within 5 km of natural populations of other members of White Water Proteas (*P. aurea, P. lacticolor, P. mundii, P. punctata*).
299	*Protea susannae* and all derived fertile hybrids	Stinkleaf protea	Category 4<br>Not permitted within the Western and Eastern Cape outside of its natural range (i.e. Fynbos outside of Agulhas Plain, and Agulhas, De Hoop & Canca Limestone Fynnos)
300	*Prunus cerasifera* Ehrh.	Cherry plum	5
301	*Prunus serotina* Ehrh.	Black cherry	3
302	*Psidium cattleianum* Sabine (= *P. littorale* Raddi var. *longipes* (O.Berg.) Fosberg	Strawberry guava	1b
303	*Psidium guajava* L. and hybrids	Guava	Category 2 in KwaZulu-Natal,

			Mpumalanga, Limpopo and the Eastern Cape
304	*Psidium guineense* Sw.	Brazilian guava	1a
305	*Psidium ×durbanensis* Baijnath ined.	Durban guava	1a
306	*Pterocarya fraxinifolia* (Lam. ex Poir.) Spach.	Caucasian wing nut	5
307	*Pueraria montana* (Lour.) Merr. var. *lobata* (Willd.) Maesen & S.M.Almeida (= *P. lobata* (Willd.) Ohwi)	Kudzu vine	1a
308	*Pyracantha angustifolia* (Franch.) C.K.Schneid.	Yellow firethorn	1b
309	*Pyracantha coccinea* M.Roem.	Red firethorn	5
310	*Pyracantha crenatoserrata* (Hance) Rehder (= *P. fortuneana* misapplied)	Chinese firethorn, Broad leaf firethorn	5
311	*Pyracantha crenulata* (D.Don) M.Roem.	Himalayan firethorn	1b
312	*Pyracantha koidzumii* (Hayata) Rehder	Formosa firethorn	5
313	*Pyracantha rogersiana* (A.B.Jacks.) Chitt.	Firethorn	5
314	*Quercus acutissima* Carruth.	Bristle oak	5
315	*Raphanus raphanistrum* L.	Wild radish	5
316	*Rhus glabra* L.	Scarlet sumach, Vinegar bush	3
317	*Ricinus communis* L.	Castor-oil plant	2
318	*Rivina humilis* L.	Rivina, Bloodberry	1a
319	*Robinia pseudoacacia* L.	Black locust	1b
320	*Rosa canina* L.	Dog-rose	5
321	*Rosa rubiginosa* L. (= *R. eglanteria* L.)	Eglantine, Sweetbriar	1b
322	*Rubus cuneifolius* Pursh and hybrid *R. ×proteus* C.H.Stirt.	American bramble	1b
323	*Rubus flagellaris* Willd.	Bramble	1a
324	*Rubus fruticosus* L. agg.	European blackberry	2
325	*Rubus niveus* Thunb.	Ceylon raspberry, Mysore raspberry	1b
326	*Rumex acetosella* L.	Sheep sorrel, Red sorrel	Category 1a on Prince Edward and Marion Islands
327	*Rumex crispus* L.	Curly dock	5
328	*Salix babylonica* L.	Weeping willow	3
329	*Salix fragilis* L.	Crack or Brittle willow	1b
330	*Salsola kali* L.	Tumbleweed	1b
331	*Salsola tragus* L. (= *S. australis* R.Br.)	Russian tumbleweed	1b
332	*Salvia tiliifolia* Vahl	Lindenleaf sage	1b
333	*Salvinia molesta* D.S.Mitch. and other species of the Family Salviniaceae	Kariba weed, Salvinia	1a
334	*Sambucus canadensis* L. (= *S. nigra* L. subsp. *canadensis* (L.) Bolli	Canadian elder	1b
335	*Sambucus nigra* L.	European elder	1b
336	*Sasa ramosa* (Makino) Makino & Shibata (= *Arundinaria vagans* Gamble)	Dwarf yellow-striped bamboo	3
337	*Schefflera actinophylla* (Endl.) Harms	Australian cabbage tree, Queensland umbrella tree	Category 1b in KwaZulu-Natal, Mpumalanga, Limpopo and Eastern Cape
338	*Schefflera arboricola* (Hayata) Merr.	Dwarf umbrella tree	Category 3 in KwaZulu-Natal, Mpumalanga, Limpopo and Eastern Cape
339	*Schefflera elegantissima* (hort. Veitch ex Mast.) Lowry & Frodin (= *Dizygotheca elegantissima* (hort. Veitch ex Mast.) R.Vig. & Guillaumin	False aralia	5
340	*Schinus molle* L.	Pepper tree	5
341	*Schinus terebinthifolius* Raddi	Brazilian pepper tree	1b
342	*Senna bicapsularis* (L.) Roxb. (= *Cassia bicapsularis* L.)	Rambling cassia	1b
343	*Senna didymobotrya* (Fresen.) H.S.Irwin & Barneby (= *Cassia didymobotrya* Fresen.)	Peanut butter cassia	Category 1b in KwaZulu-Natal, Mpumalanga, Limpopo and Eastern Cape. Category 3 in the rest of South Africa
344	*Senna hirsuta* (L.) H.S.Irwin & Barneby (= *Cassia hirsuta* L.)		1b
345	*Senna occidentalis* (L.) Link (= *Cassia occidentalis* L.)	Stinking weed, Wild coffee	1b
346	*Senna pendula* (Willd.) H.S.Irwin & Barneby var. *glabrata* (Vogel) H.S.Irwin & Barneby (= *Cassia coluteoides* Collad.)		1b
347	*Senna septemtrionalis* (Viv.) H.S.Irwin & Barneby (= *Cassia floribunda* sensu Brenan non	Arsenic bush, Smooth senna	1b

	Cav., *C. laevigata* Willd.)		
348	*Sesbania punicea* (Cav.) Benth.	Red sesbania	1b
349	*Solanum betaceum* Cav.<br>(= *Cyphomandra betacea* (Cav.) Sendtn.)	Tree tomato	5
350	*Solanum chrysotrichum* Schltdl.<br>(*S. hispidum* misapplied in South Africa)	Giant devil's fig	1b
351	*Solanum elaeagnifolium* Cav.	Silver-leaf bitter apple	1b
352	*Solanum mauritianum* Scop.	Bugweed	1b
353	*Solanum pseudocapsicum* L.	Jerusalem cherry	Category 1a in coastal areas, Mpumalanga and Limpopo. Category 1b in rest of South Africa
354	*Solanum seaforthianum* Andrews	Potato creeper	1b
355	*Solanum sisymbriifolium* Lam.	Wild tomato, Dense-thorned bitter apple	1b
356	*Sorghum halepense* (L.) Pers.	Johnson grass, Aleppo grass	2
357	*Spartium junceum* L.	Spanish broom	Category 1a in Western Cape
358	*Spathodea campanulata* P.Beauv.	African flame tree	Category 3 in KwaZulu-Natal, Mpumalanga, Limpopo and Eastern Cape
359	*Sphagneticola trilobata* (L.) Pruski<br>(= *Thelechitonia trilobata* (L.) H.Rob. & Cuatrec., *Wedelia trilobata* (L.) Hitchc.)	Singapore daisy	Category 1b in KwaZulu-Natal, Mpumalanga, Limpopo. Category 3 in rest of South Africa
360	*Stachytarpheta* spp.	Snakeweeds	3
361	*Stellaria media* (L.) Vill.	Common chickweed	Category 1a on Prince Edward Island and 1b on Marion Island
362	*Syncarpia glomulifera* (Sm.) Nied.	Turpentine tree	5
363	*Syngonium* spp.	Goose foot plants, Arrow-head vines	Category 1a in KwaZulu-Natal, Mpumalanga, Limpopo and Eastern Cape
364	*Syzygium cumini* (L.) Skeels	Jambolan	Category 1b in KwaZulu-Natal, Mpumalanga, Limpopo. Category 1b in the rest of South Africa
365	*Syzygium jambos* (L.) Alston	Rose apple	3
366	*Syzygium paniculatum* Gaertn.<br>(= *Eugenia myrtifolia* Sims)	Australian water-pear, Australian brush-cherry	5
367	*Tamarix aphylla* (L.) H.Karst.<br>Not to be confused with indigenous *Tamarix usneoides*	Athel tree, Desert tamarisk	1b
368	*Tamarix chinensis* Lour.<br>Not to be confused with indigenous *Tamarix usneoides*	Chinese tamarisk	1b
369	*Tamarix gallica* L.<br>Not to be confused with indigenous *Tamarix usneoides*	French tamarisk	1b
370	*Tamarix ramosissima* Ledeb.<br>Not to be confused with indigenous *Tamarix usneoides*	Pink tamarisk	1b
371	*Tecoma stans* (L.) Juss. ex Kunth	Yellow bells	1b
372	*Thevetia peruviana* (Pers.) K.Schum.<br>(= *T. neriifolia* Juss. ex Steud.)	Yellow oleander	1b
373	*Tipuana tipu* (Benth.) Kuntze<br>(= *T. speciosa* Benth.)	Tipu tree	3
374	*Tithonia diversifolia* (Hemsl.) A.Gray	Mexican sunflower	1b
375	*Tithonia rotundifolia* (Mill.) S.F.Blake	Red sunflower	1b
376	*Toona ciliata* M.Roem.<br>(= *Cedrela toona* Roxb. ex Willd.)	Toon tree	3
377	*Toxicodendron succedaneum* (L.) Kuntze<br>(= *Rhus succedanea* L.)	Wax tree	1a
378	*Tradescantia fluminensis* Vell.	Wandering Jew	1a
379	*Tradescantia pallida* (Rose) D.R.Hunt<br>(= *Setcreasea purpurea* Boom)	Purpleheart	5
380	*Tradescantia spathacea* Sw.<br>(= *Rhoeo spathacea* (Sw.) Stearn)	Boat lily	5
381	*Tradescantia zebrina* hort. ex Bosse<br>(= *Zebrina pendula* Schnizl.)	Wandering Jew	1a
382	*Triplaris americana* L.	Triplaris, Ant tree	Category 1a in KwaZulu-Natal, Mpumalanga, Limpopo and Eastern Cape. Category 5 in rest of South Africa
383	*Ulex europaeus* L.	European gorse	1b
384	*Ulmus parvifiolia* Jacq.<br>(= *U. chinensis* Pers.)	Chinese elm	5
385	*Verbena bonariensis* L.	Wild verbena, Tall verbena, Purple top	1b
386	*Verbena brasiliensis* Vell.	Brazilian verbena	1b
387	*Vinca major* L.	Greater periwinkle	5
388	*Vinca minor* L.	Lesser periwinkle	5

389	*Vitex trifolia* L.	Indian three-leaf vitex	Category 1a in KwaZulu-Natal, Mpumalanga, Limpopo and Eastern Cape
390	*Wigandia urens* (Ruiz & Pav.) Kunth var. *caracasana* (Kunth) D.N.Gibson (= *W. caracasana* Kunth)	Wigandia	5
391	*Xanthium spinosum* L.	Spiny cocklebur	1b
392	*Xanthium strumarium* L.	Large cocklebur	1b

3.10 Microbes

Still under discussion.

Appendix 4

Species Listed as Known to be Invasive Elsewhere in the World

4.1 Mammals

No species listed at present.

4.2 Birds

No species listed at present.

4.3 Reptiles

No species listed at present.

4.4 Amphibians

No	Species	Common Name
1	*Xenopus laevis*	African clawed frog

4.5 Fishes (Fresh-water)

No	Species	Common name
1	*Labeobarbus aeneus*	Vaal-Orange smallmouth yellowfish
2	*Labeobarbus capensis*	Clanwilliam yellowfish
3	*Labeobarbus marequensis*	Lowveld largescale yellowfish
4	*Labeobarbus natalensis*	KwaZulu-Natal yellowfish
5	*Labeobarbus polylepis*	Bushveld smallscale yellowfish
6	*Labeo umbratus*	Moggel
7	*Clarias gariepinus*	sharptooth catfish
8	*Clarias ngamensis*	blunttooth catfish
9	*Chetia brevis*	orange-fringed largemouth
10	*Chetia flaviventris*	canary kurper
11	*Oreochromis mossambicus*	Mocambique tilapia
12	*Oreochromis placidus*	black tilapia
13	*Pseudocrenilabrus philander*	southern mouthbrooder
14	*Serranochromis meridianus*	lowveld largemouth
15	*Tilapia rendalli*	redbreast tilapia
16	*Tilapia sparrmanii*	banded tilapia

4.6 Fishes (Marine) and Other Marine Species

Still under discussion.

4.7 Invertebrates (Fresh-water)

Still under discussion.

4.8 Invertebrates (Terrestrial)

Still under discussion.

4.9 Plants

No	Species	Common Name
1	*Acacia erioloba* E.Mey.	Camel thorn
2	*Acacia karroo* Hayne	Sweet thorn
3	*Acacia nilotica* (L.) Delile	Scented thorn
4	*Alectra* spp.	Witchweeds
5	*Arctotheca calendula* (L.) Levyns	Cape marigold
6	*Aristea ecklonii* Baker	Blue stars
7	*Aristolochia* spp.	Calico flowers
8	*Asparagus africanus* Lam.	Haakdoring
9	*Asparagus asparagoides* (L.) Druce	Cape smilax, Bridal-creeper
10	*Asparagus declinatus* L.	Bridal-veil
11	*Asparagus densiflorus* (Kunth) Jessop	Foxtail fern, Cat's tail asparagus
12	*Asparagus scandens* Thunb.	Climbing asparagus, Ferny asparagus
13	*Asparagus setaceus* (Kunth) Jessop	Asparagus-fern
14	*Barleria prionitoides* Engl.	Barleria, Porcupine flower
15	*Berkheya rigida* (Thunb.) Ewart et al.	African thistle
16	*Ceratophyllum demersum* L.	Water hornwort
17	*Chrysanthemoides monilifera* (L.) Norl	Bietou / Boneseed
18	*Cineraria lyratiformis* Cron	African marigold, Wild Parsley
19	*Commelina benghalensis* L.	Benghal wandering Jew
20	*Crocosmia* X *crocosmiiflora* (Lemoine) N.E.Br.	Autumn-gold, Garden montbretia
21	*Cucumis myriocarpus* Naudin	Striped wild cucumber
22	*Cuscuta* spp.	Dodders
23	*Cynodon* spp.	Couch-grasses, Quick grasses
24	*Cyperus esculentus* L.	Yellow nutsedge
25	*Cyperus rotundus* L.	Purple nutsedge
26	*Delairea odorata* Lem.	Cape ivy, Ivy groundsel
27	*Dichrostachys cinerea* (L.) Wight & Arn.	Sickle bush
28	*Digitaria scalarum (Schweinf.) Chiov.*	African finger grass
29	*Digitaria velutina* (Forssk.) P.Beauv.	Long-plumed finger-grass
30	*Dipogon lignosus* (L.) Verdc.	Wild bean
31	*Ehrharta erecta* Lam.	Panic veld grass
32	*Ehrharta villosa* Schult.f.	Pipe grass
33	*Emex australis* Steinh.	Spiny emex
34	*Eragrostis curvula* (Schrad.) Nees	African love grass
35	*Galenia pubescens* (Eckl. & Zeyh.) Druce	Bloubrakbossie
36	*Galium spurium* L.	Catchweed
37	*Gloriosa superba* L.	Flame-lily, Glory-lily
38	*Gomphocarpus fruticosus* (L.) W.T.Aiton	Milkweed
39	*Heteropogon contortus* (L.) P.Beauv. ex Roem. & Schult.	Common spear grass
40	*Hydrocotyle verticillata* Thunb.	Hydrocotyle, Shield pennywort
41	*Imperata cylindrica* (L.) P.Beauv.	Silver spike
42	*Ipomoea aquatica* Forssk.	Swamp morning-glory
43	*Lagarosiphon major* (Ridl.) Moss	African oxygen weed
44	*Leonotis nepetifolia* (L.) R.Br.	Lion's tail
45	*Leptochloa chinensis* (L.) Nees	Red sprangletop
46	*Limnophila indica* (L.) Druce	Ambulia
47	*Lycium ferocissimum* Miers	African box thorn
48	*Lygodium microphyllum* (Cav.) R.Br.	Climbing maidenhair fern
49	*Melianthus comosus* Vahl	Tufted honey flower
50	*Melianthus major* L.	Large honey flower
51	*Mikania* spp.	Mikania vines
52	*Mimosa pigra* L.	Giant sensitive plant
53	*Moraea* spp. (= *Homeria* spp.)	Cape tulips
54	*Najas marina* L.	Sawtooth, Spiny naiad
55	*Ochna serrulata* (Hochst.) Walp.	Small-leaved plane
56	*Olea europaea* (Mill.) P.S.Green subsp. *africana*	Olive
57	*Oncosiphon suffruticosum* (L.) Källersjö	Karoobush
58	*Oryza longistaminata* A.Chev. & Roehr.	Red rice
59	*Oryza punctata* Kotschy ex Steud.	Red rice
60	*Osmunda regalis* L.	Royal fern
61	*Oxalis pes-caprae* L.	Yellow sorrel
62	*Panicum repens* L.	Couch panicum
63	*Paspalum scrobiculatum* L.	Creeping paspalum, water couch
64	*Pennisetum macrourum* Trin.	African feathergrass
65	*Phragmites australis* (Cav.) Trin. ex Steud.	Common reed
66	*Phyla* spp.	Daisylawn, Capeweed
67	*Plectranthus ciliatus* E.Mey.	Speckled spurflower
68	*Plectranthus ecklonii* Benth.	Purple spurflower
69	*Polygala myrtifolia* L.	September bush
70	*Rottboellia cochinchensis* (Lour.) Clayton	Guinea-fowl grass
71	*Rumex saggitatus* Thunb.	Red sorrel

72	*Selaginella kraussiana* (Kunze) A.Braun	Mat spike-moss
73	*Senecio angulatus* L.f.	Cape ivy
74	*Senecio madagascariensis* Poir.	Fireweed
75	*Senecio pterophorus* DC.	African daisy
76	*Setaria pumila* (Poir.) Roem. & Schult.	Pigeon grass, Yellow foxtail
77	*Setaria verticillata* (L.) P.Beauv.	Sticky bristle grass
78	*Sida acuta* Burm.f.	Broomweed, Spiny-head sida
79	*Sida cordifolia* L.	Heartleaf sida
80	*Sida rhombifolia* L.	Arrowleaf sida
81	*Silene vulgaris* (Moench) Garcke	Bladder campion, Maiden's-tears
82	*Solanum linneanum* Hepper & Jaeger	Bitter apple, Apple-of-Sodom
83	*Sporobolus africanus* (Poir.) Robyns & Tournay	Rat's tail grass
84	*Sporobolus natalensis* (Steud.) T.Durand & Schinz	Giant rat's tail grass
85	*Sporobolus pyramidalis* P.Beauv.	Cat's tail grass
86	*Striga asiatica* (L.) Kuntze	Common mealie-witchweed
87	*Trapa* spp.	Water chestnuts
88	*Tribulus terrestris* L.	Devil's-thorn
89	*Triumfetta rhomboidea* Jacq.	Chinese bur
90	*Typha capensis* (Rohrb.) N.E.Br.	Common bulrush
91	*Typha domingensis* Pers. (= *T. australis* Schumach.)	Bulrush, Cattail
92	*Utricularia gibba* L.	Bladderwort
93	*Urochloa panicoides* P.Beauv.	Beesgras
94	*Vossia cuspidata* (Roxb.) Griff.	Hippo grass
95	*Watsonia meriana* (L.) Mill.	Dwarf red watsonia
96	*Zantedeschia aethiopica* (L.) Spreng.	White arum lily

4.10 Microbes

Still under discussion.

Appendix 5

Species Listed as Potentially Invasive Elsewhere in the World

Seeds and other propagules of the following species must be labelled, informing people acquiring them that the species may be or may become invasive elsewhere in the world.

5.1 Mammals

No	Species	Common Name
1	*Mastomys natalensis*	Natal multimammate mouse

5.2 Birds

No species listed at present.

5.3 Reptiles

No species listed at present.

5.4 Amphibians

No species listed at present.

5.5 Fishes (Fresh-water)

No	Species	Common name
1	*Labeobarbus kimberleyensis*	Vaal-Orange largemouth yellowfish
2	*Hydrocynus vittatus*	Tigerfish
3	*Clarias theodorae*	snake catfish

5.6 Fishes (Marine) and Other Marine Species

Still under discussion.

5.7 Invertebrates (Fresh-water)

Still under discussion.

5.8 Invertebrates (Marine)

Still under discussion.

5.9 Plants

No	Species	Common Name
1	*Agapanthus praecox* Willd. subsp. *orientalis* (F. M. Leight.) F. M. Leight.	Common agapanthus
2	*Aloe maculata* All. (= *A. saponaria* (Aiton) Haw.)	Soap aloe, Spotted aloe
3	*Amaryllis belladonna* L.	Belladonna lily
4	*Anchusa capensis* Thunb.	Cape forget-me-not
5	*Aponogeton distachyos* L.f.	Cape pondweed

6	*Aptenia cordifolia* (L.f.) Schwantes	Aptenia
7	*Arctotheca populifolia* (P.J.Bergius) Norl.	Seepampoen
8	*Arctotis stoechadifolia* P.J.Bergius	Trailing arctotis
9	*Arctotis venusta* Norl.	Free State daisy
10	*Babiana stricta* (Aiton) Ker Gawl.	Babiana
11	*Babiana disticha* Ker Gawl.	Blue babiana
12	*Babiana tubulosa* (Burm.f.) Ker Gawl.	Babiana
13	*Baeometra uniflora* (Jacq.) G.J.Lewis	
14	*Bauhinia galpinii* N.E.Br.	Pride-of-De Kaap
15	*Buddleja saligna* Willd.	False olive
16	*Buddleja salviifolia* (L.) Lam.	Sagewood
17	*Carpobrotus acinaciformis* (L.) L.Bolus	Sour fig
18	*Carpobrotus edulis* (L.) L.Bolus	Yellow sour-fig, Hottentot fig
19	*Chasmanthe floribunda* (Salisb.) N.E.Br.	Flames, Pennants
20	*Chloris gayana* Kunth	Hunyani grass, Rhodes grass
21	*Chloris virgata* Sw.	Feathertop chloris
22	*Chlorophytum comosum* (Thunb.) Jacq.	Hen-and-chickens
23	*Citrillus lanatus* (Thunb.) Matsum. & Nakai	Wild melon
24	*Cleretum papulosum* (L.f.) L.Bolus (= *Micropterum papulosum* (L.f.) Schwantes	
25	*Conicosia pugioniformis* (L.) N.E.Br.	
26	*Cotula bipinnata* Thunb.	
27	*Cotula turbinata* L.	Goose daisy
28	*Cotyledon orbiculata* L.	Pig’s ear
29	*Crassula* spp. and hybrids	Crassulas
30	*Cyanella hyacinthoides* L.	Lady’s-hand
31	*Dactyloctenium australe* Steud.	Durban grass, LM grass
32	*Delosperma litorale* (Kensit) L.Bolus	Klipvygie
33	*Dietes iridioides* (L.) Sweet ex Klatt (= *D. vegeta* (L.) N.E.Br.	Wild iris
34	*Dimorphotheca ecklonis* DC.	Blue-and-white daisy bush, Sunday’s River daisy
35	*Dimorphotheca fruticosa* (L.) Less. (= *Osteospermum fruticosum* (L.) Norl.	Creeping marguerite
36	*Dimorphotheca jucunda* E.Phillips (= *Osteospermum jucundum* (E.Phillips) Norl.	Mountain bietou, Trailing mauve daisy
37	*Dimorphotheca sinuata* DC.	Namaqualand daisy
38	*Disa bracteata* Sw. (= *Monadenia bracteata* (Sw.) T.Durand & Schinz	
39	*Dischisma arenarium* E.Mey.	
40	*Dischisma capitatum* (Thunb.) Choisy	
41	*Ehrharta brevifolia* Schrad.	
42	*Ehrharta calycina* Sm.	Veld grass
43	*Ehrharta longiflora* Sm.	
44	*Erica baccans* L.	Berry heath
45	*Euryops abrotanifolius* (L.) DC.	Yellow marguerite
46	*Euryops subcarnosus* DC.	
47	*Ferraria crispa* Burm.	Spider flower
48	*Freesia* spp. and hybrids	Freesias
49	*Garuleum latifolium* Harv. (= *Osteospermum calendulaceum* Harv.)	
50	*Gazania linearis* (Thunb.) Druce	Gazania
51	*Gazania rigens* (L.) Gaertn.	Trailing gazania
52	*Gladiolus* spp. and hybrids	Gladioli
53	*Gomphocarpus physocarpus* E.Mey.	Wild cotton
54	*Gorteria personata* L.	
55	*Grammototheca bergiana* (Cham.) C.Presl	
56	*Helichrysum petiolare* Hilliard & B.L.Burtt	Curry bush
57	*Heliophila pusilla* L.f.	
58	*Hesperantha coccinea* (Backh. & Harv.) Goldblatt & J.C.Manning (= *Schizostylis coccinea* Backh. & Harv.)	Scarlet river lily
59	*Hesperantha falcata* (L.f.) Ker Gawl.	Evening flower
60	*Holcus setiger* Nees	
61	*Ixia maculata* L.	Yellow ixia
62	*Ixia paniculata* D.Delaroche	
63	*Ixia polystachya* L.	Corn lily
64	*Ixia viridiflora* Lam.	Green ixia
65	*Juncus oxycarpus* E.Mey. ex Kunth	Rush
66	*Lachenalia aloides* (L.f.) Engl.	Golden lachenalia
67	*Lachenalia bulbifera* (Cirillo) Engl.	Red lachenalia
68	*Lachenalia mutabilis* Sweet	
69	*Lachenalia reflexa* Thunb.	
70	*Lampranthus* spp. and hybrids	Vygies
71	*Leonotis leonurus* (L.) R.Br.	Wild dagga
72	*Lobelia spp.* and hybrids	Lobelias
73	*Malephora crocea* (Jacq.) Schwantes	Vingerkanna
74	*Melinus repens* (Willd.) Zizka	Natal red-top

75	*Mesembryanthemum aitonis* Jacq.	Mesem, Ice plant
76	*Mesembryanthemum crystallinum* L.	Mesem, Ice plant
77	*Mesembryanthemum nodiflorum* L.	Mesem, Ice plant
78	*Monopsis debilis* (L.f.) C.Presl	
79	*Muraltia heisteria* (L.) DC.	
80	*Nemesia floribunda* Lehm.	Leeubekkie
81	*Nemesia strumosa* (Herb. Banks ex Benth.) Benth.	Cape jewels
82	*Ornithogalum maximum* (Burm. f.) J.C. Manning & Goldblatt (= *Albuca canadensis* (L.) F.M.Leight.)	
83	*Ornithogalum thyrsoides* Jacq.	Chinkerinchee
84	*Oxalis compressa* L.f.	Sorrel
85	*Oxalis flava* L.	Yellow oxalis
86	*Oxalis glabra* Thunb.	Sorrel
87	*Oxalis incarnata* L.	Sorrel
88	*Oxalis purpurea* L.	Sorrel
89	*Pelargonium* spp. and hybrids	Pelargoniums
90	*Pentaschistis airoides* (Nees) Stapf	Haasgras
91	*Pentaschistis pallida* (Thunb.) H.P.Linder	Duingras, Haasgras
92	*Pentzia globosa* Less.	Globe flower
93	*Phygelius capensis* E.Mey. ex Benth.	Cape fuchsia, River bells
94	*Phyllopodium cordatum* (Thunb.) Hilliard	
95	*Pittosporum viridiflorum* Sims	Cheesewood
96	*Podalyria sericea* (Andrews) R.Br. ex W.T.Aiton	Silver water blossom pea
97	*Polygala virgata* Thunb.	Purple broom
98	*Psoralea pinnata* L.	Fountain bush
99	*Romulea flava* (Lam.) M.P.de Vos	
100	*Romulea minutiflora* Klatt	Frutang
101	*Romulea obscura* Klatt	Frutang
102	*Romulea rosea* (L.) Eckl.	Frutang, Pink romulea, Onion grass
103	*Senecio burchellii* DC.	Molteno disease plant
104	*Senecio elegans* L.	Purple senecio
105	*Senecio glastifolius* L.f.	Holly leaved senecio
106	*Senecio tamoides* DC.	Canary creeper
107	*Sparaxis bulbifera* (L.) Ker Gawl.	Dwarf sparaxis
108	*Sparaxis grandiflora* (D.Delaroche) Ker Gawl.	White sparaxis
109	*Sparaxis pillansii* L.Bolus	
110	*Sparaxis tricolor* (Schneev.) Ker Gawl.	Sparaxis
111	*Sutherlandia frutescens* (L.) R.Br.	Cancer bush
112	*Tecoma capensis* (Thunb.) Lindl.	Cape honeysuckle
113	*Tetragonia decumbens* Mill.	
114	*Thunbergia alata* Sims	Black-eyed-susan
115	*Trachyandra divaricata* (Jacq.) Kunth	
116	*Tripteris clandestina* Less. (= *Osteospermum clandestinum* (Less.) Norl.	
117	*Tritonia crocata* (L.) Ker Gawl.	Blazing star
118	*Tritonia lineata* (Salisb.) Ker Gawl.	Pencilled tritonia
119	*Ursinia anthemoides* (L.) Poir.	Ringed rock ursinia
120	*Wahlenbergia capensis* (L.) A.DC.	Cape bell flower
121	*Watsonia borbonica* (Pourr.) Goldblatt and hybrids	
122	*Watsonia marginata* (L.f.) Ker Gawl.	Fairy watsonia
123	*Watsonia versfeldii* J.W.Mathews & L.Bolus	
124	*Zaluzianskya divaricata* (Thunb.) Walp.	

5.10 Microbes

Still under discussion.
